# Barriers and Facilitators for Sexual Trauma Disclosure in Boys and Men: A Systematic Review

**DOI:** 10.1177/15248380251325210

**Published:** 2025-03-23

**Authors:** Vita Pilkington, Sarah Bendall, Simon Rice, Michael Salter, Michael J. Wilson, Zac Seidler

**Affiliations:** 1 The University of Melbourne, Melbourne, VIC, Australia; 2 Orygen, VIC, Melbourne, Australia; 3 Movember Institute of Men’s Health, Melbourne, VIC, Australia; 4 University of New South Wales, Sydney, NSW, Australia

**Keywords:** help seeking/reporting/disclosure, child maltreatment, sexual abuse, child maltreatment, sexual assault, male victims

## Abstract

Disclosing sexual trauma can support recovery and healing for victim-survivors. Despite evidence indicating low disclosure rates and long disclosure delays in sexual trauma-exposed boys and men, little is known about factors impacting disclosure in this group. A systematic review was conducted to consolidate evidence surrounding barriers, facilitators, and predictors of disclosure likelihood and timing in boys and men following sexual trauma (i.e., sexual abuse, assaults, and coercion). Disclosure included formal reporting, help-seeking, and discussions with social supports. Four electronic databases (PsycINFO, CINAHL, SCOPUS, and Medline) were searched and 69 articles (42 qualitative, 25 quantitative, two mixed-methods) were included, representing 10,517 sexual trauma-exposed boys and men and 297 supports of sexual trauma-exposed boys and men (e.g., police, mental health practitioners). Barriers and facilitators were mapped according to levels of the social ecology. Boys’ and men’s understanding of, and responses to, sexual trauma were informed by perceived masculine norm violations and minimal public acknowledgement and validation of their trauma. These factors were compounded by a dearth of appropriate supports and practitioner knowledge. Assessed predictors of disclosure outcomes were highly variable, indicating limited theoretical understanding of factors likely to impact disclosure and need for greater methodological rigor and integration of theory into this domain. Scholarship remains biased towards disclosure barriers, with little emphasis on strengths-based factors that support help-seeking. Findings highlight impacts of gender socialisation processes on long-term experiences of sexual trauma. Implications for practice, policy, and research are discussed, including need for greater awareness and support for sexual trauma-exposed boys and men.

## Introduction

Sexual trauma (ST) is a significant public health concern that impacts people of all genders (Stoltenborgh et al., 2011). While prevalence is highest among girls, women, and gender minorities, boys and men also report concerningly high rates of ST, and ST inflicts similar impacts on boys and men as observed among other genders (Depraetere et al., 2020; Mathews et al., 2017). ST-exposed boys and men are at risk of long-term deleterious mental health, physical health, relational, and occupational outcomes (Millegan et al., 2016; Petersson and Plantin, 2019). For some, the impacts of ST are fatal, evidenced by elevated risk of suicide ideation, attempts, and deaths (Easton et al., 2013; Nicholas et al., 2022).

General community prevalence data suggests child sexual abuse (CSA) is experienced by 6.2%–18.8% of boys (Moody et al., 2018; Segal & Gnanamanickam, 2024). At least 3.8% of men report ST in adulthood (Craner et al., 2015; Elliott, 2004), while lifetime ST estimates are as high as 24.8% (Smith et al., 2018). However, the scope of ST perpetrated against boys and men is particularly difficult to ascertain due to limited data, definitional inconsistencies, and under-reporting (Catton & Dorahy, 2024; Peterson et al., 2011).

Throughout this review, ST refers to sexual abuse, assaults, or coercion across the lifespan. This includes rape, attempted rape, unwanted sexual touching, or forcing a person to perform sexual acts via force, threat, or manipulation. Disclosure refers to the act of revealing or telling others about the experiences of ST (Alaggia et al., 2019; Lemaigre et al., 2017). While disclosure can take many forms (e.g., intentional or unintentional; communicated verbally, in writing, or via non-verbal cues; anonymous or non-anonymous), this review focuses on victim-survivors’ intentional disclosures to a range of supports, spanning social supports, formal authorities, and health practitioners.

Disclosing events is often described as critical for processing and recovery (Easton et al., 2015). When supportive responses are received, discussing these experiences can alleviate shame, challenge internalized blame narratives, facilitate access to supports, and promote acceptance and healing (Rapsey et al., 2020). Disclosure is best viewed as a dynamic *process* that occurs across the lifespan (Alaggia et al., 2019), where determinations about *if, when*, and *how* to disclose ST involves complex decision-making processes, weighing up possible costs and benefits within given contexts (McElvaney et al., 2012). Indeed, for some, disclosing ST is not safe and can risk retaliation from perpetrators and harmful reactions from disclosure sources, such as disbelief, attributing blame, encouraging silence, and physical violence (Collin-Vézina et al., 2015; Elliott et al., 2022). Understanding factors that influence and underlie disclosure decisions is critical for identifying risk and protective factors for disclosure, identifying sub-populations vulnerable to non-disclosure, and informing strategies for promoting and supporting safe disclosure experiences (Zinzow et al., 2022).

Studies and reviews surrounding factors impacting ST disclosure commonly synthesize self-reported barriers and facilitators for disclosure (e.g., Lemaigre et al., 2017; Morrison et al., 2018). Increasingly, when considering barriers and facilitators for ST disclosure, researchers have advocated for socioecological models that take into consideration individual, relational, and sociocultural influences on disclosure decisions (Alaggia et al., 2019; Collin-Vézina et al., 2015; Zinzow et al., 2022). In addition to self-reported barriers and facilitators of disclosure, studies have also examined predictors of disclosure via quantitative methods (e.g., Latiff et al., 2024). Reviews indicate disclosure likelihood is lower among those closely related to perpetrators, younger at first ST exposure, and exposed to penetrative assaults (e.g., Alaggia et al., 2019). Known disclosure barriers include concerns about perpetrator retaliation, being disbelieved, and self-blame, while facilitators include trusted social supports and direct questioning about ST (e.g., Alaggia et al., 2019; Lemaigre et al., 2017). However, currently available reviews rely on mixed-gender (e.g., Alaggia et al., 2019; Lemaigre et al., 2017) and women-only samples (e.g., Gilligan & Akhtar, 2006; Stoner & Cramer, 2019) and fail to take into account the unique nature of boys’ and men’s experiences (PettyJohn et al., 2023).

Boys and men are less likely to disclose to social supports, police, and mental health practitioners, relative to girls and women (O'Gorman et al., 2023; Rice et al., 2022). While the current evidence base is limited, estimates suggest boys and men delay disclosure for upwards of 15–20 years on average (Easton, 2013; Romano et al., 2019). These findings are thought to be explained by gender socialization processes (Depraetere et al., 2020; O'Gorman et al., 2023; PettyJohn et al., 2023). ST has been described as eliciting significant feelings of shame for boys and men, partially due to perceptions that ST “violates” traditional masculine norms of physical strength, dominance, stoicism, self-reliance, agency, and heterosexuality, given these experiences inherently involve feelings of fear, powerlessness, and lack of control (Langdridge et al., 2023; PettyJohn et al., 2023). The tension between traditional masculine norms and ST is reflected in various gender-related barriers to disclosure reported by boys and men, such as not wanting to be labeled weak, gay, or not ‘real men’ (Kia-Keating et al., 2005; Ralston, 2020). These barriers sit within the context of boys’ and men’s comparative absence in ST research (O'Gorman et al., 2023), and harmful myths surrounding their victimization (Turchik & Edwards, 2012). Well-established “male rape myths” include beliefs that boys and men cannot experience ST, only gay boys and men experience ST, boys and men are unaffected by ST, and physiological arousal necessarily indicates consent to participate in sexual activity (Hine et al., 2021; Struckman-Johnson & Struckman-Johnson, 1992; Turchik & Edwards, 2012).

Despite increasing literature investigating boys’ and men’s experiences of ST, including impacts of gender socialization processes (Langdridge et al., 2023; PettyJohn et al., 2023), evidence surrounding factors impacting disclosure in this population has yet to be synthesized. This is a stark omission from the literature, particularly given the risk for non-disclosure, delayed disclosure, and premature loss of life associated with ST in boys and men (O'Gorman et al., 2023; Rice et al., 2022). This systematic review aims to address this gap in the literature by addressing the research question *what factors impact ST disclosure in boys and men?.* This includes factors impacting *whether* and *when* disclosure occurs. The review aims to consolidate existing evidence, assess the quality of evidence, and identify future research directions.

## Method

This review followed Preferred Reporting Items for Systematic Reviews and Meta-Analyses (PRISMA) guidelines (Moher et al., 2009; Page et al., 2021). After ensuring no similar reviews existed, a review protocol was registered on PROSPERO (CRD42022364930).

### Eligibility Criteria

Quantitative, qualitative, and mixed-methods studies examining factors impacting ST disclosure were included for review. This included reported barriers to, and facilitators of, disclosure, and variables associated with disclosure likelihood and disclosure timing. ST included sexual abuse, assaults, or coercion at any time, including childhood sexual abuse (CSA; <18 years), during adulthood, and where age of exposure was not specified. Disclosure was characterized as telling others about lived experiences of ST, including informal discussions with social supports (e.g., family, friends, partners), formal reporting to police, legal professionals, child protection agencies, and help-seeking from medical practitioners, mental health practitioners, sexual assault services, and other health professionals. Eligible participants included ST-exposed boys and men, and people who supported them, including police, mental health practitioners, sexual assault workers, and informal supports (see Supplementary File A for inclusion criteria). As per previous reviews (e.g., Kim et al., 2023; Tener & Murphy, 2015), facilitators included factors *supporting* and *motivating* disclosure.

### Data Sources and Search Strategy

Four databases (PsycInfo, Scopus, CINAHL, MEDLINE) were searched in October 2022 and updated in June 2024. The search strategy and search terms, including subject headings, were developed in consultation with a university librarian (see Supplementary File B for search terms). The search terms were devised following engagement with relevant background literature, in consideration of the varied terms used to refer to sexual trauma (e.g., sexual assault, sexual abuse, rape) and contexts in which intentional disclosures were likely to take place (e.g., police reporting, therapy, service engagement). Additional studies were sought via manual examination of reference lists, cross-checking against relevant reviews, and searching the first five pages of Google Scholar.

### Study Selection, Data Extraction, and Synthesis

Screening and duplicate removal were performed using the Covidence systematic review platform. Title and abstract screening were conducted by one reviewer and a second reviewer screened 20% of titles and abstracts. Given minimal disagreement (<10%), one reviewer assessed the remaining studies at the full-text level. Disagreements and uncertainties were resolved via discussion.

Data was extracted into Covidence by one author and then exported to Excel for subsequent synthesis. Extracted data included authors, publication year, location, design, sample size, demographics, trauma-related characteristics (e.g., childhood vs. adulthood ST), disclosure sources, and key findings. Given subtle variations between quantitative and qualitative outcomes (e.g., predictors vs. self-reported barriers of disclosure), results were synthesized across quantitative and qualitative findings separately, as per Seidler and colleagues (2016).

#### Quantitative Articles

Quantitative outcomes included: (a) the prevalence of boys and men that endorsed disclosure barrier items, (b) variables associated with disclosure likelihood, and (c) variables associated with disclosure timing. For quantitative outcome (a), barriers assessed in each study were grouped thematically into categories that reflected similar meanings between items. For outcomes (b) and (c), variables significantly associated with disclosure outcomes have been termed “predictors,” as per Latiff et al. (2024). Authors were contacted for clarification when findings were unclear or missing. Results were summarized using narrative synthesis; meta-analysis and assessment of publication bias were not feasible given differences in methodologies, analyses, and variables across studies.

#### Qualitative Articles

Qualitative outcomes included barriers to, and facilitators of, disclosure, as reported by ST-exposed boys and men and their supports. Qualitative data (themes, sub-themes, and quotes) were extracted from each study, and findings were synthesized via initial familiarization with published qualitative data, generating initial themes and sub-themes based on shared meanings in the data, refining themes, and summarizing the generated results for readers (Braun & Clarke, 2012). Reported barriers and facilitators were mapped according to socioecological frameworks (Bronfenbrenner, 1979). Bronfenbrenner’s (1979) socioecological model, which originated from developmental psychology, has recently been advocated for within disclosure literature (Alaggia et al., 2019; Collin-Vézina et al., 2015; Zinzow et al., 2022). Mapping barriers and facilitators using this framework facilitated recognition of contextual factors impacting disclosure (i.e., roles of relational, service-related, and sociocultural factors), which prevented viewing disclosure from only an individual responsibility lens (Zinzow et al., 2022).

### Risk of Bias and Quality Assessment

Quantitative articles were assessed using the Joanna Briggs Institute (JBI) checklist for analytical cross-sectional studies (JBI, 2020) and the Mixed-Methods Appraisal Tool (MMAT; Hong, 2018). The JBI checklist was used for articles reporting predictors of disclosure likelihood and timing, and the MMAT was used for quantitative articles that reported endorsed disclosure barriers and mixed-methods articles. The Critical Appraisal Skills Program (CASP) 2018 checklist for qualitative research (CASP, 2018) was used to appraise qualitative evidence. One reviewer (VP) conducted all quality assessments, with feedback from co-reviewers as needed.

### Protocol Deviations

As noted on PROPSERO, to ensure the review directly addressed its central research question, the protocol was amended to exclude articles that reported the prevalence of disclosure in ST-exposed boys and men without also reporting factors impacting disclosure.

## Results

### Study Selection and Characteristics

**Figure 1.**
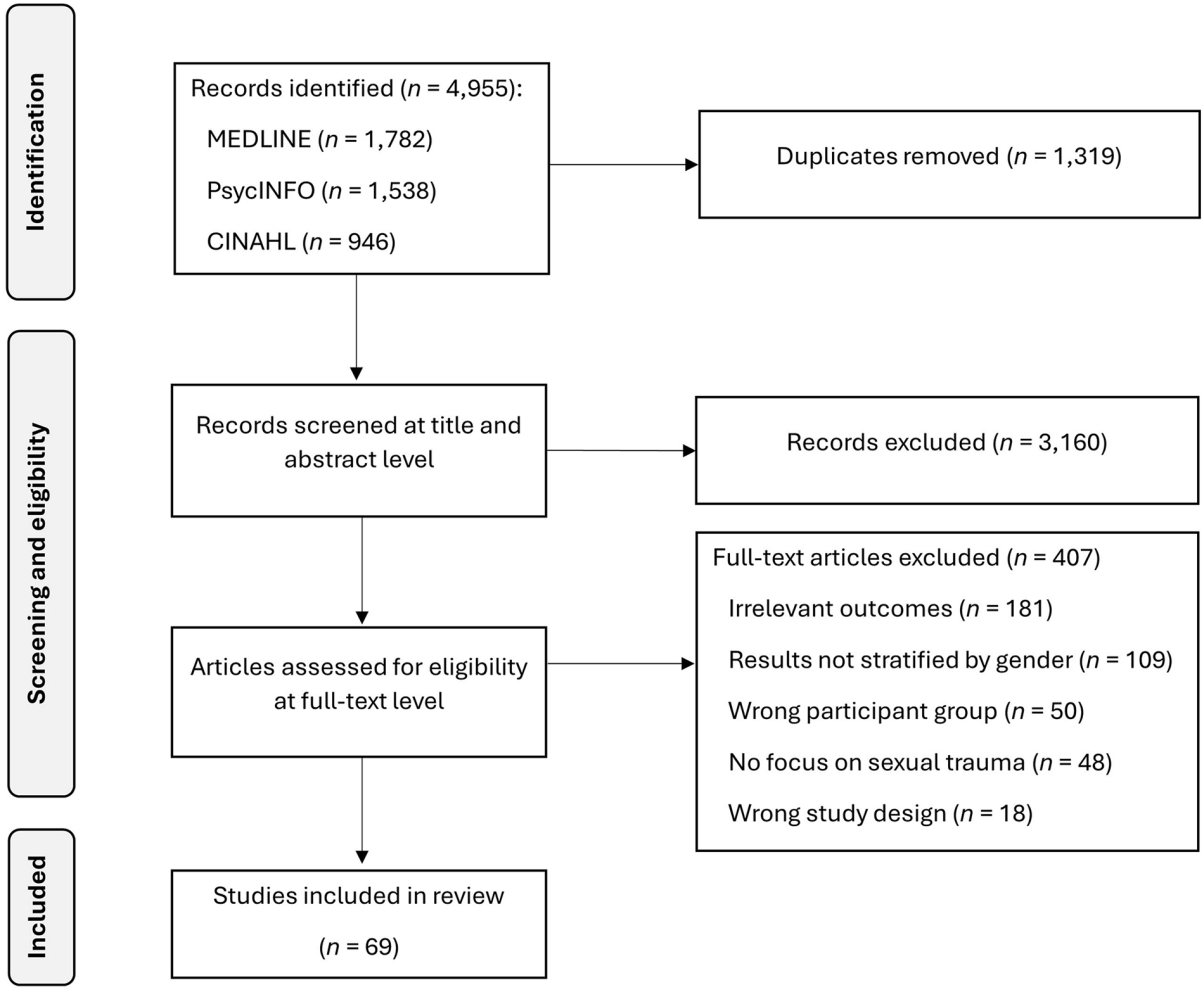
PRISMA Flow Diagram. PRISMA = Preferred Reporting Items for Systematic Reviews and Meta-Analyses.

The database search returned 4,955 articles, of which 1,319 were duplicates. At the title and abstract level, 3,636 were screened for inclusion and 476 progressed to full-text screening. Following screening, 69 articles met full inclusion criteria and were included in the review (Figure 1).^1^

Among the 69 included articles, 60.9% (*k* = 42) represented qualitative studies, 36.2% (*k* = 25) used quantitative designs, and the remaining 2.9% (*k* = 2) used mixed-methods designs. Data came from 23 countries across six continents (mostly the United States, *k* = 22, Canada, *k* = 9, and the United Kingdom, *k* = 8) and 42.0% (*k* = 29) were published in the last 5 years. The review included *n* = 10,517 ST-exposed boys and men and *n* = 297 people who acted as supports for ST-exposed boys and men. Boys’ and men’s ages ranged between 3 and 84 years, although most were adults (*M* = 32.3 years). Among the included articles, 59 included ST-exposed samples (*k* = 24 mixed gender, *k* = 35 all boys and men), four included support worker samples, and six included samples with both ST-exposed people and their sources of support. Support workers included mental health practitioners (e.g., Gruenfeld et al., 2017; Sivagurunathan, Orchard, MacDermid, et al., 2019), police (Jamel, 2010; Jamel et al., 2008), and samples where various supports were grouped (e.g., Chynoweth et al., 2020; Reeves & Stewart, 2017).

Characteristics of all included studies are shown in Table 1, including study methodology, participant demographics, age of ST exposure, and disclosure sources examined in each study. As presented in Table 1, most investigated CSA (52.2%, *k* = 36), followed by ST across the lifetime (29.0%, *k* = 20), during military service (7.3%, *k* = 5), in the 12 months prior to data collection (2.9%, *k* = 2), during adulthood (2.9%, *k* = 2), after specific ages (2.9%, *k* = 2), during university (1.4%, *k* = 1), and in the 6 months prior to data collection (1.4%, *k* = 1).

**Table 1.** Characteristics of *k* = 69 Included Articles.

Authors, Year; Location	Participant Type	ST Age	Study Type; Method	Sample Size; Gender; Age Range, Mean Age	Disclosure Type (Sources)	Outcome(s)
Adejimi et al., 2016; Nigeria	ST-exposed students (university)	Past 12 months	Quantitative; cross-sectional survey	*n* = 1,538; mixed gender (*n* = 640 boys and men); range = NR, *M* = 21.87 years	Reporting (university authority, police), help-seeking (medical practitioners), or social supports	B/F
Attrash-Najjar et al., 2023; Israel	ST-exposed men (submitted narratives to CSA Public Inquiry)	Childhood	Qualitative; analysis of written narratives	*n* = 51; all men; range = 18–60 years, *M* = 35 years	Any	B/F
Alaggia, 2005; Canada	ST-exposed members of general community	Childhood	Qualitative; interviews	*n* = 30; mixed gender (*n* = 11 men); range = 18 - 65 years, *M* = 40.1 years	Any	B/F
Aspin et al., 2009; New Zealand	ST-exposed Māori MSM from general community	Lifetime	Qualitative; interviews	*n* = 8; all men; range = 24 - 50 years, *M* = NR	Any	B/F
Boudreau et al., 2018; Kenya	ST-exposed MSM from general community	Childhood	Quantitative; cross-sectional survey	*n* = 489; mixed gender (*n* = 185 boys and men); range = 13 - 24 years, *M* = 18.4 years	Any	DL
Braun et al., 2009; New Zealand	ST-exposed MSM from general community	Lifetime	Qualitative; interviews	*n* = 19; all men; range = 20–54 years; mean NR	Any	B/F
Broban et al., 2020; 7 countries across African continent^1^	ST-exposed sexual assault service users	Lifetime	Quantitative; analysis of archival data	*n* = 13,550; mixed gender (n = 1,009 boys and men); range and mean NR	Help-seeking (sexual assault services)	B/F^2^; DT
Canan et al., 2023; United States	ST-exposed members of general community	Lifetime	Quantitative; cross-sectional survey	*n* = 2,013; mixed gender (*n* = 232 boys and men); range and mean NR	Informal (family members)	DL
Cashmore et al., 2017; Australia	ST-exposed people identified from crime statistics	Childhood	Quantitative; analysis of archival data	*n* = 122,757; mixed gender (number of boys NR); range and mean NR	Reporting (police)	DT
Christian et al., 2011; Democratic Republic of Congo	ST-exposed men in conflict and post-conflict villages, social supports (family, friends), and support workers (health practitioners)	Lifetime	Qualitative; interviews and focus groups	*n* = 30; mixed gender (*n* = 7 ST-exposed men); range = 25–74 years, mean NR	Any	B/F
Chynoweth et al., 2020; Italy, Bangladesh and Kenya	ST-exposed refugees and support workers (aid workers, human rights experts)	Lifetime	Qualitative; interviews and focus groups	*n* = 458 (*n* = 148 support workers and *n* = 310 refugees); mixed gender (number of ST-exposed boys and men NR); range and mean NR	Help-seeking (sexual assault services or medical practitioners)	B/F
Corboz et al., 2023; Afghanistan	ST-exposed men in general community, support workers (medical practitioners, mental health practitioners, community health workers)	Lifetime	Qualitative; interviews	*n* = 97 (*n* = 44 healthcare providers, *n* = 26 community health workers, *n* = 27 ST-exposed men); range = 18–40 years; *M* = 25 years	Help-seeking (medical or mental health practitioners)	B/F
Coxell et al., 2000; United Kingdom	ST-exposed genitourinary clinic service users	Lifetime	Quantitative; cross-sectional survey	*n* = 37; all men; range NR, *M* = 31 years	Any	DL
Donne et al., 2018; United States	ST-exposed members of general community	Past 12 months	Qualitative; interviews and focus groups	*n* = 32; all men; range = 21–47 years, *M* = 32 years	Help-seeking (sexual assault services, mental health practitioners, or medical practitioners)	B/F
Easton et al., 2014; United States	ST-exposed members of general community	Childhood	Qualitative; open-ended responses from cross-sectional survey	*n* = 460; all men; range = 19–84 years, *M* = 50.7 years	Any	B/F
Easton; 2013; United States	ST-exposed members of general community	Childhood	Quantitative; cross-sectional survey	*n* = 487; all men; range = 19–84 years, *M* = 50.4 years	Any	DL; DT
Eisenberg et al., 2021; United States	ST-exposed students (university)	Lifetime	Quantitative; cross-sectional survey	*n* = 551; mixed gender (*n* = 74 boys and men); range and mean NR	Reporting (police, university), help-seeking (medical practitioners), or social supports	DL
Elder, Domino, Rentz, et al., 2017; United States	ST-exposed veterans	During military	Qualitative; interviews	*n* = 21; all men; range = 29–70 years; mean NR	Any	B/F
Forde and Duvvury, 2017; Republic of Ireland	ST-exposed rape crisis center users	Childhood	Qualitative; interviews	*n* = 5; all men; range = 28–56 years; *M* = 44.6 years	Help-seeking (counselling from rape crisis centers)	B/F
Foster, 2017a; United States	ST-exposed mental health service users	Childhood	Qualitative; analysis of written narratives	*n* = 19; all boys; range = 3–17 years, *M* = 8.5 years	Any	B/F
Foster, 2017b; United States	ST-exposed mental health service users	Childhood	Qualitative; analysis of written narratives	*n* = 19; all boys; range = 3–17 years, *M* = 8.5 years	Any	B/F
Frias and Erviti, 2014; Mexico	ST-exposed students (public high school)	Childhood	Quantitative; cross-sectional survey	*n* = NR; mixed gender (*n* = NR boys and men); range = NR, *M* = 16.5 years	Any	B/F
Gagnier and Collin-Vezina, 2016; Canada	ST-exposed sexual assault service users	Childhood	Qualitative; interviews	*n* = 17; all men; range = 19-67 years, *M* = 47 years	Any	B/F
Gagnier et al., 2017; Canada	ST-exposed sexual assault service users	Childhood	Qualitative; interviews	*n* = 17; all men; range = 19–67 years, *M* = 47 years	Help-seeking (mental health practitioners, sexual assault services, support and advocacy groups) or reporting (legal professionals)	B/F
Gill and Begum, 2023; United Kingdom	ST-exposed British South Asian users of sexual assault services/websites	Childhood	Qualitative; interviews	*n* = 8; all men; range and mean NR	Any	B/F
Gruenfeld et al., 2017; International	Support workers (mental health practitioners)	Childhood	Qualitative; interviews	*n* = 9; all men; range and mean NR	Any	B/F
Guerra et al., 2021; Chile	ST-exposed sexual assault service users	Childhood	Mixed methods; interviews, analysis of clinical case notes^3^	*n* = 10, mixed gender (*n* = 3 men), range = 18–20 years, *M* = 18.8 years	Any	B/F
Gundlapalli et al., 2019; United States	ST-exposed veterans	During military	Quantitative; analysis of medical records	*n* = 1,730; mixed gender (*n* = 112 men); range and mean NR	Help-seeking (Veterans’ Health Administration)	DT
Hahn et al., 2021; United States	ST-exposed veterans	During military	Quantitative; cross-sectional survey	*n* = 1,185; mixed gender (*n* = 389 men); range = NR, *M* = 50.09 years	Help-seeking (Veterans’ Health Administration)	B/F
Hanson et al., 2003; United States	ST-exposed members of general community	Childhood	Quantitative; cross-sectional survey	*n* = 326; mixed gender (*n* = 71 boys); range = 12-17 years, mean NR	Any	DL
Hershkowitz et al., 2005; Israel	ST-exposed people identified from archival data (investigative interviews post-suspected ST)	Childhood	Quantitative; analysis of forensic interviews	*n* = 10,988; mixed gender (*n* = 3,416 boys); range = 3-14 years, mean NR	Reporting (investigative interviewers)	DL
Hietamäki et al., 2024; Finland	ST-exposed students (school)	Childhood	Quantitative; cross-sectional survey	*n* = 537; mixed gender (*n* = 79 boys); range = 11–17 years; mean NR	Any	DL
Hlavka, 2017; United States	ST-exposed people completing forensic interviews post-suspected ST	Childhood	Qualitative; interviews	*n* = 31; all boys; range = 5-17 years, mean NR	Any	B/F
Holland and Cipriano, 2021; United States	ST-exposed students (university)	During university	Qualitative; interviews	*n* = 40; mixed gender (*n* = 7 men); range = 19–25 years, *M* = 19.77 years	Reporting (university service)	B/F
Hunter, 2011; Australia	ST-exposed members of general community	Childhood	Qualitative; interviews	*n* = 22; mixed gender (*n* = 9 men); range = 25–70 years, mean NR	Any	B/F
Jackson et al., 2017; United States and Canada	ST-exposed MSM from general community	Adulthood	Qualitative; interviews	*n* = 18; all men; range NR; *M* = 42.4 years	Any	B/F
Jamel et al., 2008; United Kingdom	ST-exposed sexual assault service users (in Australia) and police (in United Kingdom)	Lifetime	Qualitative; open-ended responses from cross-sectional survey	*n* = 95; ST-exposed: *n* = 76, mixed gender (*n* = 20 men), range and mean NR; police: *n* = 19, gender NR, range and mean NR	Reporting (police)	B/F
Jamel, 2010; United Kingdom	ST-exposed sexual assault service users (in Australia) and police (in United Kingdom)	Lifetime	Qualitative; open-ended responses from cross-sectional survey	*n* = 95; ST-exposed: *n* = 76, mixed gender (*n* = 20 men), range and mean NR; police: *n* = 19, gender NR, range and mean NR	Reporting (police)	B/F
Javaid, 2018; United Kingdom	Support workers (counsellors, therapists, voluntary agency case workers, police)	Lifetime	Qualitative; interviews and open-ended responses from cross-sectional survey	*n* = 70; mixed gender (*n* = 33 men); range and mean NR	Any	B/F
Kwon et al., 2007; South Korea	ST-exposed military members	During military	Mixed methods; interviews and cross-sectional survey^4^	*n* = 75; all men; range and mean NR	Reporting (military superiors)	B/F
Lehrer et al., 2013; Chile	ST-exposed students attending General Education courses at a public university	After age 14	Quantitative; cross-sectional survey	*n* = 85; all boys and men; range = 13–30 years, mean NR	Reporting (police)	B/F
Manor-Binyamini and Schreiber-Divon, 2023; Israel	ST-exposed Bedouin men	Lifetime	Qualitative; interviews	*n* = 17; all men; range = 23–40 years; *M* = 31.88 years	Any	B/F
Masho and Alvanzo; 2010; United States	ST-exposed members of general community	Lifetime	Quantitative; cross-sectional survey	*n* = 91; all boys and men; range = NR, *M* = 42.4 years	Help-seeking (medical practitioners, mental health practitioners, or sexual assault services)	DL
Mgolozeli and Duma; 2020; South Africa	ST-exposed sexual assault service users (post-rape crisis centers)	Lifetime	Qualitative; interviews	*n* = 11; all men; range = 18–65, mean NR	Reporting (police)	B/F
Nofziger and Stein; 2006; United States	ST-exposed members of general community	Childhood	Quantitative; cross-sectional survey	*n* = 326; mixed gender (*n* = 71 boys); range = 12–17 years, mean NR	Any	DL
Okur et al., 2020; Netherlands	ST-exposed vocational school and university students	Childhood	Quantitative; cross-sectional survey	*n* = 586; mixed gender (*n* = 111 men); range = 18–25 years, *M* = 20.06 years	Help-seeking (medical practitioners, mental health practitioners) and social support (family, friends)	B/F
Oueis et al., 2024; Canada	ST-exposed MSM from general community	Adulthood	Qualitative; open-ended responses from cross-sectional survey	*n* = 206; all men; range = 18–77 years; *M* = 31.84 years	Any	B/F
Pacheco et al., 2023; International	ST-exposed men (incest and child trafficking/exploitation)	Childhood	Qualitative; interviews	*n* = 10; all men; range = 20–59 years; mean NR	Any	B/F
Patterson et al., 2023; New Zealand	ST-exposed members of men's support group for ST	Childhood	Qualitative; interviews	*n* = 9; all men; range = 42–67 years; mean NR	Any	B/F
Petersson and Plantin; 2019; Sweden	ST-exposed members of general community	Lifetime	Qualitative; interviews	*n* = 10; all men; range = "20s to 70s"; mean NR	Any	B/F
Postmus et al., 2015; Liberia	ST-exposed school students	Childhood	Quantitative; cross-sectional survey	*n* = 1,858; mixed gender (*n* = 1,100 boys); range and mean NR	Any	B/F
Priebe and Svedin; 2008; Sweden	ST-exposed students (high school)	Childhood	Quantitative	*n* = 1,493; mixed gender (*n* = 249 boys); range and mean NR	Any	DL
Priebe and Svedin; 2012; Sweden	ST-exposed students (high school)	Childhood	Quantitative; cross-sectional survey	*n* = 576; mixed gender (*n* = 112 boys and men); range = NR, *M* = 18.3 years	Any	DL
Rapsey et al., 2020; New Zealand	ST-exposed members of support group for ST	Childhood	Qualitative; interviews	*n* = 9; all men; range = 42–67 years, mean NR	Help-seeking (mental health practitioners)	B/F
Reeves and Stewart, 2017; Canada	ST-exposed Indigenous men attending health service and support workers at same service (mental health practitioners, community healers)	Lifetime	Qualitative; interviews	*n* = 16; ST-exposed: *n* = 6, all men, range = 30–60 years, mean NR; support workers: *n* = 10, mixed gender (*n* = 5 men), range="late 20s to late 60s", mean NR	Help-seeking (mental health practitioners)	B/F
Roberts, 2020; United Kingdom	ST-exposed sample of incarcerated men exposed to and convicted of CSA	Childhood	Qualitative; interviews	*n* = 18; all men; range = 23–67 years, mean NR	Any	B/F
Romano et al., 2019; United States and Canada	ST-exposed members of general community	Childhood	Quantitative; cross-sectional survey	*n* = 253; all men; range = 18–59 years, *M* = 39.5 years	Any	DT
Sharma, 2022; India	ST-exposed members of general community	Childhood	Qualitative; interviews	*n* = 11; all men; range = "20s to 50s"; mean NR	Any	B/F
Sivagurunathan, Orchard, MacDermid, et al., 2019; Canada	Support workers (mental health practitioners)	Childhood	Qualitative; interviews	*n* = 11; mixed gender (*n* = 4 men); range and mean NR	Any	B/F
Sivagurunathan, Orchard, and Evans, 2019; Canada	Support workers (mental health practitioners)	Childhood	Qualitative; interviews	*n* = 11; mixed gender (*n* = 4 men); range and mean NR	Help-seeking (mental health practitioners)	B/F
Sorsoli et al., 2008; United States	ST-exposed members of general community	Childhood	Qualitative; interviews	*n* = 20; all men; range = 24–61 years; mean NR	Any	B/F
Turchik et al., 2013; United States	ST-exposed veterans	During military	Qualitative; interviews	*n* = 20; all men; range = NR, *M* = 62.20 years	Help-seeking (mental health practitioners)	B/F
Velloza et al., 2022; Namibia	ST-exposed members of general community	Childhood	Quantitative; cross-sectional survey	*n* = 675; mixed gender (*n* = 101 boys and men); range = 13-24 years, mean NR	Any	B/F; DL
Walfield et al., 2024; United States	ST-exposed members of general community	Past 6 months	Quantitative; cross-sectional survey	*n* = 330; all men; range NR; *M* = 33.9 years	Reporting (police)	DL
Weare et al., 2024; United Kingdom	ST-exposed (forced-to-penetrate) members of general community	Lifetime	Qualitative; interviews	*n* = 30; all men; range NR; *M* = 42.9 years	Any	B/F
Weiss, 2010; United States	ST-exposed members of general community	Lifetime	Quantitative; cross-sectional survey	*n* = 1,050; mixed gender (*n* = 94 boys and men); range and mean NR	Police	DL
Widanaralalage et al., 2022; United Kingdom	ST-exposed members of general community	After age 13	Qualitative; interviews	*n* = 9; all men; range and mean NR	Any	B/F
Young, Pruett, and Colvin; 2018; United States	ST-exposed sexual assault helpline users	Lifetime	Qualitative; analysis of helpline calls	*n* = 116; mixed gender (*n* = 58 boys and men); range = 15–61 years, *M* = 32 years	Help-seeking (sexual assault services)	B/F
Zalcberg, 2017; Israel	ST-exposed members of Haredi (Ultra-Orthodox Jewish) community	Childhood	Qualitative; interviews	*n* = 40; all men; range = 18–44 years, *M* = 29 years	Any	B/F

*Note.* ST = Sexual trauma; MSM = men who had sex with men. Outcome codes: B/F = reported barriers and/or facilitators for disclosure; DL = variables associated with disclosure likelihood; DT = variables associated with disclosure timing. Sample sizes include the number of participants exposed to ST in each study (i.e., studies where general community samples were asked about ST exposure only list *n* = for those exposed to ST, given these are the participants relevant to the present review). ^1^ Full list of countries not provided. ^2^ Barriers to *timely* disclosures (more than 72 hr after ST). ^3^ Only quantitative results relevant to this review (values reported for quantitative results only). ^4^ Only qualitative results relevant to this review (values reported for qualitative component only).

### Quality Appraisals and Risk of Bias

Qualitative appraisal ratings (Supplementary File C) varied across studies; 44.9% of articles (*k* = 31) were rated as good quality with low risk of bias, 24.6% (*k* = 17) as fair quality with moderate risk of bias, and 30.4% (*k* = 21) as poor quality with high risk of bias. Methodological issues primarily related to quantitative articles and included non-representative samples, non-validated items/measures and limited attempts to measure and control for confounding variables in quantitative articles. In qualitative articles, minimal examination of possible impacts of researchers’ biases and positionality on the analysis and interpretation was identified.

### Quantitative Results

Quantitative results are reported across three outcomes: (a) prevalence of disclosure barrier item endorsement (*k* = 9), (b) predictors of disclosure likelihood (*k* = 15), and (c) predictors of disclosure timing (*k* = 5). ^2^ For outcomes (b) and (c), researchers used logistic regression, chi-square, Pearson’s correlation, Cuzick’s test for trend, and log linear analyses.

#### Endorsed Barriers to Disclosure

Nine studies provided ST-exposed boys and men with a list of potential disclosure barriers and reported the proportion that endorsed each barrier. After synthesizing items into similar categories/themes (see Supplementary File D), the most commonly assessed items enquired about concerns about unsupportive responses such as being disbelieved or blamed (e.g., “*I didn’t think anyone would believe me”*, “*Other people might think I caused the event to happen”*), shame (e.g., “*I felt ashamed and embarrassed”*, “*It would feel like an admission of failure”*), and logistical factors (e.g., “*The insurance did not cover any expenses”*, “*I found it too expensive”*). Endorsement of the barrier types varied widely between studies. For example, reporting of barriers surrounding concerns about unsupportive responses ranged from 1.6% (Okur et al., 2020) to 55.9% (Hahn et al., 2021).

Shame-related items were among the most widely endorsed disclosure barriers (Postmus et al., 2015; Velloza et al., 2022). For example, nearly two-thirds of veteran men described shame as impeding help-seeking following military ST (Hahn et al., 2021). In Okur et al. (2020), men rarely endorsed logistical barriers or concerns about being disbelieved, and instead mostly reported they did not need help (73.9%) or deemed disclosure unnecessary (46.9%). Items reflecting stoicism, minimization of ST, and a preference for self-reliance were also endorsed widely, by 23.9% (Okur et al., 2020) to 82.7% of participants (Kwon et al., 2007). Only Hahn et al. (2021) assessed men’s concerns about how others would view their sexualities following ST disclosure; this barrier was endorsed by approximately 60% of veteran men.

#### Predictors of Disclosure Likelihood

Predictors of disclosure likelihood were examined in 15 articles. Potential predictor variables comprised mostly demographic (e.g., race/ethnicity, sexuality) and trauma-related variables (e.g., perpetrator relationships, injuries sustained during ST). Findings are synthesized across these two domains below (Supplementary File E presents disclosure sources assessed, variables assessed as possible predictors, and statistical results).

##### Demographic Predictors of Disclosure Likelihood

Examined demographic variables included race/ethnicity, age, education, and sexuality. Race/ethnicity was only examined in Hanson et al. (2003), which found a significantly lower likelihood of disclosing (to any source) among African American vs. White boys, OR = 0.19, 95% CI: 0.04–0.96, *p* < .05. The two studies that examined age produced mixed findings, although age categorizations differed between studies. Hershkowitz et al. (2005) found increased formal reporting among older versus younger boys, although no significance testing was reported, while Easton (2013) found a significantly lower likelihood of reporting among older versus younger men, OR = 0.96, *p* < .01. Education was examined in terms of highest *completed* education level and *current* educational pathway. While Velloza et al. (2022) found disclosure (any source) was not associated with completed education, Walfield et al. (2024) found lower likelihood of police reporting in college-educated versus non-college-educated men, OR = 0.38, 95% CI: 0.19–0.77, *p* < .01. In the study that examined current education, Priebe and Svedin (2008) found higher disclosure likelihood (any source) in children attending academic versus vocational high schools, OR = 3.20, 95% CI: 1.65–6.21, *p* = .001. No differences were observed between heterosexual versus sexual minority boys and men in two studies (Eisenberg et al., 2021; Priebe & Svedin, 2012). In Canan et al. (2023), disclosure to family was higher in gay (16.9%) and heterosexual (14.6%), relative to bisexual men (1.8%), χ^2^ = 7.8, *p* = .020, Cramer’s *V* = 0.19, although the authors noted the small number of bisexual men in the sample (*n* = 24) limited this analysis.

##### Trauma-Related Predictors of Disclosure Likelihood

The most frequently assessed predictor of disclosure likelihood was perpetrator relationships, assessed in seven studies. Findings were mixed, although categorizations of perpetrator relationships varied between studies. For example, Easton (2013) examined family versus non-family perpetrated CSA, Hershkowitz et al. (2005) examined parent figure versus non-parent figure CSA, and Masho and Alvanzo (2010) examined ST perpetrated by family members or friends versus others. Overall, two studies found significantly lower disclosure likelihood in boys and men with close versus more distal relationships to perpetrators (i.e., family vs. non-family), *p*s < .05 (Easton, 2013; Hershkowitz et al., 2005), one found significantly higher disclosure likelihood with close relationships (i.e., family/friend vs. other) to perpetrators, aOR = 6.42, 95% CI: 1.47–28.04, *p* < .01 (Masho & Alvanzo, 2010), and three found no differences (Boudreau et al., 2018; Hanson et al., 2003; Walfield et al., 2024).

Other perpetrator characteristics assessed were clergy status and sex. Reporting was higher among men exposed to clergy-perpetrated versus non-clergy-perpetrated CSA, *p* < .001 (Easton, 2013), although this was only assessed in one study. In the four studies that assessed perpetrator sex, mixed results were observed. Hietamäki et al. (2024) found boys were less likely to disclose (to any source) when perpetrators were male versus female (*p* = .02). In contrast, Walfield et al. (2024) and Weiss (2010) observed less police reporting with female versus male perpetrators (*p*s < .05). The fourth study found no relationship between perpetrator sex and likelihood of disclosing to any source (Coxell et al., 2000).

Two studies (Nofziger & Stein, 2006; Walfield et al., 2024) examined relationships between age of ST exposure and disclosure, although they produced conflicting results. Nofziger and Stein (2006) reported lower likelihood of disclosure (any source) in boys and men who were older at first exposure, OR = 0.73, *p* < .01, but Walfield et al. (2024) found higher likelihood of police reporting with older age of exposure, OR = 1.79, 95% CI: 1.25–2.58, *p* < .01.

Three studies examined the relationship between disclosure and threats or injuries sustained during ST events. Findings were mixed for threats; Hanson et al. (2003) found no differences but Masho and Alvanzo (2010) found seven times greater likelihood of help-seeking among threatened vs. non-threatened men, aOR = 7.08, 95% CI: 1.52 – 33.03, *p* < .05, and Walfield et al. (2024) found 4.5 times greater likelihood of police reporting among men threatened with weapons (vs. no use of weapons) during assaults, OR = 4.54, 95% CI: 1.74–11.82, *p* < .001. Masho and Alvanzo (2010) also found significantly higher help-seeking likelihood among injured versus non-injured men, aOR = 6.58, 95% CI: 1.08–40.19, *p* < .05, although this was not observed in Walfield et al. (2024). No significant relationships were observed for level of exposure (e.g., single-event vs. repeated) or type of assault (e.g., penetrative vs. non-penetrative; (Boudreau et al., 2018; Hanson et al., 2003; Walfield et al., 2024).

##### Other Predictors of Disclosure Likelihood

Two studies showed relationships between disclosure to any source and social support variables. Those who engaged with their peers for support were three times more likely to disclose, relative to those who did not rely on peers, aOR = 3.03, 95% CI: 1.00–6.98, *p* = .04 (Velloza et al., 2022).^3^ Presence of caring and non-overprotective parent relationships positively predicted disclosure (relative to caring and overprotective, and non-caring and not overprotective parent relationships), *p*s < .05 (Priebe & Svedin, 2008).^4^

#### Predictors of Disclosure Timing

Eleven articles reported the time taken for boys and men to first disclose ST, which was mostly reported as mean time until disclosure (*k* = 4) and the proportion of CSA-exposed men who disclosed in childhood versus adulthood (*k* = 4). Mean disclosure timing ranged from 15.4 years (*SD* = 13.9, range NR; (Romano et al., 2019) to 21.38 years (*SD* = 14.88, range = 0–63; (Easton, 2013). Childhood disclosure ranged from 11.1% (Hunter, 2011) to 42.3% (Romano et al., 2019).

Among the articles that reported time taken to disclose, only five reported *predictors* of disclosure timing. Three examined time until boys and men disclosed to specific supports (e.g., police, sexual assault services (Broban et al., 2020; Cashmore et al., 2017; Gundlapalli et al., 2019) and two examined time until disclosure to any source (Easton, 2013; Romano et al., 2019). Findings are summarized below (see Supplementary File F for disclosure sources, disclosure timing conceptualizations, possible predictors examined, and statistical results).

##### Demographic Predictors of Disclosure Timing

Demographic variables examined included age (at data collection), race/ethnicity, relationship status, and level of education. In one study, age of CSA-exposed men (i.e., at the time of completing an online survey) was positively associated with number of years until first disclosure, *r* = .33, *p* < .001 (Easton, 2013). In Gundlapalli et al. (2019), no differences were observed across race/ethnicity, education, or relationship/marital status.

##### Trauma-Related Predictors of Disclosure Timing

Trauma-related variables assessed were age of ST exposure, perpetrator relationships, and type of assault. Although the two studies that examined age of exposure used different age categorizations (see Supplementary File F), both found delayed disclosure was more common in older versus younger participants (Broban et al., 2020; Cashmore et al., 2017), *p*s < .01. Easton (2013) found longer disclosure delays among CSA-exposed men with close relationships to perpetrators (i.e., family members), relative to those with more distal relationships, *p* < .05. No differences were found between clergy versus non-clergy-perpetrated CSA (Easton, 2013), although Cashmore et al. (2017) reported boys were particularly prone to long disclosure delays (>20 years) when perpetrators were authority figures. No significant relationships were found between disclosure timing and ST type or severity (Cashmore et al., 2017; Romano et al., 2019).

##### Other Predictors of Disclosure Timing

In one study, age at first disclosure was positively correlated with longer disclosure delays, *r* = .82, *p* < .01 (Romano et al., 2019). This study also found longer delays were associated with receiving positive reactions to disclosures, although this represented a weak association, *r* = .16, *p* < .05 (Romano et al., 2019). Weak positive associations were also found between longer disclosure delays and past 6-month externalizing difficulties, *r* = .15, *p* < .05, and substance use problems, *r* = .14, *p* < .05, while no significant relationships were observed between disclosure and internalizing problems or resilient functioning (Romano et al., 2019).

### Qualitative Results

**Figure 2.**
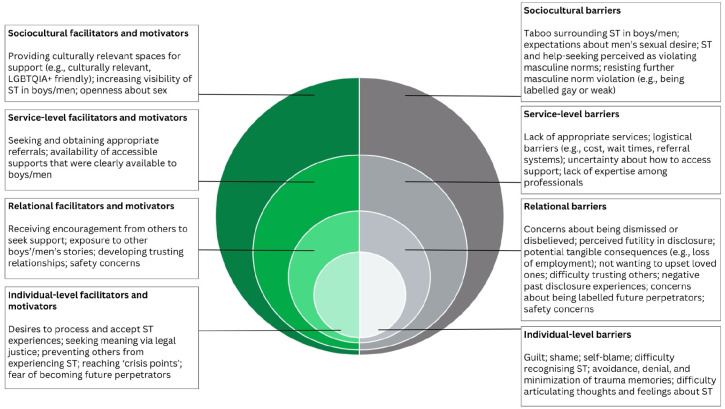
Results From Qualitative Studies: Barriers and Facilitators Mapped According to Socioecological Framework.

Most qualitative articles relied on interviews or focus groups (*k* = 23), followed by open-ended survey questions (*k* = 3), written narratives (*k* = 2), helpline calls (*k* = 1), or a combination of interviews and open-ended survey responses (*k* = 1). All included qualitative articles described disclosure barriers and 18 investigated factors that motivated and/or supported disclosures (see Supplementary File G for barriers and facilitators in each article). Figure 2 presents barriers and facilitators according to the socioecological framework (Bronfenbrenner, 1979), demonstrating various spheres of influence for disclosure. This schematic informed subsequent theme development. Five themes were developed to capture key meanings in the qualitative data: (a) coming to terms with ST, (b) relational push and pull factors for disclosure, (c) considering the safety to disclose, (d) needing appropriate services and pathways into care, and (e) perceiving incompatibility between masculinities and ST.

#### Theme 1. Coming to Terms With ST

Boys and men often needed to go through an internal process of understanding and reckoning with ST before disclosing. The theme *coming to terms with ST* describes internal factors that motivated boys and men to label their experiences as ST, and barriers that prevented internal recognition of these events. Some men expressed motivations to disclose to process, understand, and accept their experiences, often seeking understanding into long-standing impacts of CSA on their relationships, behavior, and mental health (e.g., Petersson & Plantin, 2019; Sorsoli et al., 2008). For example, in Rapsey et al. (2020, p. 2040) a participant explained “*I wanted to understand myself more, so that I could accept why I was [the way I was]*”. Other motivations included seeking recognition that their experiences were illegal or wrong, seeking justice (perpetrator punishment), and preventing others from experiencing ST (e.g., Mgolozeli & Duma, 2020; Widanaralalage et al., 2022). Conversely, some disclosure decisions appeared less intentional and were instead prompted by “crisis points,” including acute psychological distress, deteriorating mental ill-health, and substance use problems that necessitated immediate support (e.g., Alaggia, 2005; Sivagurunathan et al., 2019).

Recognizing and labeling ST events was described as a difficult, time-consuming process (e.g., Gagnier & Collin-Vézina, 2016; Patterson et al., 2023; Sivagurunathan et al., 2019), especially when physiological arousal was experienced during assaults (e.g., Sivagurunathan et al., 2019; Weare et al., 2024). Men found it difficult to “*admit or to become aware of the fact that [ST] has happened to you—to accept that you have been victimized, to accept that you have been impacted by it, let alone ask for help*” (Gruenfeld et al., 2017, p. 741). For some boys and men, this was made more challenging by legal definitions that did not acknowledge they could experience rape (Chynoweth et al., 2020; Weare et al., 2024). Many explained ST recognition was particularly difficult when perpetrators were women due to widespread beliefs that men exclusively constitute perpetrators, and women as victim-survivors (e.g., Roberts, 2020) and expectations that men constantly desire heterosexual sexual advances (e.g., Easton et al., 2014; Weare et al., 2024). Some MSM exposed to ST in adulthood described difficulties understanding experiences when sexual encounters with men had initially been consensual but became non-consensual over the course of the encounter (Aspin et al., 2009; Donne et al., 2018; Oueis et al., 2024).

Other commonly reported barriers were the avoidance, denial, and minimization of trauma memories, which were often repressed, minimized, or compartmentalized for long periods (e.g., Sorsoli et al., 2008; Turchik et al., 2013). Some explained they avoided discussing ST, including seeking therapeutic support, to avoid reliving painful thoughts and emotions surrounding the experience (Foster, 2017b) and avoidance often entailed alcohol and drug use (Elder et al., 2017; Forde & Duvvury, 2017). Self-blame narratives also created significant challenges for coming to terms with ST. Participants reported beliefs they had been personally culpable for ST events due to aspects of their character, such as being “weak” or “naïve,” and their behavior, such as indicating their interest in perpetrators and not physically fighting back during assaults (Oueis et al., 2024; Widanaralalage et al., 2022).

#### Theme 2. The Relational Push and Pull Factors for Disclosure

Theme two describes negative expectations about how others would respond to disclosures and roles others played in inviting disclosures. Related to theme one, others sometimes impeded boys’ and men’s recognition of ST events. For example, men who experienced CSA and exploitation described manipulation and deception from perpetrators and family members, which minimized their experiences (Pacheco et al., 2023). CSA-exposed men also expressed desire to protect perpetrators, including their relationships with perpetrators and perpetrators’ reputations and social standing in the community (Roberts, 2020; Sharma, 2022).

Boys and men frequently expressed concerns about unsupportive, dismissive responses to disclosure, such as disbelief, denial, and minimization (e.g., Sivagurunathan et al., 2019; Turchik et al., 2013). This was particularly evident in formal reporting contexts, where men described reporting as futile due to limited confidence in police/the criminal justice system to respond to their reports (e.g., Aspin et al., 2009; Jamel et al., 2008). Some stated their stories would be dismissed *because* of their gender (Widanaralalage et al., 2022), such as a participant in Mgolozeli and Duma (2020, p. 14), *“I just did not think police offers would [sic] listen to me. . . because everyone thinks rape is for women.”*

MSM often reported their stories would be dismissed because of both their gender and sexuality. This was due to stigmatizing attitudes among the general public, including perceived promiscuity of MSM and perceptions that they “deserved” to be assaulted (Donne et al., 2018; Jackson et al., 2017). In Jackson et al. (2017), MSM reflected on historical tensions between LGBTQIA+ communities and police, citing this contributed to difficulties in trusting and reporting to police. Further, those assaulted in adulthood explained their reports were unlikely to be believed and validated if they spent time with the perpetrator(s) prior to assaults occurring (e.g., engaged with them in public, gone home together; Braun et al., 2009; Oueis et al., 2024).

In several studies (e.g., Easton et al., 2014; Gruenfeld et al., 2017), boys and men reported concerns that disclosure would lead others to believe they (i.e., the person disclosing) would later perpetrate CSA, as per Gagnier and Collin-Vézina (2016, p. 230), “*If they knew I was sexually abused, they may think that I will sexually abuse their children.*” Other relational concerns included negative impacts on relationships and ostracization from peer groups and communities (e.g., Holland & Cipriano, 2021; Sivagurunathan, Orchard, & Evans, 2019). As discussed in theme five, this was often discussed in relation to concerns about being labeled gay and subject to homophobic responses (Sharma, 2022; Sorsoli et al., 2008).

Crucially, these concerns were not unfounded. Across qualitative studies, boys and men described harmful reactions from various supports, including family members, medical and mental health practitioners, and members of the criminal justice system (e.g., Corboz et al., 2023; Forde & Duvvury, 2017; Pacheco et al., 2023), which prevented subsequent disclosure. As per their fears, such reactions included minimization and outright denial of their experiences, being blamed, encouraging their silence, physical violence, and lack of action or follow-up (e.g., Attrash-Najjar et al., 2023; Gruenfeld et al., 2017; Jackson et al., 2017; Manor-Binyamini & Schreiber-Divon, 2023).

However, others also played critical roles in encouraging and supporting disclosure. Being asked directly about ST and encouraged to seek help—such as social supports encouraging mental health help-seeking or police reporting—were impactful facilitators for some men (e.g., Rapsey et al., 2020; Reeves & Stewart, 2017). Hearing others’ stories via support groups or in the media (e.g., television interviews, memoirs), particularly stories from other boys and men, was also a significant experience that helped many boys and men alleviate their shame and isolation and provided models for safe discussions about ST (e.g., Gagnier & Collin-Vézina, 2016).

#### Theme 3. Considering the Safety to Disclose

A number of safety concerns were discussed, many of which were relational in nature. These concerns both prevented and prompted disclosures. Some participants described concerns that family members would respond to disclosures with physical violence as punishment (e.g., Corboz et al., 2023; Roberts, 2020) or that perpetrators would respond with further physical and/or sexual violence (e.g., Guerra et al., 2021; Mgolozeli & Duma, 2020). At times, these fears were informed by direct threats and negative past disclosure experiences (e.g., Easton et al., 2014; Sivagurunathan et al., 2019). Conversely, in two studies, men explained that concerns about their welfare (i.e., further ST exposure or being harmed/killed by perpetrators) *motivated* them to report to police or child protection agencies (Hlavka, 2017; Mgolozeli & Duma, 2020).

Contextual factors impacted perceived safety to disclose; many men described limited cultural acceptance of homosexuality, with potential risks to acceptance and safety in the family or community if homosexuality was presumed or discovered (e.g., Attrash-Najjar et al., 2023; Sharma, 2022). Some participants felt their families would conflate their assaults with consensual same-sex sexual activity, which was likely to result in being labeled gay, bringing disrepute to, or “*letting down*” the family (Gill & Begum, 2023). Further, those in countries where same-sex relationships were outlawed feared legal penalties if they disclosed ST perpetrated by men (Chynoweth et al., 2020; Corboz et al., 2023).

Men described making decisions about whether to disclose, and who to disclose to, based on their perceived safety with various supports. In Gill and Begum (2023), CSA-exposed men reflected it was often deemed unsafe to disclose to family members as children, as this risked unsupportive responses, reputational damage, and feeling they “*let down*” their families, whereas disclosing to romantic partners as adults was deemed more likely to elicit supportive, empathetic responses. Concerns about letting down family members, bringing shame to the family unit, and ostracizing family members from the community were particularly prevalent in samples of men from non-western locations (e.g., Christian et al., 2011; Chynoweth et al., 2020; Corboz et al., 2023; Gill & Begum, 2023).

Finally, several men described “*power imbalances*” in therapy, where therapy was seen as unsafe due to its potential to mimic aspects of abuse (Sivagurunathan, Orchard, & Evans, 2019). This was described by a man in Rapsey et al. (2020, p. 2039), who explained that during therapy “*you go into a room with someone and the door is shut, there’s a parallel immediately with abuse*”.

#### Theme 4. Needing Appropriate Services and Pathways Into Care

The fourth theme *needing appropriate services and pathways into care* summarizes factors impeding access to support, mostly surrounding mental health care. Participants often described uncertainty about where and how to seek support (e.g., Turchik et al., 2013), which was complicated by the dearth of services and referral options available to boys and men (e.g., Chynoweth et al., 2020; Sivagurunathan, Orchard, & Evans, 2019). Some described this lack of supports in relation to the limited acknowledgement of men’s victimization (see theme five), “*We don’t have a cultural place for men as victims. . . If we don’t accept that a problem exists, why would anybody want to create services to address a non-existent problem?*” (Gruenfeld et al., 2017, p. 742). Some men even reported they had been turned away from sexual assault services after being presumed to be perpetrators based on their gender (Widanaralalage et al., 2022).

Coupled with these challenges were logistical barriers, such as long wait times, high financial costs, insurance coverage issues, scheduling difficulties, difficulty organizing transport to appointments, and poor communication between services (e.g., Donne et al., 2018; Sivagurunathan, Orchard, & Evans, 2019). For example, in Rapsey et al. (2020), men described requirements to disclose ST upon their first contact with mental health practitioners to access funded support for ST-related issues. This required disclosure to occur before developing trust with practitioners, which was deemed highly challenging. Further, both ST-exposed boys and men and mental health practitioners described a lack of practitioners with sufficient knowledge and training in gender-related ST experiences and support needs (e.g., Chynoweth et al., 2020; Corboz et al., 2023; Gruenfeld et al., 2017).

Two studies highlighted a lack of culturally relevant supports for ST-exposed boys and men (Aspin et al., 2009; Reeves & Stewart, 2017). Men reflected that support services were largely Western-centric and did not adequately consider boys’ and men’s cultural identities, including impacts of culture on support needs. ST-exposed Māori men from New Zealand (Aspin et al., 2009) and Indigenous men in Canada (Reeves & Stewart, 2017) explained that incorporating their cultures into care when accessing mental health support (e.g., promoting connection to culture, including cultural practices such as drumming ceremonies) was instrumental in supporting them to disclose and heal from ST. In a similar vein, MSM expressed a desire to speak with practitioners with understanding about their LGBTQIA+ identities and experiences (e.g., Donne et al., 2018; Jackson et al., 2017).

#### Theme 5. Perceiving Incompatibility Between Masculinity and ST

The final theme describes participants’ reports about the perceived incompatibility between masculine norms and ST disclosure. Experiencing ST was described as violating traditional masculine norms of strength and invulnerability, and the perceived failure to live up to these norms elicited significant shame among boys and men (e.g., Gruenfeld et al., 2017; Widanaralalage et al., 2022). As articulated in Easton et al. (2014, p. 463), “*Sexual abuse to a man is an abuse against his manhood as well*”. Several studies noted that boys’ and men’s desires to conceal these perceived violations and to maintain ‘successful’ masculine presentations impeded ST disclosure (e.g., Elder et al., 2017; Hlavka, 2017; Sorsoli et al., 2008).

Sexuality concerns were often implicated in this theme, where many participants described non-disclosure motivated by resisting being viewed/labeled as gay (e.g., Elder et al., 2017; Gruenfeld et al., 2017). This was reported by heterosexual men, who reported not wanting to be *mistakenly* labeled gay, and MSM, who reported not wanting their sexualities to be *attributed* to ST events (e.g., Easton et al., 2014). Boys and men often resisted being labeled gay given the potential for marginalization and unsupportive responses resulting from non-heterosexuality, such as mocking and homophobic comments (see theme two; Hunter, 2011; Sorsoli et al., 2008). MSM often reported that they already occupied marginalized masculine identities given their sexualities, and disclosing ST risked contributing to further marginalization (e.g., Widanaralalage et al., 2022).

In addition to non-disclosure resulting from beliefs that experiencing ST violated masculine norms, participants also reflected that help-seeking was unacceptable for boys and men (e.g., Sorsoli et al., 2008). Seeking support and expressing strong emotions (e.g., crying) during disclosure were described as violating the stoicism and self-reliance expected of men and reflecting weakness, and were therefore avoided (e.g., Guerra et al., 2021; Reeves & Stewart, 2017). In Donne et al. (2018), men explained that ideas surrounding how men should behave interacted with their cultural backgrounds, where Black men reflected that help-seeking, particularly for psychological or emotional issues, was not normalized or encouraged “As African American, I grew up, we are not taught to, ‘oh go get counselling for this’. No one talks about getting therapy. That’s not even a discussion. Only time you’re going to anything is when you have an illness, you’ve been hurt, like you got a cut” (p. 198).

The incompatibility between masculinity and ST was also discussed in the context of broader silence surrounding boys’ and men’s victimization. Many participants described limited acknowledgment of ST in boys and men; these experiences were described as “taboo” and elicited significant discomfort among the general public (Christian et al., 2011; Sorsoli et al., 2008). As a result, they sometimes described non-disclosure as a means of “*protecting*” others from the discomfort and pain associated with acknowledging and discussing these events (Gill & Begum, 2023; Hlavka, 2017; Zalcberg, 2017).

## Discussion

This is the first study to synthesize evidence regarding factors impacting ST disclosure in boys and men. The review offers a comprehensive summary of available evidence by consolidating findings from 69 quantitative, qualitative, and mixed-methods articles. Studies were conducted across a significant breadth of countries and a substantial portion (42.0%, *k* = 29) were published in the last 5 years , indicating this topic represents a growing and globally significant area of interest. Bronfenbrenner’s (1979) socioecological framework was used to conceptualize barriers and facilitators that impacted boys’ and men’s decisions to tell others about ST experiences (based on the qualitative evidence). As shown in Table 2, findings underscore that boys and men are subject to complex barriers that impede their access to support at all levels of the social ecology. These barriers are highly interrelated. Disclosure intentions and behaviors are informed by boys’ and men’s beliefs that ST “violates” traditional masculine norms, minimal acknowledgment of ST perpetrated against boys and men and culturally-held beliefs about the legitimacy of men’s victimization, anticipated reactions from others, and resourcing availability (Rapsey et al., 2020; Sorsoli et al., 2008). These intersecting factors contribute to boys’ and men’s internal beliefs about the feasibility and possible costs of disclosure (Gruenfeld et al., 2017). In contrast, results also highlighted several factors that facilitated and supported disclosure among boys and men, although facilitators were discussed in considerably fewer studies and in less depth, relative to disclosure barriers. Many facilitators reported in the extant literature emphasized important roles played by others in inviting and supporting boys and men to share their experiences (Gagnier et al., 2017). Further, while quantitative studies have sought to examine predictors of *whether* and *when* disclosure occurs, this represents an early evidence base with a number of important weaknesses. Primarily, this includes the need for greater methodological rigor, larger and more diverse samples, and integrating theoretical understanding regarding factors likely to impact disclosure.

**Table 2.** Critical Findings From Review.

Number	Details
Critical finding 1	Complex barriers to disclosure were experienced at all levels of the social ecology, which included significant systemic and sociocultural factors that impeded disclosure, formal reporting, and help-seeking. Some of these barriers are shared across genders but others appear unique to boys and men.
Critical finding 2	Difficulty understanding and disclosing ST was exacerbated by the minimal public acknowledgment of victimization experienced by boys and men.
Critical finding 3	Boys and men often felt that experiencing ST meant they had violated masculine norms requiring them to demonstrate dominance, strength, and control. This contributed to prolonged and significant feelings of shame, self-blame, isolation, and reticence to seek support. While this was reported across samples and backgrounds, cultural nuances were apparent, where boys and men in non-western contexts often described ST as risking bringing shame to their families, in addition to themselves.
Critical finding 4	Research showed a bias toward investigating barriers to disclosure, relative to factors that motivated and supported ST disclosure in boys and men. Reported facilitators largely highlighted critical roles others played in supporting boys’ and men’s disclosure via offering safe and trusting relational foundations for disclosure, direct questioning about sexual trauma, and encouraging help-seeking. Hearing other boys’ and men’s stories of ST was particularly powerful in combatting shame and isolation.
Critical finding 5	Limited quantitative research has been conducted. The available quantitative evidence has not been based on a theoretical understanding of ST disclosure. Further, the ability to determine whether particular groups of boys and men are at higher risk of non-disclosure and delayed disclosure is currently limited by important methodological issues within existing quantitative research, including non-sophisticated statistical approaches, use of non-validated measures, and small samples.

ST = Sexual trauma.

### Synthesis of Disclosure Barriers

Reticence towards disclosure was often discussed in relation to the lack of “a cultural place for men as victims” (Gruenfeld et al., 2017, p. 742), in light of traditional masculine norms that describe men as invulnerable, dominant, and hyper-sexual (PettyJohn et al., 2023). Boys and men frequently minimized and struggled to recognize ST experiences (Lehrer et al., 2013), in part because they viewed their experiences as “violating” norms, and as abnormal or unlikely, given their gender (Sivagurunathan et al., 2019). Difficulty recognizing ST has also been reported among mixed-gender samples and women (Collin-Vézina et al., 2015; Stoner & Cramer, 2019), which has informed educational programs and public health campaigns promoting increased knowledge and recognition of ST (Kemshall & Moulden, 2017). However, the effectiveness of these strategies for boys and men appears limited, in light of the persistent lack of awareness that this group *can* and *does* experience ST (O'Gorman et al., 2023). Difficulty labeling ST was evident across perpetrators genders, including among boys who were subjected to CSA by women (Roberts, 2020) and MSM who were assaulted by other men in adulthood (Donne et al., 2018). For some, difficulties labeling and understanding ST were informed by legislation that failed to recognize assaults perpetrated against boys and men (Christian et al., 2011; Chynoweth et al., 2020), which highlights the important roles of structural and systemic factors in shaping boys’ and men’s internal understandings of the legitimacy of their experiences. Many of these results mirror those from men exposed to intimate partner and domestic violence, who report difficulties identifying as victim-survivors because their experiences are deemed “atypical” and struggle to reconcile their experiences with their understandings of gender (Hine et al., 2022; Kim et al., 2023).

The tension between men’s victimization and traditional masculine norms leaves many ST-exposed boys and men faced with significant feelings of confusion, internalized blame, and shame (O'Gorman et al., 2023; Petersson & Plantin, 2019). Gendered manifestations of shame included grappling with masculine identities and beliefs others would view them as weak or no longer ‘real men’ (Christian et al., 2011; Ralston, 2020). This was evident across sexualities, although as noted by PettyJohn et al. (2023), may be particularly relevant to MSM, who often already believe they occupy marginalized masculine identities.

The perceived ‘loss’ of masculinity following trauma has been reported elsewhere and helps contextualize findings that following ST, some men show hypermasculine behaviors, including aggression and overt displays of misogyny, homophobia, and hypersexuality, in efforts to reaffirm masculine identities and presentations (Elder, Domino, Mata-Galán, et al., 2017; Gauthier-Duchesne et al., 2024; Ralston, 2020). To avoid *further* masculine norm violation, disclosure was avoided because this required vulnerability, reliance on others, and (potentially) strong displays of emotion, all of which further contradicted traditionally masculine expectations of self-reliance and stoicism (Easton et al., 2014).

Across contexts and disclosure sources, boys and men expressed concerns their reports would elicit disbelief, dismissal, and blame (Javaid, 2018; Zalcberg, 2017), similar to men exposed to domestic violence (Kim et al., 2023). While uncertainty and fear about others’ disclosure reactions are common across genders (Lemaigre et al., 2017), boys’ and men’s concerns existed in the context of widely held beliefs that ST is exclusively perpetrated against girls and women (Christian et al., 2011). This “female victim, male perpetrator” paradigm can lead boys and men to feel their experiences are denied, thereby instilling further confusion, shame, and isolation following ST (Stemple & Meyer, 2014).

Discussions surrounding sexualities were directly implicated in boys’ and men’s concerns about how others would respond (Alaggia, 2005). Indeed, it is well established that many men experience sexuality conflict and concerns about their sexualities being misconstrued following ST (Kia-Keating et al., 2005). This was evident across sexualities, where heterosexual men resisted being incorrectly labeled gay and MSM resisted their sexualities being incorrectly attributed to ST events (Easton et al., 2014). These concerns largely existed in the context of heterosexuality being a key facet of traditional masculinity (Cheng, 1999) and reflected pressure to uphold masculine norms (Turchik et al., 2013). However, for some, sexuality concerns were related to laws that prohibited same-sex sexual activity, which resulted in fears that reporting their experiences (when perpetrated by other men) may lead to receiving punishment, rather than support (Chynoweth et al., 2020; Corboz et al., 2023).

Regrettably, boys’ and men’s concerns were often justified, as their informal and formal supports were largely unprepared to validate their experiences. For many, concerns about telling others were founded on past experiences, as their reports had been met with disbelief, minimization, discouraging subsequent disclosures, and even joking about or celebrating the assaults perpetrated against them (Attrash-Najjar et al., 2023; Jackson et al., 2017; Mgolozeli & Duma, 2020). This is consistent with mental health practitioners’ reports that a central component of their roles when supporting ST-exposed men is managing expectations around disclosure and responding to distress when unsupportive responses are experienced (Widanaralalage et al., 2023). These findings are highly concerning, particularly given such responses can reduce the likelihood and hope for future help-seeking, and contribute to prolonged and deteriorating mental ill-health, including self-harm and suicide (Collin-Vézina et al., 2021; Orchowski & Gidycz, 2015).

Notably, even for boys and men who *wanted* to access help from police, sexual assault services, and mental health practitioners, structural barriers were frequently encountered, including a dearth of appropriate support and knowledge among police and health practitioners (Chynoweth et al., 2020; Sivagurunathan, Orchard, & Evans, 2019). Consequently, boys and men who are able to overcome complex disclosure barriers are likely to find few, if any, appropriate services and are at risk of engaging with practitioners who are minimally equipped to recognize and respond to their trauma (O'Gorman et al., 2023; Rice et al., 2022).

Taken together, these findings highlight that boys’ and men’s challenges with recognizing and telling others about ST experiences do not simply reflect knowledge gaps that can be addressed via education alone; these challenges are informed by their minimal recognition in criminal justice, servicing, and sociocultural domains, which requires structural and sociocultural action, as well as public health investment (Petersson & Plantin, 2019; PettyJohn et al., 2023).

#### An Intersectional Lens: Barriers Within Particular Groups of Boys and Men

The limited acceptance of boys and men as legitimate victim-survivors was particularly evident in non-western contexts, based on samples from Afghanistan (Corboz et al., 2023), India (Sharma, 2022), Democratic Republic of Congo (Christian et al., 2011), Israel (Manor-Binyamini & Schreiber-Divon, 2023; Zalcberg, 2017), Bangladesh, and Kenya (Chynoweth et al., 2020). Indeed, men in these settings emphasized that disclosure risked not only *personally* receiving unsupportive responses but also reputational damage for their families, and even ostracization from their local communities. Similarly, some participants described non-disclosure due to not wanting to “let down” or disappoint their families (Gagnier & Collin-Vézina, 2016; Gill & Begum, 2023). For these boys and men, the ramifications of disclosure extended to others within their personal networks, which played key roles in their decisions surrounding whether to share their experiences. Similar findings were recently reported in a rapid review by Widanaralalage et al. (2024). Further, as per previous research (e.g., Jacoby et al., 2020; Tillman et al., 2010), Black men in Donne et al. (2018) explained that help-seeking was not normalized in their communities, which prevented them from accessing mental health support in particular, following ST. In addition to the stigma surrounding mental ill-health and help-seeking, individuals from ethnic minority backgrounds also experience greater structural barriers to help-seeking, including reduced access to services and high costs as a barrier to entry (Alam et al., 2024).

While the number of studies that specifically sampled MSM was limited, findings from this review highlight further complications surrounding disclosure faced by this group, given their need to navigate homophobic attitudes and responses from others. Indeed, beliefs that their experiences would be dismissed due to being deemed sexually “*promiscuous*” and even “*deserving*” of assaults (Donne et al., 2018) emphasizes the double-stigma, and often the limited safety to disclose, among this group (Jackson et al., 2017). Further, some participants reported that practitioners often had a limited understanding about boys’ and men’s cultural, sexual, and gender identities and how this impacted their experiences of ST and support needs (e.g., Jackson et al., 2017; Oueis et al., 2024; Reeves & Stewart, 2017). These findings collectively indicate the complex intersections between race, culture, and gender in impacting the ramifications of boys’ and men’s disclosure of ST.

### An Enduring Emphasis on Disclosure Barriers

This review highlighted a number of facilitators for disclosure, which contribute towards understanding about how boys and men can overcome complex disclosure barriers, although the existing literature has emphasized barriers with less emphasis on facilitating and motivating factors, as per previous similar reviews (Alaggia et al., 2019; Heron & Eisma, 2021).

Commonly reported facilitators were largely relational and included the presence of trusted supports (Gagnier & Collin-Vézina, 2016), high-quality relationships with peers and parents (Priebe & Svedin, 2008; Velloza et al., 2022), developing trust with practitioners (Gruenfeld et al., 2017; Reeves & Stewart, 2017), and encouragement to seek support (Foster, 2017b). This aligns with previous reviews that emphasize the importance of having supports who are deemed likely to respond to disclosures with empathy, validation, and practical and emotional support (Alaggia et al., 2019). Other key facilitators included direct questioning about ST, offering culturally appropriate support, and hearing the stories of other boys and men (Reeves & Stewart, 2017). The power of hearing other boys’ and men’s stories of ST was highlighted by many participants, where these stories could come in various forms (e.g., peer support groups, discussion with social supports who shared similar experiences, and media outputs such as interviews and memoirs; Gagnier et al., 2017; Oueis et al., 2024; Zalcberg, 2017). This highlights the importance of normalizing ST as a means of reducing victim-survivors’ stigma, shame, and isolation (Kennedy & Prock, 2018).

Several studies shined a light on internal motivating factors for disclosure and help-seeking among boys and men, including desires to understand and process ST events and to address long-term deleterious impacts of ST (e.g., relationship difficulties, substance use problems, mental ill-health; Forde & Duvvury, 2017; Reeves & Stewart, 2017). These studies highlight that men *can* overcome barriers to disclosure, which often appear motivated by their desires to pursue healthier relationships and lives, and take back control by addressing long-term impacts of the trauma (Patterson et al., 2023; Ralston, 2020; Rapsey et al., 2020). Further evidence surrounding motivating and facilitating factors remains needed, with consideration of individual-level, relational, systemic, and sociocultural spheres of influence.

### Methodological Issues and Limited Theoretical Understandings of Disclosure in Quantitative Studies

A wide range of variables were assessed as possible predictors of disclosure outcomes, although each possible predictor was only assessed in a small number of articles. The lack of consistency in variables assessed indicates a limited theoretical understanding of factors *likely* to impact disclosure. Authors of the included articles reported that some variables were systematically associated with disclosure outcomes. For example, higher-risk groups were those assaulted by family members (Easton, 2013; Hershkowitz et al., 2005) and African American boys (Hanson et al., 2003). Some of these results have been supported elsewhere. For example, challenges disclosing ST with closely related perpetrators have been attributed to factors such as residing together, physical safety risks, potential disruptions to family dynamics, and love and desire to protect perpetrators (Lemaigre et al., 2017). However, other findings, such as poorer disclosure outcomes among those who were older at first ST exposure (Cashmore et al., 2017; Nofziger & Stein, 2006), contradicted previous reviews (Alaggia et al., 2019; Zinzow et al., 2022). There were a number of important methodological issues within the quantitative articles, including the use of small samples, non-validated measurement tools, and non-sophisticated statistical approaches (or incomplete reporting of statistical results). The inferences that can be drawn from this evidence are limited by moderate-to-high risk of bias in many quantitative articles, the limited number of articles examining each potential predictor, and wide methodological variability, precluding meta-analysis. As such, available evidence currently precludes definitive statements about *which* groups of boys and men are at the highest risk of non-disclosure and delayed disclosure. The themes derived from the qualitative studies in this review, which describe factors impacting disclosure at various levels of the social ecology, may inform decision-making about predictors of disclosure to be examined in future studies. Future meta-analyses are warranted as this evidence base continues to proliferate in size, quality, and consistency.

### Implications and Future Directions

Our findings inform implications for practice, policy, and research, shown in Table 3.

**Table 3.** Summary of Review Implications for Practice, Policy, and Research.

Area	Implications
Policy	• There is an urgent need to increase public awareness about the prevalence and impacts of ST among boys and men, particularly in higher-risk settings (e.g., institutional settings) and groups (e.g., MSM). • There is a clear need to adopt gender-neutral language in ST legislation to ensure enhanced recognition of assaults against boys and men. This requires amending legal language pertaining to sex characteristics when referring to non-consensual sexual acts (i.e., rape, assaults, coercion, abuse). • Increased investment, availability, and visibility of services and treatment models for ST-exposed boys and men require prioritization. While developing and upscaling supports designed specifically for boys and men will require time and investment, existing sexual trauma services would also benefit from including images of boys and men on relevant resources (e.g., websites, flyers) and clearly stating that boys and men are eligible for support from their services. • Development and implementation of trauma-informed, gender-sensitive training for police, sexual assault workers, and health practitioners is warranted to support effective recognition and responding to ST.
Clinical practice	• Health practitioners require training surrounding gender-specific ways ST may present. While further understanding of gender-specific impacts remains needed, this may include men describing feelings of emasculation or shame; isolation; difficulties connecting with other men; avoidant coping (including substance use problems); relationship problems; significant changes in sexual health, behavior, or interest; externalising symptoms and behaviors; and signs or descriptions of attempts to re-affirm traditional masculine tropes (e.g., internalized homophobia or hyper-sexuality). As with all clinical presentations, these possible indicators should be considered in the context of individual circumstances and case conceptualisations. • It is essential that mental health practitioners are effectively prepared to provide support that addresses gendered impacts of ST and to understand how boys’ and men’s cultural and sexual identities impact their support needs. This will likely include directly engaging with, and combatting, harmful male rape myths, highly ingrained trauma narratives and shame surrounding masculine norm violations, and feelings of responsibility surrounding ST events. It is recommended that clinicians are equipped with sufficient understanding about the widespread nature of ST in boys and men, factors underpinning boys’ and men’s silence surrounding ST, and long-term impacts of ST in this population. • Health practitioners are encouraged to ask boys and men about ST histories and be aware of their own potential biases surrounding sexual trauma, including gender-related assumptions and attitudes. Development of training modules for health practitioners and others who are likely to come into contact with ST-exposed boys and men (e.g., police and sexual assault service volunteers) may support best practices among practitioners. • Individuals offering support to boys and men who disclose ST histories have a responsibility to respond to these disclosures in a non-judgmental, compassionate manner. They should demonstrate through their words, body language, and actions that boys and men can receive affirming and safe responses when they choose to tell others about ST experiences, which sets the foundation for subsequent disclosures. Mental health practitioners should be mindful that men may be prone to minimizing ST events and their impacts. As such, they may need to consider ways the trauma continues to impact their clients’ lives and support clients to recognize these ongoing impacts. • Clinicians should be prepared to engage with ST-exposed boys’ and men’s social supports (e.g., romantic partners, family members), where needed. This may be warranted to assist these individuals in understanding the trauma and its impacts and upskilling them in supporting their loved one. Given the prevalence of “male rape myths” in the general population, engaging with loved ones may require dismantling harmful ideas about boys’ and men’s victimization. Consideration of cultural factors will be required, particularly factors that impact the way the boy or man is viewed and responded to within their networks and communities. • Health practitioners should consider opportunities for boys’ and men’s exposure to other men’s stories of sexual trauma, particularly stories underpinned by hope and resilience. Health practitioners may wish to provide boys and men with information about local peer support groups or services or online support groups and forums, particularly where face-to-face supports are limited. Referring to men who have discussed their experiences of ST publicly (e.g., memoirs, documentaries, interviews) may also be helpful strategies for combatting some of the shame, confusion, and isolation about these experiences.
Research	• Research investigating sexual abuse, assaults, and coercion requires greater inclusion of boys and men from a range of diverse backgrounds and identities, and consideration about how to directly target this population in research. • Further research is needed surrounding boys’ and men’s disclosure to social support (e.g., family members, friends, and partners), who are often likely to serve as initial disclosure sources. • Research is needed surrounding boys’ and men’s experiences of help-seeking from supports such as mental health practitioners for processing and healing from ST. This includes seeking understanding into effective ways of asking boys and men about their ST histories and creating safe spaces to share these experiences, responding to their disclosures in meaningful and supportive ways, useful therapeutic modalities and strategies for combatting shame, and minimizing disengagement throughout the help-seeking process. • Greater understanding is needed surrounding motivators and facilitators for disclosure in boys and men to inform opportunities for promoting disclosure, reporting, and help-seeking. • Increased quantitative research with greater methodological rigor is needed, with implementation of validated tools for assessing disclosure outcomes and possible predictors, mediators, and moderators of these outcomes. • Continued international investigation into boys’ and men’s experiences is required, with consideration of how experiences and needs are likely to be impacted by age, sexualities, legislation, service availability and resourcing, religious beliefs and practices, and ethnocultural identities.

This review highlights the need to promote widespread awareness about the prevalence and impacts of ST among boys and men via gender-inclusive educational and public health campaigns (Rice et al., 2022). Campaigns may benefit from representing higher-risk settings and groups, such as institutional and religious settings (Böhm et al., 2014; Jones & Pratt, 2008) and gender and sexual minority boys and men (Rothman et al., 2011). While further exploration surrounding effective health promotion strategies that will resonate with boys and men is certainly needed, these strategies will only be as effective as the systems and services in place to offer support to boys and men who have experienced ST. Recommending help-seeking in the absence of accessible avenues to obtain support risks further harm (Aguirre Velasco et al., 2020). It is essential that health promotion strategies occur alongside greater investment into support for ST-exposed boys and men.

Services that offer support to people exposed to ST would benefit from making it clear where boys and men are eligible to access their services, which may be communicated in writing or visual cues (e.g., including images of boys and men on websites, informational flyers, and advertisements). Findings also demonstrate the need to review and amend legislative language that fails to recognize assaults perpetrated against boys and men (Langdridge et al., 2023). Further, it is critical that professionals who are likely to encounter and offer support to ST-exposed boys and men are appropriately equipped. Development and implementation of trauma-informed, gender-sensitive training is recommended to support professionals to recognize indicators of ST in boys and men, inquire about ST, understand the varied impacts of ST in boys and men, and respond safely to their disclosures (Elkins et al., 2017; Teram et al.,, 2006). This may include upskilling mental health practitioners to address potential gendered impacts of ST, including impacts on masculine identities, and support boys and men to develop more adaptive definitions of masculinities following ST (Ralston, 2020; Widanaralalage et al., 2023). Effective care also requires understanding about various intersectional experiences of boys and men (Elkins et al., 2017), given cultural, community, and identity-related factors can play important roles in ST and disclosure experiences, including safety to disclose (Edwards et al., 2023).

Substantially more research with ST-exposed boys and men is needed across a range of identities and backgrounds (Widanaralalage et al., 2024). Greater methodological rigor is needed in quantitative research, with validated measurement tools, large samples of boys and men, and rigorous and clear reporting of statistical analyses. Given variables assessed as possible predictors of disclosure outcomes produced mixed and contradictory results, further investigation surrounding predictors, mediators, and moderators of disclosure outcomes is warranted to better understand factors driving non-disclosure and delayed disclosure. In quantitative and qualitative research, the literature is biased towards disclosure barriers, representing a deficit-based approach and preventing understanding of boys’ and men’s strength and resilience. Further investigation of motivators and facilitators of disclosure has the potential to inform health promotion initiatives. Finally, researchers must consider intersectional experiences and needs to ensure the representation of boys and men in all their diversity and avoid homogenizing this group (Elkins et al., 2017; Widanaralalage et al., 2024). Finally, we acknowledge that journeys towards healing and recovery do not end with disclosure. While more information about pathways to, and experiences of, initial disclosures is warranted, further information is also needed surrounding long-term discussions about ST and the recovery process in boys and men (Langdridge et al., 2023).

### Strengths and Limitations

A key strength of this review is the examination of factors impacting disclosure across ecological domains, facilitating an understanding of how disclosure is promoted and prevented across various social spheres. Consolidating evidence surrounding facilitating and motivating factors is timely, given these have been largely neglected to date (Alaggia et al., 2019). Crucially, this review seeks to address the minimal inclusion of boys and men in ST research and discourse (O'Gorman et al., 2023) and offers clinicians a repository of knowledge surrounding unique challenges faced by boys and men for seeking and accessing support following ST.

This review examined disclosure to a broad range of supports, including social supports, police, sexual assault services, medical practitioners, and mental health practitioners. While this limited understanding of disclosure within specific contexts, which often entail different motivations and challenges, this maximized the scope of the limited available evidence. Further, while we acknowledge that the dynamics and consequences of childhood versus adulthood ST can differ (Weiss, 2010), we opted to incorporate studies examining ST across the lifetime to maximize available evidence for review. While the four search databases may not have been exhaustive and non-peer-reviewed works were excluded, searching Google Scholar and reference lists of included articles sought to address this limitation. Finally, given resourcing constraints, only articles published in English were included, although evidence came from a broad range of countries across six continents.

## Conclusion

This review solidifies that boys and men experience gendered barriers to ST disclosure, complicating their access to needed support (PettyJohn et al., 2023). ST often evokes significant, lasting impacts on masculine self-concepts, which can play critical roles in preventing informal disclosure, formal reporting, and help-seeking. Boys’ and men’s experiences of ST are informed by perceived masculine norm violations and minimal public acknowledgment of their experiences (Widanaralalage et al., 2022). This is compounded by their limited prioritization in service provision, reflected by the dearth of tailored supports, treatment models, and training for professionals (O'Gorman et al., 2023). This risks limited effective recognition and response to gendered trauma responses in this group (Rice et al., 2022). There is a demonstrable need for further research in this domain, which may guide the development of gender-sensitive, trauma-informed approaches to care (Elkins et al., 2017; O'Gorman et al., 2023). Such work will be critical to developing appropriate supports and treatment models for ST-exposed boys and men, which has the potential to drastically improve health outcomes for this population.

## Supplemental Material

sj-docx-1-tva-10.1177_15248380251325210 – Supplemental material for Barriers and Facilitators for Sexual Trauma Disclosure in Boys and Men: A Systematic Review Supplemental material, sj-docx-1-tva-10.1177_15248380251325210 for Barriers and Facilitators for Sexual Trauma Disclosure in Boys and Men: A Systematic Review by Vita Pilkington, Sarah Bendall, Simon Rice, Michael Salter, Michael J. Wilson and Zac Seidler in Trauma, Violence, & Abuse

sj-docx-2-tva-10.1177_15248380251325210 – Supplemental material for Barriers and Facilitators for Sexual Trauma Disclosure in Boys and Men: A Systematic Review Supplemental material, sj-docx-2-tva-10.1177_15248380251325210 for Barriers and Facilitators for Sexual Trauma Disclosure in Boys and Men: A Systematic Review by Vita Pilkington, Sarah Bendall, Simon Rice, Michael Salter, Michael J. Wilson and Zac Seidler in Trauma, Violence, & Abuse

sj-docx-3-tva-10.1177_15248380251325210 – Supplemental material for Barriers and Facilitators for Sexual Trauma Disclosure in Boys and Men: A Systematic Review Supplemental material, sj-docx-3-tva-10.1177_15248380251325210 for Barriers and Facilitators for Sexual Trauma Disclosure in Boys and Men: A Systematic Review by Vita Pilkington, Sarah Bendall, Simon Rice, Michael Salter, Michael J. Wilson and Zac Seidler in Trauma, Violence, & Abuse

sj-docx-4-tva-10.1177_15248380251325210 – Supplemental material for Barriers and Facilitators for Sexual Trauma Disclosure in Boys and Men: A Systematic Review Supplemental material, sj-docx-4-tva-10.1177_15248380251325210 for Barriers and Facilitators for Sexual Trauma Disclosure in Boys and Men: A Systematic Review by Vita Pilkington, Sarah Bendall, Simon Rice, Michael Salter, Michael J. Wilson and Zac Seidler in Trauma, Violence, & Abuse

sj-docx-5-tva-10.1177_15248380251325210 – Supplemental material for Barriers and Facilitators for Sexual Trauma Disclosure in Boys and Men: A Systematic Review Supplemental material, sj-docx-5-tva-10.1177_15248380251325210 for Barriers and Facilitators for Sexual Trauma Disclosure in Boys and Men: A Systematic Review by Vita Pilkington, Sarah Bendall, Simon Rice, Michael Salter, Michael J. Wilson and Zac Seidler in Trauma, Violence, & Abuse

sj-docx-6-tva-10.1177_15248380251325210 – Supplemental material for Barriers and Facilitators for Sexual Trauma Disclosure in Boys and Men: A Systematic Review Supplemental material, sj-docx-6-tva-10.1177_15248380251325210 for Barriers and Facilitators for Sexual Trauma Disclosure in Boys and Men: A Systematic Review by Vita Pilkington, Sarah Bendall, Simon Rice, Michael Salter, Michael J. Wilson and Zac Seidler in Trauma, Violence, & Abuse

sj-docx-7-tva-10.1177_15248380251325210 – Supplemental material for Barriers and Facilitators for Sexual Trauma Disclosure in Boys and Men: A Systematic Review Supplemental material, sj-docx-7-tva-10.1177_15248380251325210 for Barriers and Facilitators for Sexual Trauma Disclosure in Boys and Men: A Systematic Review by Vita Pilkington, Sarah Bendall, Simon Rice, Michael Salter, Michael J. Wilson and Zac Seidler in Trauma, Violence, & Abuse

## Notes

1. Several articles used the same dataset but have been treated as separate studies, given they explored distinct outcomes and results (e.g., factors impacting disclosure to any source vs. factors impacting disclosure to mental health practitioners). Articles include: Easton (2013) and Easton et al. (2014); Foster (2017a) and Foster (2017b); Gagnier and Collin-Vezina (2016) and Gagnier et al., (2017); Sivagurunathan, Orchard, MacDermid, et al. (2019) and Sivagurunathan, Orchard, and Evans (2019); Jamel et al. (2008) and Jamel (2010).

2. Allows for multiple outcomes.

3. Authors assessed peer support using a single item that asked how often participants talked to their friends about “important things” ( “not at all,” “not very much,” “some,” “a lot”); responses were dichotomized and those reporting “some” and “a lot” were classified as having peer support.

4. Reference group was caring and non-overprotective parent relationships. This was compared to caring and over-protective relationships and to non-caring and not over-protective relationships.

## References

[bibr1-15248380251325210] Adejimi A. A. Sabageh O. A. Adedokun O. P. (2016). Experiences and disclosures of sexual assault among Nigerian undergraduates in a tertiary institution. Violence and Gender, 3(4), 208–215.

[bibr2-15248380251325210] Aguirre Velasco A. Cruz I. S. S. Billings J. Jimenez M. Rowe S . (2020). What are the barriers, facilitators and interventions targeting help-seeking behaviours for common mental health problems in adolescents? A systematic review. BMC Psychiatry, 20, 1–22. 32527236 10.1186/s12888-020-02659-0 PMC7291482

[bibr3-15248380251325210] Alaggia R. (2005). Disclosing the trauma of child sexual abuse: A gender analysis. Journal of Loss and Trauma, 10(5), 453–470.

[bibr4-15248380251325210] Alaggia R. Collin-Vézina D. Lateef R. (2019). Facilitators and barriers to child sexual abuse (CSA) disclosures: A research update (2000–2016). Trauma, Violence, & Abuse, 20(2), 260–283. 10.1177/1524838017697312 PMC6429637 29333973

[bibr5-15248380251325210] Alam S. O’Halloran S. Fowke A. (2024). What are the barriers to mental health support for racially-minoritised people within the UK? A systematic review and thematic synthesis. The Cognitive Behaviour Therapist, 17, e10.

[bibr6-15248380251325210] Aspin C. Reynolds P. Lehavot K. Taiapa J. (2009). An investigation of the phenomenon of non-consensual sex among Maori men who have sex with men. Culture, Health & Sexuality, 11(1), 35–49. 10.1080/13691050802483711 19234949

[bibr7-15248380251325210] Attrash-Najjar A. Cohen N. Glucklich T. Katz C. (2023). “I was the only one talking about the abuse”: Experiences and perceptions of survivors who underwent child sexual abuse as boys. Child Abuse & Neglect, 140, 106144. 36965436 10.1016/j.chiabu.2023.106144

[bibr8-15248380251325210] Böhm B. Zollner H. Fegert J. M. Liebhardt H. (2014). Child sexual abuse in the context of the Roman Catholic Church: A review of literature from 1981–2013. Journal of Child Sexual Abuse, 23(6), 635–656. 24911986 10.1080/10538712.2014.929607

[bibr9-15248380251325210] Boudreau C. L. Kress H. Rochat R. W. Yount K. M. (2018). Correlates of disclosure of sexual violence among Kenyan youth. Child Abuse & Neglect, 79, 164–172. 29459242 10.1016/j.chiabu.2018.01.025 PMC6091645

[bibr10-15248380251325210] Braun V. Clarke V. (2012). Thematic analysis: American Psychological Association.

[bibr11-15248380251325210] Braun V. Schmidt J. Gavey N. Fenaughty J. (2009). Sexual coercion among gay and bisexual men in Aotearoa/New Zealand. Journal of Homosexuality, 56(3), 336–360. 19319741 10.1080/00918360902728764

[bibr12-15248380251325210] Broban A. Van den Bergh R. Russell W. Benedetti G. Caluwaerts S. Owiti P. Reid A. De Plecker E. (2020). Assault and care characteristics of victims of sexual violence in eleven Médecins Sans Frontières programs in Africa. What about men and boys? PLoS One, 15(8), e0237060. 32750062 10.1371/journal.pone.0237060 PMC7402504

[bibr13-15248380251325210] Bronfenbrenner U. (1979). The ecology of human development. Harvard University Press.

[bibr14-15248380251325210] Canan S. N. Haus K. R. Wiersma-Mosley J. D. Jozkowski K. N. (2023). Familial support and disclosure: A two-sample study of LGBT sexual assault. LGBTQ+ Family: An Interdisciplinary Journal, 19(1), 54–69.

[bibr15-15248380251325210] Cashmore J. Taylor A. Parkinson P. (2017). The characteristics of reports to the police of child sexual abuse and the likelihood of cases proceeding to prosecution after delays in reporting. Child Abuse & Neglect, 74, 49–61. 28803002 10.1016/j.chiabu.2017.07.006

[bibr16-15248380251325210] CASP C. A. S. P . (2018). Critical Appraisal Checklists. https://casp-uk.net/casp-tools-checklists/

[bibr17-15248380251325210] Catton A. K. Dorahy M. J. (2024). The Sexual Encounters Questionnaire: A gender-inclusive survey of sexual victimization across the lifespan. Psychological Trauma: Theory, Research, Practice, and Policy. 10.1037/tra0001695 38546598

[bibr18-15248380251325210] Cheng C. (1999). Marginalized masculinities and hegemonic masculinity: An introduction. The Journal of Men’s Studies, 7(3), 295–315.

[bibr19-15248380251325210] Christian M. Safari O. Ramazani P. Burnham G. Glass N. (2011). Sexual and gender based violence against men in the Democratic Republic of Congo: effects on survivors, their families and the community. Medicine, Conflict and Survival, 27(4), 227–246. 22416570 10.1080/13623699.2011.645144

[bibr20-15248380251325210] Chynoweth S. K. Buscher D. Martin S. Zwi A. B. (2020). A social ecological approach to understanding service utilization barriers among male survivors of sexual violence in three refugee settings: a qualitative exploratory study. Conflict and Health, 14(1), 1–13. 32670397 10.1186/s13031-020-00288-8 PMC7346522

[bibr21-15248380251325210] Collin-Vézina D. De La Sablonnière-Griffin M. Palmer A. M. Milne L. (2015). A preliminary mapping of individual, relational, and social factors that impede disclosure of childhood sexual abuse. Child Abuse & Neglect, 43, 123–134. 25846196 10.1016/j.chiabu.2015.03.010

[bibr22-15248380251325210] Collin-Vézina D. De La Sablonnière-Griffin M. Sivagurunathan M. Lateef R. Alaggia R. McElvaney R. Simpson M. (2021). “How many times did I not want to live a life because of him”: the complex connections between child sexual abuse, disclosure, and self-injurious thoughts and behaviors. Borderline Personality Disorder and Emotion Dysregulation, 8, 1–13. 33397506 10.1186/s40479-020-00142-6 PMC7783974

[bibr23-15248380251325210] Corboz J. Pasquero L. Hogg C. L. Rasheed A. (2023). Enhancing a survivor-centred approach to healthcare provision in Afghanistan: Understanding and addressing the barriers faced by male victims/survivors of sexual violence. Child Abuse & Neglect, 142, 105854. 36031438 10.1016/j.chiabu.2022.105854

[bibr24-15248380251325210] Coxell A. W. King B. Mezey G. C. Kell P. (2000). Sexual molestation of men: Interviews with 224 men attending a genitourinary medicine service. International Journal of STD & AIDS, 11(9), 574–578. 10997498 10.1258/0956462001916542

[bibr25-15248380251325210] Craner J. R. Martinson A. A. Sigmon S. T. McGillicuddy M. L. (2015). Prevalence of sexual trauma history using behaviorally specific methods of assessment in first year college students. Journal of Child Sexual Abuse, 24(5), 484–505. 26090864 10.1080/10538712.2015.1026014

[bibr26-15248380251325210] Depraetere J. Vandeviver C. Beken T. V. Keygnaert I. (2020). Big boys don’t cry: A critical interpretive synthesis of male sexual victimization. Trauma, Violence, & Abuse, 21(5), 991–1010. 10.1177/1524838018816979 PMC7444022 30554559

[bibr27-15248380251325210] Donne M. D. DeLuca J. Pleskach P. Bromson C. Mosley M. P. Perez E. T. Mathews S. G. Stephenson E. Frye V. (2018). Barriers to and facilitators of help-seeking behavior among men who experience sexual violence. American Journal of Men's Health, 12(2), 189–201. 10.1177/1557988317740665 PMC5818122 29161934

[bibr28-15248380251325210] Easton S. D. (2013). Disclosure of child sexual abuse among adult male survivors. Clinical Social Work Journal, 41(4), 344–355.

[bibr29-15248380251325210] Easton S. D. Leone-Sheehan D. M. Sophis E. J. Willis D. G. (2015). “From that moment on my life changed”: Turning points in the healing process for men recovering from child sexual abuse. Journal of Child Sexual Abuse, 24(2), 152–173. 25747418 10.1080/10538712.2015.997413

[bibr30-15248380251325210] Easton S. D. Renner L. M. O’Leary P. (2013). Suicide attempts among men with histories of child sexual abuse: Examining abuse severity, mental health, and masculine norms. Child Abuse & Neglect, 37(6), 380–387. 23313078 10.1016/j.chiabu.2012.11.007

[bibr31-15248380251325210] Easton S. D. Saltzman L. Y. Willis D. G. (2014). “Would you tell under circumstances like that?”: Barriers to disclosure of child sexual abuse for men. Psychology of Men & Masculinity, 15(4), 460.

[bibr32-15248380251325210] Edwards K. M. Mauer V. A. Huff M. Farquhar-Leicester A. Sutton T. E. Ullman S. E. (2023). Disclosure of sexual assault among sexual and gender minorities: A systematic literature review. Trauma, Violence, & Abuse, 24(3), 1608–1623. 10.1177/15248380211073842 35403506

[bibr33-15248380251325210] Eisenberg M. E. Lust K. Mathiason M. A. Porta C. M. (2021). Sexual assault, sexual orientation, and reporting among college students. Journal of Interpersonal Violence, 36(1-2), 62–82. 29294876 10.1177/0886260517726414

[bibr34-15248380251325210] Elder W. B. Domino J. L. Mata-Galán E. L. Kilmartin C. (2017). Masculinity as an avoidance symptom of posttraumatic stress. Psychology of Men & Masculinity, 18(3), 198.

[bibr35-15248380251325210] Elder W. B. Domino J. L. Rentz T. O. Mata-Galán E. L. (2017). Conceptual model of male military sexual trauma. Psychological Trauma: Theory, Research, Practice, and Policy, 9(S1), 59. 27669163 10.1037/tra0000194

[bibr36-15248380251325210] Elkins J. Crawford K. Briggs H. E. (2017). Male survivors of sexual abuse: Becoming gender-sensitive and trauma-informed. Advances in Social Work, 18(1), 116–130.

[bibr37-15248380251325210] Elliott D. M. Mok D. S. Briere J. (2004). Adult sexual assault: Prevalence, symptomatology, and sex differences in the general population. Journal of Traumatic Stress, 17(3), 203–211. 15253092 10.1023/B:JOTS.0000029263.11104.23

[bibr38-15248380251325210] Elliott S. A. Goodman K. L. Bardwell E. S. Mullin T. M. (2022). Reactions to the disclosure of intrafamilial childhood sexual abuse: Findings from the National Sexual Assault Online Hotline. Child Abuse & Neglect, 127, 105567. 35278820 10.1016/j.chiabu.2022.105567

[bibr39-15248380251325210] Forde C. Duvvury N. (2017). Sexual violence, masculinity, and the journey of recovery. Psychology of Men & Masculinity, 18(4), 301.

[bibr40-15248380251325210] Foster J. M. (2017a). It happened to me: A qualitative analysis of boys’ narratives about child sexual abuse. Journal of Child Sexual Abuse, 26(7), 853–873. 28857688 10.1080/10538712.2017.1360426

[bibr41-15248380251325210] Foster J. M. (2017b). The fears and futures of boy victims of sexual abuse: An analysis of narratives. Journal of Child Sexual Abuse, 26(6), 710–730. 28836930 10.1080/10538712.2017.1339223

[bibr42-15248380251325210] Frías S. M. Erviti J. (2014). Gendered experiences of sexual abuse of teenagers and children in Mexico. Child Abuse & Neglect, 38(4), 776-787. 24445000 10.1016/j.chiabu.2013.12.001

[bibr43-15248380251325210] Gagnier C. Collin-Vézina D. (2016). The disclosure experiences of male child sexual abuse survivors. Journal of Child Sexual Abuse, 25(2), 221–241. 26934546 10.1080/10538712.2016.1124308

[bibr44-15248380251325210] Gagnier C. Collin-Vézina D. La Sablonnière-Griffin M. D. (2017). The journey of obtaining services: The realities of male survivors of childhood sexual abuse. Journal of Child & Adolescent Trauma, 10, 129–137.

[bibr45-15248380251325210] Gauthier-Duchesne A. Fernet M. Hébert M. Guyon R. Tardif M. Godbout N. (2024). The externalization of suffering among male survivors of child sexual abuse:“A deeply buried rage that must come out”. Psychology of Men & Masculinities, 25(2), 142.

[bibr46-15248380251325210] Gill A. K. Begum H. (2023). ‘They wouldn’t believe me’: Giving a voice to British south Asian male survivors of child sexual abuse. The British Journal of Criminology, 63(5), 1146–1164.

[bibr47-15248380251325210] Gilligan P. Akhtar S. (2006). Cultural barriers to the disclosure of child sexual abuse in Asian communities: Listening to what women say. British Journal of Social Work, 36(8), 1361–1377.

[bibr48-15248380251325210] Gruenfeld E. Willis D. G. Easton S. D. (2017). “A very steep climb”: therapists’ perspectives on barriers to disclosure of child sexual abuse experiences for men. Journal of Child Sexual Abuse, 26(6), 731–751. 28657500 10.1080/10538712.2017.1332704

[bibr49-15248380251325210] Guerra C. Arredondo V. Saavedra C. Pinto-Cortez C. Benguria A. Orrego A. (2021). Gender differences in the disclosure of sexual abuse in Chilean adolescents. Child Abuse Review, 30(3), 210–225.

[bibr50-15248380251325210] Gundlapalli A. V. Jones A. L. Redd A. Divita G. Brignone E. Pettey W. B. Carter M. E. Samore M. H. Blais R. K. Fargo J. D. (2019). Combining Natural Language Processing of electronic medical notes with administrative data to determine racial/ethnic differences in the disclosure and documentation of military sexual trauma in veterans. Medical Care, 57, S149–S156. 31095054 10.1097/MLR.0000000000001031

[bibr51-15248380251325210] Hahn C. K. Turchik J. Kimerling R. (2021). A latent class analysis of mental health beliefs related to military sexual trauma. Journal of Traumatic Stress, 34(2), 394–404. 32969098 10.1002/jts.22585 PMC7985046

[bibr52-15248380251325210] Hanson R. F. Kievit L. W. Saunders B. E. Smith D. W. Kilpatrick D. G. Resnick H. S. Ruggiero K. J. (2003). Correlates of adolescent reports of sexual assault: Findings from the National Survey of Adolescents. Child Maltreatment, 8(4), 261–272. 14604174 10.1177/1077559503257087

[bibr53-15248380251325210] Heron R. L. Eisma M. C. (2021). Barriers and facilitators of disclosing domestic violence to the healthcare service: a systematic review of qualitative research. Health & Social Care in the Community, 29(3), 612–630. 33440034 10.1111/hsc.13282 PMC8248429

[bibr54-15248380251325210] Hershkowitz I. Horowitz D. Lamb M. E. (2005). Trends in children's disclosure of abuse in Israel: A national study. Child Abuse & Neglect, 29(11), 1203–1214. 16260036 10.1016/j.chiabu.2005.04.008

[bibr55-15248380251325210] Hietamäki J. Husso M. Arponen T. Lahtinen H.-M. (2024). Differences between girls and boys in the disclosure of sexual violence. Journal of Interpersonal Violence, 39(11–12), 2629–2654. 38254297 10.1177/08862605231221283 PMC11071602

[bibr56-15248380251325210] Hine B. Bates E. A. Wallace S. (2022). “I have guys call me and say ‘I can’t be the victim of domestic abuse’”: Exploring the experiences of telephone support providers for male victims of domestic violence and abuse. Journal of Interpersonal Violence, 37(7–8), NP5594–NP5625. 10.1177/0886260520944551 PMC8980445 32727270

[bibr57-15248380251325210] Hine B. A. Murphy A. D. Churchyard J. S. (2021). Development and validation of the male rape myth acceptance scale (MRMAS). Heliyon, 7(6), e07421. 10.1016/j.heliyon.2021.e07421 PMC8258646 34307931

[bibr58-15248380251325210] Hlavka H. R. (2017). Speaking of stigma and the silence of shame: Young men and sexual victimization. Men and Masculinities, 20(4), 482–505.

[bibr59-15248380251325210] Holland K. J. Cipriano A. E. (2021). Does a report= support? A qualitative analysis of college sexual assault survivors’ Title IX Office knowledge, perceptions, and experiences. Analyses of Social Issues and Public Policy, 21(1), 1054–1081.

[bibr60-15248380251325210] Hong Q. N. Pluye P. Fabregues P. Bartlett G. Boardman F. Cargo M. Dagenais P. Gagnon M. P. Griffiths F. Nicolau B. O'Cathain A. Rousseau M. C. Vedel I. (2018). Mixed-methods appraisal tool (MMAT) version 2018: User guide. http://mixedmethodsappraisaltoolpublic.pbworks.com/w/file/fetch/127916259/MMAT_2018_criteria-manual_2018-08-01_ENG.pdf 10.1016/j.jclinepi.2019.03.008 30905698

[bibr61-15248380251325210] Hunter S. V. (2011). Disclosure of child sexual abuse as a life-long process: Implications for health professionals. Australian and New Zealand Journal of Family Therapy, 32(2), 159–172.

[bibr62-15248380251325210] Jackson M. A. Valentine S. E. Woodward E. N. Pantalone D. W. (2017). Secondary victimization of sexual minority men following disclosure of sexual assault: “Victimizing me all over again. . .”. Sexuality Research and Social Policy, 14, 275–288.

[bibr63-15248380251325210] Jacoby S. F. Rich J. A. Webster J. L. Richmond T. S. (2020). ‘Sharing things with people that I don’t even know’: help-seeking for psychological symptoms in injured black men in Philadelphia. Ethnicity & Health, 25(6), 777–795. 29607675 10.1080/13557858.2018.1455811 PMC6167172

[bibr64-15248380251325210] Jamel J. (2010). Researching the provision of service to rape victims by specially trained police officers: The influence of gender–an exploratory study. New Criminal Law Review, 13(4), 688–709.

[bibr65-15248380251325210] Jamel J. Bull R. Sheridan L. (2008). An investigation of the specialist police service provided to male rape survivors. International Journal of Police Science & Management, 10(4), 486-508.

[bibr66-15248380251325210] Javaid A. (2018). Male rape, masculinities, and sexualities. International Journal of Law, Crime and Justice, 52, 199–210.

[bibr67-15248380251325210] JBI. (2020). JBI critical appraisal checklist for analytical cross-sectional studies. https://jbi.global/critical-appraisal-tools

[bibr68-15248380251325210] Jones T. R. Pratt T. C. (2008). The prevalence of sexual violence in prison: the state of the knowledge base and implications for evidence-based correctional policy making. International journal of Offender Therapy and Comparative Criminology, 52(3), 280–295. 17893207 10.1177/0306624X07307631

[bibr69-15248380251325210] Kemshall H. Moulden H. M. (2017). Communicating about child sexual abuse with the public: Learning the lessons from public awareness campaigns. Journal of Sexual Aggression, 23(2), 124–138.

[bibr70-15248380251325210] Kennedy A. C. Prock K. A. (2018). “I still feel like I am not normal”: A review of the role of stigma and stigmatization among female survivors of child sexual abuse, sexual assault, and intimate partner violence. Trauma, Violence, & Abuse, 19(5), 512–527. 10.1177/1524838016673601 27803311

[bibr71-15248380251325210] Kia-Keating M. Grossman F. K. Sorsoli L. Epstein M. (2005). Containing and Resisting Masculinity: Narratives of Renegotiation Among Resilient Male Survivors of Childhood Sexual Abuse. Psychology of Men & Masculinity, 6(3), 169.

[bibr72-15248380251325210] Kim E. Y. Y. Nelson L. E. Pereira T. L.-B. Shorey S. (2023). Barriers to and facilitators of help-seeking among men who are victims of domestic violence: a mixed-studies systematic review. Trauma, Violence, & Abuse, 15248380231209435. 10.1177/15248380231209435 37970823

[bibr73-15248380251325210] Kwon I. Lee D.-O. Kim E. Kim H.-Y. (2007). Sexual violence among men in the military in South Korea. Journal of Interpersonal Violence, 22(8), 1024–1042. 17709808 10.1177/0886260507302998

[bibr74-15248380251325210] Langdridge D. Flowers P. Carney D. (2023). Male survivors' experience of sexual assault and support: A scoping review. Aggression and Violent Behavior, 101838.

[bibr75-15248380251325210] Latiff M. A. Fang L. Goh D. A. Tan L. J. (2024). A systematic review of factors associated with disclosure of child sexual abuse. Child Abuse & Neglect, 147, 106564. 38056036 10.1016/j.chiabu.2023.106564

[bibr76-15248380251325210] Lehrer J. A. Lehrer E. L. Koss M. P. (2013). Unwanted sexual experiences in young men: Evidence from a survey of university students in Chile. Archives of Sexual Behavior, 42, 213–223. 22971801 10.1007/s10508-012-0004-x PMC4475681

[bibr77-15248380251325210] Lemaigre C. Taylor E. P. Gittoes C. (2017). Barriers and facilitators to disclosing sexual abuse in childhood and adolescence: A systematic review. Child Abuse & Neglect, 70, 39–52. 28551460 10.1016/j.chiabu.2017.05.009

[bibr78-15248380251325210] Manor-Binyamini I. Schreiber-Divon M. (2023). Exposing the secret: listening to Bedouin men who have experienced sexual violence. Journal of Interpersonal Violence, 38(3–4), 3468–3488. 35658742 10.1177/08862605221107054

[bibr79-15248380251325210] Masho S. W. Alvanzo A. (2010). Help-seeking behaviors of men sexual assault survivors. American Journal of Men's Health, 4(3), 237–242. 10.1177/1557988309336365 19706673

[bibr80-15248380251325210] Mathews B. Bromfield L. Walsh K. Cheng Q. Norman R. E. (2017). Reports of child sexual abuse of boys and girls: Longitudinal trends over a 20-year period in Victoria, Australia. Child Abuse & Neglect, 66, 9–22. 28222908 10.1016/j.chiabu.2017.01.025

[bibr81-15248380251325210] McElvaney R. Greene S. Hogan D. (2012). Containing the secret of child sexual abuse. Journal of Interpersonal Violence, 27(6), 1155–1175. 22203619 10.1177/0886260511424503

[bibr82-15248380251325210] Mgolozeli S. E. Duma S. E. (2020). “They all laughed and asked me if I enjoyed having sex with those guys”: Exploring men’s lived experiences when reporting rape to police in South Africa. PLoS One, 15(8), e0235044. 32822366 10.1371/journal.pone.0235044 PMC7444553

[bibr83-15248380251325210] Millegan J. Wang L. LeardMann C. A. Miletich D. Street A. E. (2016). Sexual trauma and adverse health and occupational outcomes among men serving in the US military. Journal of Traumatic Stress, 29(2), 132–140. 27077493 10.1002/jts.22081

[bibr84-15248380251325210] Moher D. Liberati A. Tetzlaff J. Altman D. G. Group P. (2009). Preferred reporting items for systematic reviews and meta-analyses: the PRISMA statement. Annals of Internal Medicine, 151(4), 264–269. 19622511 10.7326/0003-4819-151-4-200908180-00135

[bibr85-15248380251325210] Moody G. Cannings-John R. Hood K. Kemp A. Robling M. (2018). Establishing the international prevalence of self-reported child maltreatment: a systematic review by maltreatment type and gender. BMC Public Health, 18(1), 1–15. 10.1186/s12889-018-6044-y PMC6180456 30305071

[bibr86-15248380251325210] Morrison S. E. Bruce C. Wilson S. (2018). Children’s disclosure of sexual abuse: A systematic review of qualitative research exploring barriers and facilitators. Journal of Child Sexual Abuse, 27(2), 176–194. 29488844 10.1080/10538712.2018.1425943

[bibr87-15248380251325210] Nicholas A. Krysinska K. King K. E. (2022). A rapid review to determine the suicide risk and risk factors of men who are survivors of sexual assault. Psychiatry Research, 317, 114847. 36126347 10.1016/j.psychres.2022.114847

[bibr88-15248380251325210] Nofziger S. Stein R. E. (2006). To tell or not to tell: Lifestyle impacts on whether adolescents tell about violent victimization. Violence and Victims, 21(3), 371–382. 16761860 10.1891/vivi.21.3.371

[bibr89-15248380251325210] O'Gorman K. Pilkington V. Seidler Z. Oliffe J. L. Peters W. Bendall S. Rice S. M . (2023). Childhood sexual abuse in boys and men: The case for gender-sensitive interventions. Psychological Trauma: Theory, Research, Practice, and Policy, 16(S1), S181. 10.1037/tra0001520 37326539

[bibr90-15248380251325210] Okur P. van der Knaap L. M. Bogaerts S. (2020). A quantitative study on gender differences in disclosing child sexual abuse and reasons for nondisclosure. Journal of Interpersonal Violence, 35(23–24), 5255–5275. 29294841 10.1177/0886260517720732

[bibr91-15248380251325210] Orchowski L. M. Gidycz C. A. (2015). Psychological consequences associated with positive and negative responses to disclosure of sexual assault among college women: A prospective study. Violence against Women, 21(7), 803–823. 25926138 10.1177/1077801215584068 PMC4632843

[bibr92-15248380251325210] Oueis J. McKie R. M. Reissing E. D. (2024). A qualitative account of coping following non-consensual sexual experiences among gay, bisexual, and other men who have sex with men. The Journal of Sex Research, 61(3), 414–426. 37310380 10.1080/00224499.2023.2220694

[bibr93-15248380251325210] Pacheco E. L. M. Buenaventura A. E. Miles G. M. (2023). “She was willing to send me there”: Intrafamilial child sexual abuse, exploitation and trafficking of boys. Child Abuse & Neglect, 142, 105849. 36369043 10.1016/j.chiabu.2022.105849

[bibr94-15248380251325210] Page M. J. McKenzie J. E. Bossuyt P. M. Boutron I. Hoffmann T. C. Mulrow C. D. Shamseer L. Tetzlaff J. M. Moher D. (2021). Updating guidance for reporting systematic reviews: development of the PRISMA 2020 statement. Journal of Clinical Epidemiology, 134, 103–112. 33577987 10.1016/j.jclinepi.2021.02.003

[bibr95-15248380251325210] Patterson T. Campbell A. La Rooy D. Hobbs L. Clearwater K. Rapsey C. (2023). Impact, ramifications and taking back control: A qualitative study of male survivors of childhood sexual abuse. Journal of Interpersonal Violence, 38(1–2), 1868–1892. 10.1177/08862605221094629 35487882

[bibr96-15248380251325210] Peterson Z. D. Voller E. K. Polusny M. A. Murdoch M. (2011). Prevalence and consequences of adult sexual assault of men: Review of empirical findings and state of the literature. Clinical Psychology Review, 31(1), 1–24. 21130933 10.1016/j.cpr.2010.08.006

[bibr97-15248380251325210] Petersson C. C. Plantin L. (2019). Breaking with norms of masculinity: Men making sense of their experience of sexual assault. Clinical Social Work Journal, 47(4), 372–383.

[bibr98-15248380251325210] PettyJohn M. E. Reid T. A. Cary K. M. Greer K. M. Nason J. A. Agundez J. C. Carin G. McCauley H. L. (2023). “I don’t know what the hell you’d call it”: A qualitative thematic synthesis of men’s experiences with sexual violence in adulthood as contextualized by hegemonic masculinity. Psychology of Men & Masculinities, 24(4), 272–290.

[bibr99-15248380251325210] Postmus J. L. Hoge G. L. Davis R. Johnson L. Koechlein E. Winter S. (2015). Examining gender based violence and abuse among Liberian school students in four counties: An exploratory study. Child Abuse & Neglect, 44, 76–86. 25529859 10.1016/j.chiabu.2014.11.012

[bibr100-15248380251325210] Priebe G. Svedin C. G. (2008). Child sexual abuse is largely hidden from the adult society: An epidemiological study of adolescents’ disclosures. Child Abuse & Neglect, 32(12), 1095–1108. 19038448 10.1016/j.chiabu.2008.04.001

[bibr101-15248380251325210] Priebe G. Svedin C. G. (2012). Online or off-line victimisation and psychological well-being: A comparison of sexual-minority and heterosexual youth. European Child & Adolescent Psychiatry, 21, 569–582. 22772657 10.1007/s00787-012-0294-5

[bibr102-15248380251325210] Ralston K. M. (2020). “If i was a ‘real man’”: The role of gender stereotypes in the recovery process for men who experience sexual victimization. The Journal of Men’s Studies, 28(2), 127–148.

[bibr103-15248380251325210] Rapsey C. Campbell A. Clearwater K. Patterson T. (2020). Listening to the therapeutic needs of male survivors of childhood sexual abuse. Journal of Interpersonal Violence, 35(9–10), 2033–2054. 10.1177/0886260517701453 29294699

[bibr104-15248380251325210] Reeves A. Stewart S. (2017). Healing the spirit: Exploring sexualized trauma and recovery among Indigenous men in Toronto. American Indian and Alaska Native Mental Health Research, 24(1), 30–60. 28562836 10.5820/aian.2401.2017.30

[bibr105-15248380251325210] Rice S. M. Easton S. D. Seidler Z. E. Oliffe J. L. (2022). Sexual abuse and mental ill health in boys and men: what we do and don't know. BJPsych Open, 8(4), e110. 10.1192/bjo.2022.508 PMC9230611 35678473

[bibr106-15248380251325210] Roberts S. (2020). Untold stories: Male child sexual abusers’ accounts of telling and not telling about sexual abuse experienced in childhood. Journal of Child Sexual Abuse, 29(8), 965–983. 33185510 10.1080/10538712.2020.1841351

[bibr107-15248380251325210] Romano E. Moorman J. Ressel M. Lyons J. (2019). Men with childhood sexual abuse histories: Disclosure experiences and links with mental health. Child Abuse & Neglect, 89, 212–224. 30710773 10.1016/j.chiabu.2018.12.010

[bibr108-15248380251325210] Rothman E. F. Exner D. Baughman A. L. (2011). The prevalence of sexual assault against people who identify as gay, lesbian, or bisexual in the United States: A systematic review. Trauma, Violence, & Abuse, 12(2), 55–66. 10.1177/1524838010390707 PMC3118668 21247983

[bibr109-15248380251325210] Segal L. Gnanamanickam E. S. (2024). The Australian Child Maltreatment Study: National prevalence and associated health outcomes of child abuse and neglect. Medical Journal of Australia, 220(5), 275. 10.5694/mja2.52230 38375581

[bibr110-15248380251325210] Seidler Z. E. Dawes A. J. Rice S. M. Oliffe J. L. Dhillon H. M. (2016). The role of masculinity in men's help-seeking for depression: a systematic review. Clinical Psychology Review, 49, 106–118. 27664823 10.1016/j.cpr.2016.09.002

[bibr111-15248380251325210] Sharma A. (2022). Disclosure of child sexual abuse: Experiences of men survivors in India. The British Journal of Social Work, 52(8), 4588–4605.

[bibr112-15248380251325210] Sivagurunathan M. Orchard T. Evans M. (2019). Barriers to utilization of mental health services amongst male child sexual abuse survivors: Service providers’ perspective. Journal of Child Sexual Abuse, 28(7), 819–839. 31184546 10.1080/10538712.2019.1610823

[bibr113-15248380251325210] Sivagurunathan M. Orchard T. MacDermid J. C. Evans M. (2019). Barriers and facilitators affecting self-disclosure among male survivors of child sexual abuse: The service providers’ perspective. Child Abuse & Neglect, 88, 455–465. 30219431 10.1016/j.chiabu.2018.08.015

[bibr114-15248380251325210] Smith S. G. Zhang X. Basile K. C. Merrick M. T. Wang J. Kresnow M.-J. Chen J. (2018). The national intimate partner and sexual violence survey: 2015 data brief–updated release. CDC. https://stacks.cdc.gov/view/cdc/60893 10.1016/j.amepre.2018.01.014 PMC6007810 29449134

[bibr115-15248380251325210] Sorsoli L. Kia-Keating M. Grossman F. K. (2008). “I keep that hush-hush”: Male survivors of sexual abuse and the challenges of disclosure. Journal of Counseling Psychology, 55(3), 333.

[bibr116-15248380251325210] Stemple L. Meyer I. H. (2014). The sexual victimization of men in America: New data challenge old assumptions. American journal of public health, 104(6), e19–e26. 10.2105/AJPH.2014.301946 PMC4062022 24825225

[bibr117-15248380251325210] Stoltenborgh M. Van Ijzendoorn M. H. Euser E. M. Bakermans-Kranenburg M. J. (2011). A global perspective on child sexual abuse: Meta-analysis of prevalence around the world. Child Maltreatment, 16(2), 79–101. 21511741 10.1177/1077559511403920

[bibr118-15248380251325210] Stoner J. E. Cramer R. J. (2019). Sexual violence victimization among college females: A systematic review of rates, barriers, and facilitators of health service utilization on campus. Trauma, Violence, & Abuse, 20(4), 520–533. 10.1177/1524838017721245 29333989

[bibr119-15248380251325210] Struckman-Johnson C. Struckman-Johnson D. (1992). Acceptance of male rape myths among college men and women. Sex Roles, 27, 85–100.

[bibr120-15248380251325210] Tener D. Murphy S. B. (2015). Adult disclosure of child sexual abuse: A literature review. Trauma, Violence, & Abuse, 16(4), 391–400. 10.1177/1524838014537906 24903400

[bibr121-15248380251325210] Teram E. Stalker C. Hovey A. Schachter C. Lasiuk G. (2006). Towards malecentric communication: Sensitizing health professionals to the realities of male childhood sexual abuse survivors. Issues in Mental Health Nursing, 27(5), 499–517. 16613801 10.1080/01612840600599994

[bibr122-15248380251325210] Tillman S. Bryant-Davis T. Smith K. Marks A. (2010). Shattering silence: Exploring barriers to disclosure for African American sexual assault survivors. Trauma, Violence, & Abuse, 11(2), 59–70. 10.1177/1524838010363717 20430798

[bibr123-15248380251325210] Turchik J. A. Edwards K. M. (2012). Myths about male rape: A literature review. Psychology of Men & Masculinity, 13(2), 211.

[bibr124-15248380251325210] Turchik J. A. McLean C. Rafie S. Hoyt T. Rosen C. S. Kimerling R. (2013). Perceived barriers to care and provider gender preferences among veteran men who have experienced military sexual trauma: a qualitative analysis. Psychological Services, 10(2), 213. 22984877 10.1037/a0029959

[bibr125-15248380251325210] Velloza J. Davies L. Ensminger A. Theofelus F. M. Andjamba H. Kamuingona R. Nakuta J. Uiras W. Massetti G. Coomer R. Wolkon A. Forster N. Coomer R. (2022). Disclosure and help-seeking behaviors related to sexual and physical violence in childhood and adolescence: Results from the Namibia Violence Against Children and Youth Survey. Child Abuse & Neglect, 128, 105624. 35381545 10.1016/j.chiabu.2022.105624 PMC9119951

[bibr126-15248380251325210] Walfield S. M. McCormack P. Clarke K. (2024). Male victims of sexual violence and factors associated with reporting to law enforcement in the United States. The Journal of Men’s Studies, 32(3), 577–594.

[bibr127-15248380251325210] Weare S. Hulley J. Craig D. (2024). ‘Nobody believes you if you’re a bloke’: Barriers to disclosure and help-seeking for male forced-to-penetrate victims/survivors. International Review of Victimology, 30(3), 596–611. 10.1177/02697580241238768

[bibr128-15248380251325210] Weiss K. G. (2010). Male sexual victimization: Examining men’s experiences of rape and sexual assault. Men and Masculinities, 12(3), 275–298.

[bibr129-15248380251325210] Widanaralalage B. K. Hine B. A. Murphy A. D. Murji K. (2022). “I didn’t feel I was a victim”: A phenomenological analysis of the experiences of male-on-male survivors of rape and sexual abuse. Victims & Offenders, 17(8), 1147–1172.

[bibr130-15248380251325210] Widanaralalage B. K. Hine B. A. Murphy A. D. Murji K. (2023). A qualitative investigation of service providers’ experiences supporting raped and sexually abused men. Springer. 10.1891/VV-2022-0084 36717192

[bibr131-15248380251325210] Widanaralalage B. K. Jennings S. Dando C. Mackenzie J.-M. (2024). Prevalence, disclosure, and help seeking in Black and Asian male survivors of sexual violence in the United Kingdom: a rapid review. Trauma, Violence, & Abuse, 25(4), 3299–3314. 10.1177/15248380241246217 PMC11370200 38629644

[bibr132-15248380251325210] Young S. M. Pruett J. A. Colvin M. L. (2018). Comparing help-seeking behavior of male and female survivors of sexual assault: A content analysis of a hotline. Sexual Abuse, 30(4), 454–474. 27864570 10.1177/1079063216677785

[bibr133-15248380251325210] Zalcberg S. (2017). The place of culture and religion in patterns of disclosure and reporting sexual abuse of males: A case study of ultra orthodox male victims. Journal of Child Sexual Abuse, 26(5), 590–607. 28696908 10.1080/10538712.2017.1316335

[bibr134-15248380251325210] Zinzow H. M. Littleton H. Muscari E. Sall K. (2022). Barriers to formal help-seeking following sexual violence: Review from within an ecological systems framework. Victims & Offenders, 17(6), 893–918.

