# Supplementary material for: Barriers and Facilitators for Sexual Trauma Disclosure in Boys and Men: A Systematic Review
Source: Trauma Violence Abuse. 2025 Mar 23;27(3):830–53. doi: 10.1177/15248380251325210 (PMC13287383; doi:10.1177/15248380251325210)
Supplement: sj-docx-1-tva-10.1177_15248380251325210 – Supplemental material for Barriers and Facilitators for Sexual Trauma Disclosure in Boys and Men: A Systematic Review [file sj-docx-1-tva-10.1177_15248380251325210.docx]

**Supplementary File A. Inclusion and exclusion criteria**

Inclusion criteria:

1. Mixed-gender samples exposed to ST *or* boys and/or men exposed to ST *or* professionals (any gender) with experience supporting ST-exposed boys and/or men *or* social supports (any gender) of ST-exposed boys and/or men; and
2. Focus on exposure to ST, including sexual abuse, assaults, and/or coercion; and
3. Study reported perceived barriers and/or facilitators or motivators for ST disclosure *or* study examined variables associated with disclosure likelihood/prevalence *or* study examined variables associated with disclosure timing; and
4. Peer-reviewed studies published in English since 2000.

Exclusion criteria:

1. ST-exposed girls and/or women only *or* mixed-gender ST-exposed samples where key outcomes were not stratified by gender *or* boys and/or men who reported ST perpetration but not exposure *or* professionals and/or social supports with experience supporting ST-exposed people, but with no discussion about boys’ and men’s experiences of ST; and
2. Study examined exposure to sexual harassment *or* exposure to intimate partner violence where ST was not explicitly reported; and
3. Study did not report relevant outcomes *or* study examined relationships between historical ST and health service use, where reasons for service use were not specified; and
4. Grey literature, including theses, dissertations, and non-peer-reviewed reports.
