# Supplementary material for: Barriers and Facilitators for Sexual Trauma Disclosure in Boys and Men: A Systematic Review
Source: Trauma Violence Abuse. 2025 Mar 23;27(3):830–53. doi: 10.1177/15248380251325210 (PMC13287383; doi:10.1177/15248380251325210)
Supplement: sj-docx-2-tva-10.1177_15248380251325210 – Supplemental material for Barriers and Facilitators for Sexual Trauma Disclosure in Boys and Men: A Systematic Review [file sj-docx-2-tva-10.1177_15248380251325210.docx]

**Supplementary File B. Search terms used on each database**

| **Database** | **Search terms** |
| --- | --- |
| PsycInfo | 1 (male or males or boy or boys or men or man or father* or brother* or husband* or son or sons or boyfriend* or masculin*).mp. [mp=title, abstract, heading word, table of contents, key concepts, original title, tests & measures, mesh word]  2 exp Human Males/ or exp "Males (Human)"/ or exp Masculinity/ or exp Gay Males/ or exp Male Only Environments/ or exp Brothers/ or exp Fathers/ or exp Husbands/ or exp Sons/  3 1 or 2  4 ((sex or gender) adj3 difference*).mp. [mp=title, abstract, heading word, table of contents, key concepts, original title, tests & measures, mesh word]  5 exp "Sex Differences (Human)"/ or exp Human Sex Differences/ or exp Gender Differences/  6 4 or 5  7 ((gender adj3 role*) or (gender adj3 norm*) or (gender adj3 strain*)).mp. [mp=title, abstract, heading word, table of contents, key concepts, original title, tests & measures, mesh word]  8 exp Gender Role Attitudes/ or exp Gender Roles/ or Role Strain/  9 7 or 8  10 3 or 6 or 9  11 ((sexual adj3 abuse*) or (sexual adj3 assault*) or (sexual adj3 trauma*) or (sexual adj3 victim*) or (sexual adj3 coerc*) or sexual violence or rape or raped or CSA or victim-survivor).mp. [mp=title, abstract, heading word, table of contents, key concepts, original title, tests & measures, mesh word]  12 exp Sexual Abuse/ or exp Rape/ or exp Sexual Assault/  13 11 or 12  14 ((barrier* or prevent* or inhibit* or obstacle* or delay*) adj3 (report* or disclos* or help-seek* or police or therap* or "service use" or "service usage" or "service engag*" or treatment or "service utilisation" or "service utilization")).mp. [mp=title, abstract, heading word, table of contents, key concepts, original title, tests & measures, mesh word]  15 (non-disclos* or undisclos* or unreport* or underreport* or under-report*).mp. [mp=title, abstract, heading word, table of contents, key concepts, original title, tests & measures, mesh word]  16 exp Abuse Reporting/ or exp Child Abuse Reporting/ or exp Help Seeking Behavior/ or exp Health Care Seeking Behavior/ or exp Health Service Utilization/ or exp Self-Report/ or exp Treatment Barriers/ or exp Communication Barriers/ or exp Health Care Barriers/ or exp Mental Health Care Barriers/  17 ((facilitat* or help* or assist* or support* or encourag*) adj3 (report* or disclos* or help-seek* or police or therap* or "service use" or "service usage" or "service engag*" or treatment or "service utilisation" or "service utilization")).mp. [mp=title, abstract, heading word, table of contents, key concepts, original title, tests & measures, mesh word]  18 14 or 15 or 16 or 17  19 10 and 13 and 18  20 limit 19 to yr="2000 -Current"  21 limit 20 to English language |
| Scopus | ( ( TITLE-ABS-KEY ( ( facilitat* OR help* OR assist* OR support* OR encourag* ) W/3 ( report* OR disclos* OR "help-seek*" OR police OR therap* OR "service use" OR "service usage" OR "service engag*" OR "service utilisation" OR "service utilization" OR treatment ) ) ) OR ( TITLE-ABS-KEY ( ( barrier* OR prevent* OR inhibit* OR obstacle* OR delay* ) W/3 ( report* OR disclos* OR "help-seek*" OR police OR therap* OR "service use" OR "service usage" OR "service engag*" OR "service utilisation" OR "service utilization" OR treatment ) ) ) OR ( TITLE-ABS-KEY ( non-disclos* OR undisclos* OR unreport* OR underreport* OR "under-report*" ) ) ) AND ( TITLE-ABS-KEY ( male OR males OR boy OR boys OR men OR man OR father* OR brother* OR husband* OR son OR sons OR boyfriend* OR masculin* OR "gender W/3 strain*" OR "gender W/3 role*" OR "gender W/3 norm*" OR "gender W/3 difference*" OR "sex w/3 difference*" ) ) AND ( TITLE-ABS-KEY ( "sexual W/3 abuse*" OR "sexual W/3 assault*" OR "sexual W/3 trauma*" OR "sexual W/3 victim*" OR "sexual coerc*" OR "rape" OR "raped" OR "victim-survivor*" OR "sexual violence" ) ) AND ( LIMIT-TO ( LANGUAGE , "English" ) ) AND ( LIMIT-TO ( PUBYEAR , 2022 ) OR LIMIT-TO ( PUBYEAR , 2021 ) OR LIMIT-TO ( PUBYEAR , 2020 ) OR LIMIT-TO ( PUBYEAR , 2019 ) OR LIMIT-TO ( PUBYEAR , 2018 ) OR LIMIT-TO ( PUBYEAR , 2017 ) OR LIMIT-TO ( PUBYEAR , 2016 ) OR LIMIT-TO ( PUBYEAR , 2015 ) OR LIMIT-TO ( PUBYEAR , 2014 ) OR LIMIT-TO ( PUBYEAR , 2013 ) OR LIMIT-TO ( PUBYEAR , 2012 ) OR LIMIT-TO ( PUBYEAR , 2011 ) OR LIMIT-TO ( PUBYEAR , 2010 ) OR LIMIT-TO ( PUBYEAR , 2009 ) OR LIMIT-TO ( PUBYEAR , 2008 ) OR LIMIT-TO ( PUBYEAR , 2007 ) OR LIMIT-TO ( PUBYEAR , 2006 ) OR LIMIT-TO ( PUBYEAR , 2005 ) OR LIMIT-TO ( PUBYEAR , 2004 ) OR LIMIT-TO ( PUBYEAR , 2003 ) OR LIMIT-TO ( PUBYEAR , 2002 ) OR LIMIT-TO ( PUBYEAR , 2001 ) OR LIMIT-TO ( PUBYEAR , 2000 ) ) |
| CINAHL | male OR males OR boy OR boys OR man OR men OR father* OR brother* OR husband* OR son OR sons OR boyfriend* OR masculin* OR gender N3 strain* OR gender N3 role* OR gender N3 norm* OR gender N3 difference* OR sex N3 difference* OR (MH "Male") OR (MH "Men") OR (MH "Gender Role") OR (MH "Masculinity")  AND  sexual N3 abuse* OR sexual N3 assault* OR sexual N3 trauma* OR sexual N3 victim* OR sexual N3 coerc* OR victim-survivor OR rape OR raped OR CSA OR sexual violence OR (MH "Sexual Abuse") OR (MH "Child Abuse, Sexual") OR (MH "Child Abuse Survivors") OR (MH "Sexual Trauma") OR (MH "Sexual Assault Examination" OR (MH "Rape")  AND  ((barrier* OR prevent* OR inhibit* OR obstacle* OR delay*) N3 (report* OR disclos* OR help-seek* OR police OR therap* OR service use OR service usage OR service engag* OR service utilisation OR service utilization OR treatment)) OR ((facilitat* OR help* OR support* OR encourag*) N3 (report* OR disclos* OR help-seek* OR police OR therap* OR service use OR service usage OR service engag* OR service utilisation OR service utilization OR treatment)) OR (MH "Help-Seeking Behavior") OR (MH "Self Disclosure") |
| Medline | *1 (male or males or boy or boys or men or man or father* or brother* or husband* or son or sons or boyfriend* or masculin*).mp. [mp=title, book title, abstract, original title, name of substance word, subject heading word, floating sub-heading word, keyword heading word, organism supplementary concept word, protocol supplementary concept word, rare disease supplementary concept word, unique identifier, synonyms]*  *2 exp Male/ or exp Men/*  *3 1 or 2*  *4 ((sex or gender) adj3 difference*).mp. [mp=title, book title, abstract, original title, name of substance word, subject heading word, floating sub-heading word, keyword heading word, organism supplementary concept word, protocol supplementary concept word, rare disease supplementary concept word, unique identifier, synonyms]*  *5 ((gender adj3 role*) or (gender adj3 norm*) or (gender adj3 strain*)).mp. [mp=title, book title, abstract, original title, name of substance word, subject heading word, floating sub-heading word, keyword heading word, organism supplementary concept word, protocol supplementary concept word, rare disease supplementary concept word, unique identifier, synonyms]*  *6 exp Gender Role/ or exp Masculinity/*  *7 5 or 6*  *8 3 or 4 or 7*  *9 ((sexual adj3 abuse*) or (sexual adj3 assault*) or (sexual adj3 trauma*) or (sexual adj3 victim*) or rape or raped or CSA or "victim-survivor" or (sexual adj3 coerc*) or sexual violence).mp. [mp=title, book title, abstract, original title, name of substance word, subject heading word, floating sub-heading word, keyword heading word, organism supplementary concept word, protocol supplementary concept word, rare disease supplementary concept word, unique identifier, synonyms]*  *10 exp Rape/ or exp Child Abuse, Sexual/ or exp Sex Offenses/ or exp incest/*  *11 9 or 10*  *12 ((barrier* or prevent* or obstacle* or inhibit* or delay*) adj3 (report* or disclos* or help-seek* or police or therap* or "service use" or "service usage" or "service engag*" or treatment or "service utilisation" or "service utilization")).mp. [mp=title, book title, abstract, original title, name of substance word, subject heading word, floating sub-heading word, keyword heading word, organism supplementary concept word, protocol supplementary concept word, rare disease supplementary concept word, unique identifier, synonyms]*  *13 exp Help-Seeking Behavior/ or exp Disclosure/ or exp Self Disclosure/*  *14 (non-disclos* or undisclos* or unreport* or underreport* or under-report*).mp. [mp=title, book title, abstract, original title, name of substance word, subject heading word, floating sub-heading word, keyword heading word, organism supplementary concept word, protocol supplementary concept word, rare disease supplementary concept word, unique identifier, synonyms]*  *15 ((facilitat* or help* or assist* or support* or encourag*) adj3 (report* or disclos* or help-seek* or police or therap* or "service use" or "service usage" or "service engag*" or treatment or "service utilisation" or "service utilization")).mp. [mp=title, book title, abstract, original title, name of substance word, subject heading word, floating sub-heading word, keyword heading word, organism supplementary concept word, protocol supplementary concept word, rare disease supplementary concept word, unique identifier, synonyms]*  *16 12 or 13 or 14 or 15*  *17 8 and 11 and 16*  *18 limit 17 to English language*  *19 limit 18 to yr="2000 -Current"* |
