# Supplementary material for: Barriers and Facilitators for Sexual Trauma Disclosure in Boys and Men: A Systematic Review
Source: Trauma Violence Abuse. 2025 Mar 23;27(3):830–53. doi: 10.1177/15248380251325210 (PMC13287383; doi:10.1177/15248380251325210)
Supplement: sj-docx-3-tva-10.1177_15248380251325210 – Supplemental material for Barriers and Facilitators for Sexual Trauma Disclosure in Boys and Men: A Systematic Review [file sj-docx-3-tva-10.1177_15248380251325210.docx]

**Supplementary File D. Assessed and endorsed disclosure barriers from k = 9 articles that presented disclosure barrier items and reported the prevalence of boys and men endorsing each item**

| **Grouped disclosure barriers** | **Barrier to disclosure item used** | **Prevalence of endorsement in each article** |
| --- | --- | --- |
| Shame/ embarrassment | I would feel ashamed to talk to my provider about MST; It is embarrassing to receive services for MST^1^ (Hahn et al., 2021) | 64.9% |
|  | Embarrassed for self or family (Velloza et al., 2022) | 54.6% |
|  | I was ashamed of what happened and didn't want anyone to know (Postmus et al., 2015) | 46.2% |
|  | It would feel like an admission of failure (Adejimi, Sabageh, & Adedokun, 2016) | 44.7% |
|  | I felt ashamed and embarrassed (Adejimi et al., 2016) | 26.3% |
|  | I was embarrassed (Frías & Erviti, 2014) | 25.7% |
|  | I felt ashamed (Lehrer, Lehrer, & Koss, 2013) | 14.3% |
|  | Afraid/ashamed (Broban et al., 2020) | 11.9% |
|  | I felt dirty (Frías & Erviti, 2014) | 3.5% |
|  | Other people would find out I experienced MST (Hahn et al., 2021) | NR^3^ |
| Concerns about unsupportive responses | I would not be believed without evidence (Hahn et al., 2021) | 55.9% |
|  | I didn’t think anyone would believe me (Postmus et al., 2015) | 45.4% |
|  | No one cares about my experiences (Hahn et al., 2021) | 40.8% |
|  | I was afraid of what would happen to my family (Postmus et al., 2015) | 38.5% |
|  | It’s no use reporting to superiors (Kwon et al., 2007) | 29.3% |
|  | There was no reason to say it to anybody (Frías & Erviti, 2014) | 28.8% |
|  | I thought nobody would believe me (Frías & Erviti, 2014) | 11.1% |
|  | If I told the police they would not respond (Lehrer et al., 2013) | 7.1% |
|  | I thought I would be blamed (Adejimi et al., 2016) | 5.3% |
|  | I was scared of not being believed (Adejimi et al., 2016) | 5.3% |
|  | I was afraid that people would not believe me (Okur, van der Knaap, & Bogaerts, 2020) | 3.1% |
|  | I was afraid to be blamed (Okur et al., 2020) | 1.6% |
|  | I thought I would not be believed (Lehrer et al., 2013) | NR^2^ |
|  | My VA provider would not believe me (Hahn et al., 2021) | NR^3^ |
|  | My provider would blame me for the events (Hahn et al., 2021) | NR^3^ |
|  | Other people might think I caused the event to happen (Hahn et al., 2021) | NR^3^ |
|  | My provider would react negatively (Hahn et al., 2021) | NR^3^ |
|  | My provider would not want to hear about my problems (Hahn et al., 2021) | NR^3^ |
|  | I was afraid of not being treated well by the justice system (Lehrer et al., 2013) | NR^2^ |
| Logistical factors | It is better to seek services outside of the VA for MST; I would not receive good mental health services for MST; Staff is not well-trained to handle problems related to MST^4^ (Hahn et al., 2021) | 41.3% |
|  | No knowledge about treatment (Broban et al., 2020) | 18.6% |
|  | No access to healthcare (Broban et al., 2020) | 17.1% |
|  | I did not know the reporting procedure (Adejimi et al., 2016) | 11.6% |
|  | I found it too much hassle (Okur et al., 2020) | 4.7% |
|  | I did not know where to seek help (Okur et al., 2020) | 2.2% |
|  | I did not know what to do (Okur et al., 2020) | 0.0% |
|  | The insurance did not cover any expenses (Okur et al., 2020) | 0.0% |
|  | There was a long waiting list for mental care (Okur et al., 2020) | 0.0% |
|  | I found it too expensive (Okur et al., 2020) | 0.0% |
|  | I did not know how to go to the police (Lehrer et al., 2013) | NR^2^ |
| Minimization | I didn't think it was a problem because it occurs so commonly (Kwon et al., 2007) | 82.7% |
|  | I did not think what happened to me was sufficiently serious or a crime (Lehrer et al., 2013) | 50.0% |
|  | Did not view the experience as a problem (Velloza et al., 2022) | 46.2% |
|  | I thought it was not severe enough (informal reporting) (Okur et al., 2020) | 25.0% |
|  | I thought it was not severe enough (formal reporting) (Okur et al., 2020) | 23.9% |
| Perpetrator relationships | I was afraid I would get the person who did it to me in trouble (Postmus et al., 2015) | 43.2% |
|  | Because of my relationship to the perpetrator (Kwon et al., 2007) | 30.7% |
|  | I was not sure that the person who did this really meant to harm me (Lehrer et al., 2013) | 14.3% |
|  | I loved the person who did it (Frías & Erviti, 2014) | 11.1% |
|  | I did not want the perpetrator to get into trouble (Adejimi et al., 2016) | 7.9% |
|  | I did not want it to have negative consequences for the perpetrator (Okur et al., 2020) | 0.0% |
|  | I did not want my relationship with the person to end (Lehrer et al., 2013) | NR^2^ |
| Retaliation concerns | I was scared of revenge by the perpetrator (Adejimi et al., 2016) | 36.8% |
|  | Feared retaliation (Kwon et al., 2007) | 13.3% |
|  | Fear of retaliation by the person who did it (Lehrer et al., 2013) | 9.5% |
|  | I was threatened (Frías & Erviti, 2014) | 1.8% |
| Punishment concerns | I was afraid of getting into trouble (Postmus et al., 2015) | 45.3% |
|  | I was afraid I would get kicked out of school (Postmus et al., 2015) | 35.1% |
|  | I thought I would be admonished (Frías & Erviti, 2014) | 15.0% |
| Negative help-seeking attitudes | It's better to avoid talking about or seek help for mental health problems (Hahn et al., 2021) | 21.4% |
|  | I found it difficult to ask for help (Okur et al., 2020) | 2.2% |
|  | I did not dare to seek formal help (Okur et al., 2020) | 2.2% |
|  | I did not trust formal health counsellors (Okur et al., 2020) | 1.1% |
|  | Other people would find out I was seeking mental health care (Hahn et al., 2021) | NR^3^ |
| Self-reliance or stoicism | I did not need formal help (Okur et al., 2020) | 73.9% |
|  | Tough people can handle their problems on their own; It is better to cover up how I feel^5^ (Hahn et al., 2021) | 59.6% |
|  | I would not be able to control my emotions (Hahn et al., 2021) | 55.5% |
|  | I thought it was unnecessary (Okur et al., 2020) | 46.9% |
|  | I took care of it myself (Okur et al., 2020) | 17.2% |
|  | Other people would think I was weak (Hahn et al., 2021) | NR^3^ |
| Privacy | I felt it was a private issue and wanted to deal with it personally (Adejimi et al., 2016) | 60.5% |
|  | I thought it was something very personal (Frías & Erviti, 2014) | 27.9% |
|  | I did not want my family and friends to find out (Okur et al., 2020) | 3.1% |
|  | The provider would not keep it confidential (Hahn et al., 2021) | NR^3^ |
| Other | Other people would make judgments about my sexuality (Hahn et al., 2021) | 59.1% |
|  | It is embarrassing to talk to a person of the same sex about MST (Hahn et al., 2021) | 54.6% |
|  | The large number of men at the VA makes me uncomfortable (Hahn et al., 2021) | 48.1% |
|  | I did not think others would understand (Adejimi et al., 2016) | 44.7% |
|  | I was afraid of what would happen to me (Postmus et al., 2015) | 41.5% |
|  | I wanted to forget the sexual assault (Adejimi et al., 2016) | 39.5% |
|  | I was afraid (Frías & Erviti, 2014) | 27.9% |
|  | It is embarrassing to talk to a person of the opposite sex about MST (Hahn et al., 2021) | 26.3% |
|  | I felt guilty (Frías & Erviti, 2014) | 5.8% |
|  | I was afraid of gossip (Okur et al., 2020) | 4.7% |
| *Note.* NR = not reported. VA = Veterans Administration. ^1^ These two items were grouped by the authors under “internal stigma”; percentage of endorsement reflects these two items collectively. ^2^ In Lehrer et al. (2013), authors did not report prevalence of endorsement for items endorsed by less than 7.1% of participants. ^3^ Prevalence of endorsement for this item unavailable because authors grouped conceptually distinct disclosure barrier items and reported prevalence of endorsement collectively. ^4^ These three items were grouped by the authors under “staff skill”; percentage of endorsement reflects these three items collectively. ^5^ These two items were grouped by the authors under “self-reliance”; percentage of endorsement reflects these two items collectively. Full list of disclosure barriers not reported in Velloza et al. (2022). | | |
