# Supplementary material for: Barriers and Facilitators for Sexual Trauma Disclosure in Boys and Men: A Systematic Review
Source: Trauma Violence Abuse. 2025 Mar 23;27(3):830–53. doi: 10.1177/15248380251325210 (PMC13287383; doi:10.1177/15248380251325210)
Supplement: sj-docx-4-tva-10.1177_15248380251325210 – Supplemental material for Barriers and Facilitators for Sexual Trauma Disclosure in Boys and Men: A Systematic Review [file sj-docx-4-tva-10.1177_15248380251325210.docx]

**Supplementary File E. Variables assessed as possible predictors of disclosure likelihood in ST-exposed boys ad men (k = 15)**

| **Authors; year; location** | **Sample size; gender; age range, mean age** | **Disclosure source(s) assessed** | **Study method; analyses** | **Potential correlates assessed** | **Correlates of disclosure likelihood in boys/men** |
| --- | --- | --- | --- | --- | --- |
| Boudreau et al.; 2018; Kenya | n = 489; mixed gender (n = 185 boys/men); range = 13 - 24 years, *M* = 18.4 years | Any source | Cross-sectional survey; logistic regression | Age at survey, single or double orphan prior to ST, age of ST exposure, number of ST experiences, ST type (physically forced sex Y/N), emotional violence exposure before ST, physical violence exposure before ST, perpetrator relationships (romantic partner Y/N; family member Y/N), perpetrator familiarity | None associated in unadjusted or adjusted models |
| Canan et al.; 2023; United States | n = 2,013; mixed gender (n = 232); range and mean NR | Family member(s) | Cross-sectional survey; chi-square | Sexuality (gay, bisexual, heterosexual) | Higher disclosure to family members in gay (16.9%) and heterosexual men (14.6%), relative to bisexual men (1.8%), χ^2^ = 7.8, *p* = .020, Cramer’s V = 0.19. |
| Coxell et al.; 2000; United Kingdom | n = 37; all men; range = NR, *M* = 31 years | Any source | Cross-sectional survey; NR | Perpetrator sex (male vs female) | Not associated with disclosure |
| Easton; 2013; United States | n = 487; all men; range = 19 - 84 years, *M* = 50.4 years | Any source; a spouse or partner; formal authorities (police or child protective services) | Cross-sectional survey; chi-square and logistic regression | Clergy member vs non clergy member perpetrator, family member vs non-family perpetrator, age at time of study | Higher likelihood of reporting to authorities with clergy vs non-clergy perpetrators (20% vs 8%, χ^2^ = 12.51, *p*< .001). Family-perpetrated CSA associated with: lower likelihood of reporting to authorities (6% vs 16%, χ^2^ = 4.26, *p*< .05), childhood disclosure (15% vs 27%, χ^2^ = 4.03, *p*< .05), disclosure to spouse/partner (77% vs 87%, χ^2^ = 13.68, *p*< .01) or in-depth discussion with spouse/partner (57% vs 68%; χ^2^ = 8.84, *p* < .05). Reporting to authorities less likely with older age (OR = 0.963, *p* < .01). |
| Eisenberg et al.; 2021; United States | n = 551; mixed gender (n = 74 boys/men); range and mean NR | Medical practitioners; university authority; police; social supports (friend, partner, family, or other) | Cross-sectional survey; chi-square | Sexuality (heterosexual vs sexual minority) | Not associated with disclosure |
| Hanson et al.; 2003; United States | n = 326; mixed gender (n = 71 boys); range = 12-17 years, mean NR | Any source | Cross-sectional survey; logistic regression | Race/ethnicity (African American vs White, Hispanic vs White), perceived life threat during CSA, substance-facilitated CSA, single vs multiple-occasion assault, known vs unknown perpetrator, penetrative assault | Lower disclosure likelihood in African American vs White boys (OR = 0.19, *p* < .05, 95% CI: 0.04 - 0.96) |
| Hershkowitz et al.; 2005; Israel | n = 10,988; mixed gender (n = 3,416 boys); age range = 3 - 14 years, mean NR | Investigative interviewers | Analysis of archival investigative interview data; log linear analysis and descriptive statistics | Perpetrator relationship (parent figure vs non-parent figure) and age (3-6, 7-10, 11-14) | Boys less likely than girls to allege CSA with parent-figure suspected perpetrators; result seen in all three age groups (3-6 years: 12.3% boys vs 16.7% girls, *p* < .017; 7 - 10 years: 16.9% vs 22.2%, *p* = .009; 11 - 14 years: 12.4% vs 34.1%, *p* < .001). Higher allegation rates in older vs younger boys (3 - 6 years: 47.0%, 7-10 years: 72.6%, 11 - 14 years: 77.3%).  Lower allegation rates when suspected perpetrators were parent vs non-parent figures (14.2% vs 87.4%; statistics NR) |
| Hietamäki et al.; 2024; Finland | n = 537; mixed gender (n = 79 boys); range = 11 - 17 years; mean NR | Any source | Cross-sectional survey; chi-square | Perpetrator sex (male vs female) | Lower rates of disclosure observed in boys assaulted by male vs female perpetrators (non-disclosure: 56% vs 27% respectively, χ^2^ = NR, *p* = .024). |
| Masho & Alvanzo; 2010; United States | n = 91; all boys/men; age range NR, *M* = 42.4 years | Medical practitioners, mental health practitioners, or sexual assault services | Cross-sectional survey; logistic regression | Threatened during assault, injured during assault, family member/friend perpetrator, childhood or adulthood ST | Higher disclosure likelihood with threats (aOR = 7.08, 95% CI: 1.52 - 33.03, *p* < .05), injuries (aOR = 6.58, 95% CI: 1.08 - 40.19, *p* < .05), and family/friend perpetrators (aOR = 6.42, 95% CI: 1.47 - 28.04, *p* < .01) |
| Nofziger & Stein; 2006; United States | n = 326; mixed gender (n = 71 boys); range = 12 - 17 years, mean NR | Any source | Cross-sectional survey; logistic regression | Age of ST exposure, deviance, number of witnessed violence types, ST context (home, school, neighbourhood, other), fear of serious injury/death | Lower disclosure likelihood with older age of exposure (OR = 0.73, SE = 0.12, *p* < .01). |
| Priebe & Svedin; 2008; Sweden | n = 1,493; mixed gender (n = 249 boys); range and mean NR | Any source, friend of my age, mother, father, sibling, professional, adult relative/friend, social authorities/police, other person | Cross-sectional survey; logistic regression | ST type (non-contact vs contact or penetrative), frequency of ST (once, 2-5 occasions, more than 5 occasions), perpetrator relationship (stranger, family/relative, friend/acquaintance), offender used alcohol or drugs (Y/N), immigrant (Y/N), education (academic or vocational), family structure (living with both parents or not living with both parents), parental bonding (high care low overprotection, high care high overprotection, low care low overprotection, low care high overprotection) | Higher likelihood of non-disclosure (any source) with vocational education (aOR = 3.20, 95% CI: 1.65 - 6.21, *p* = .001), living with both parents (aOR = 0.43, 95% CI: 0.22 - 0.86, *p* = .017), and parents perceived as: caring and overprotective (aOR = 2.76, 95% CI: 1.12 - 6.80, *p* = .028) or not caring and not overprotective (aOR = 3.20, 95% CI: 1.49 - 6.91, *p =* .003). |
| Priebe & Svedin; 2012; Sweden | n = 576; mixed gender (n = 112 boys/men); range NR, *M* = 18.3 years | Any source | Cross-sectional survey; logistic regression | Sexuality (heterosexual vs sexual minority) | Not associated with disclosure |
| Velloza et al.; 2022; Namibia | n = 675; mixed gender (n = 101 boys/men); range = 13 - 24 years, mean NR | Any source | Cross-sectional survey; logistic regression | Age (13-14, 15-19, 20-24), highest level of education (primary or less, secondary to grade 10, grade 11 or higher), communication with parents^1^ (easy vs difficult), close relationship with friends (Y/N), ever had sex, number of experienced violence types (1, 2, 3 or more), perpetrator relationship (family member, romantic partner or spouse, friend or neighbour or classmate, authority figure or other adult in community), endorsed any normative beliefs about domestic violence^2^, ever witnessed physical violence in home or community | Higher disclosure likelihood with close friendships (aOR = 3.03; 95% CI: 1.00 - 6.98, *p* = .04), exposure to multiple violence types (aOR = 6.83, 95% CI: 1.45 - 32.08, *p* = .04) and witnessed physical violence (aOR = 1.89; 95% CI: 1.14 - 3.13, *p* = .04) |
| Walfield, McCormack, & Clarke; 2024; United States | n = 330; all men; range NR; *M* = 33.9 years | Police | Cross-sectional survey; logistic regression | Age of ST exposure, perpetrator age (juvenile vs adult), perpetrator familiarity (known vs unknown), perpetrator relationship (current/former intimate partner Y/N), college education (two-year associate degree vs some or no college), living in rural area (Y/N), single- vs repeated-event assault, weapon used (Y/N), physical injury sustained (Y/N), assault during day vs night, offender used substances prior to assault (Y/N), location of assault (a home vs another location), ST type (completed rape vs other) | Higher reporting likelihood with older vs younger age of ST exposure (OR = 1.79, 95% CI: 1.25 - 2.58, *p*< .01) and when assaults involved weapons vs no weapons (OR = 4.54, 95% CI: 1.74 - 11.82, *p*< .001). Lower reporting likelihood with female vs male perpetrators (OR = 0.36, 95% CI: 0.13 - 0.99, *p*< .05), juvenile vs adult offenders (OR = 0.15, 95% CI: 0.03 - 0.81, *p*< .05), and college education vs not started/completed college education (OR = 0.38, 95% CI: 0.19 - 0.77, *p*< .01). |
| Weiss; 2010; United States | n = 1,050; mixed gender (n = 94 boys and men); range and mean NR | Police | Cross-sectional survey; chi-square | Perpetrator sex (male vs female) | Higher proportion of police reporting with male-perpetrated (21.6%) vs female-perpetrated (7.0%) assaults (χ^2^ = 3.919, *p*< .05). |
| Note. NR = not reported. ^1^Items from Health Behaviour in School-Aged Children scale (Lenciauskiene & Zaborskis, 2008). ^2^ Items such as “is a husband justified in hitting or beating his wife if she refuses to have sex with him” and “is a husband justified in hitting or beating his wife if she argues with him?”. | | | | | |
