# Supplementary material for: Barriers and Facilitators for Sexual Trauma Disclosure in Boys and Men: A Systematic Review
Source: Trauma Violence Abuse. 2025 Mar 23;27(3):830–53. doi: 10.1177/15248380251325210 (PMC13287383; doi:10.1177/15248380251325210)
Supplement: sj-docx-5-tva-10.1177_15248380251325210 – Supplemental material for Barriers and Facilitators for Sexual Trauma Disclosure in Boys and Men: A Systematic Review [file sj-docx-5-tva-10.1177_15248380251325210.docx]

**Supplementary File F. Variables assessed as possible predictors of disclosure timing in ST-exposed boys and men (k = 5)**

| **Authors; year; location** | **Sample size; gender; age range, mean age** | **Disclosure source(s) assessed; disclosure timing outcome** | **Study method; analysis method** | **Disclosure correlates assessed** | **Correlates of disclosure timing for boys/men** |
| --- | --- | --- | --- | --- | --- |
| Broban et al.; 2020; 7 countries across African continent | n = 13,550; mixed gender (n = 1,009 boys/men); range and mean NR | Sexual assault services; disclosure timing categorised into: within 72 hours of ST, 72 hours-1 month after ST, or one month or longer after ST | Analysis of archival healthcare data; Cuzick's test for trend | Age at presentation to sexual assault service (0 - 12 years, 13 - 19 years, 20 - 45 years, > 45 years) | Younger age associated with help-seeking 72 hours-1 month after ST (*p* < .001) and older age associated with help-seeking one month or longer after ST (*p* < .01). No relationship between age and presenting to care within 72 hours. |
| Cashmore et al.; 2017; Australia | n = 122,757; mixed gender (number of boys NR); range and mean NR | Police; childhood vs adult reporting | Analysis of archival crime data from New South Wales and South Australia; chi-square | Age at ST (5 or younger, 6 - 9, 10 - 13, 14 - 17, multiple ages), ST type (sexual assault, indecent assault, acts of indecency), perpetrator relationship (parent/guardian, sibling, other family member, member of household, boyfriend or girlfriend, other known person, authority figure^1^, not known) | Delayed reporting higher in older boys (New South Wales: χ^2^ = 165.9, *p* < .0001; South Australia: χ^2^ = 165.9, *p* < .0001). More boys vs girls with authority-figure perpetrators delayed disclosure for 20+ years in New South Wales, 65% vs 27.8%; no inferential statistics reported. |
| Easton; 2013; United States | n = 487; all men; range = 19-84 years, *M* = 50.4 years | Any source; number of years until first told (continuous), number of years until first in-depth discussion and disclosed during childhood (Y/N) | Cross-sectional survey; t-tests and correlation | Clergy member vs non clergy member perpetrator, family member vs non-family perpetrator, age at time of study | Longer delays with family member vs non-family perpetrators, t (71) = -2.16, *p* < .05. Age positively correlated with years until first disclosure (r = 0.328, *p* < .001) and years until first in-depth discussion (r = 0.547, *p* < .001) |
| Gundlapalli et al.; 2019; United States | n = 1,730; mixed gender (n = 112 men); range and mean NR | Veterans’ Health Administration; initial negative screen for MST accompanied by subsequent (next 12 months) evidence of MST in medical notes | Natural language processing analysis of medical records; logistic regression | Race/ethnicity (non-Hispanic White, non-Hispanic Black, White, or other), age at time of study (19-30, 31-40, 41-50, 51+), education more than high school Y/N, marital status (married, previously married, never married), branch of military service (army, air force, navy, marines, coast guard), active duty Y/N, officer or warrant rank Y/N | Delayed disclosure less likely in active duty vs non-active duty (aOR = 0.29, 95% CI: 0.13 - 0.68, *p* = .004) |
| Romano et al.; 2019; United States and Canada | n = 253; all men; range = 18-59 years, *M* = 39.5 years | Any source; number of years until first told (continuous) | Cross-sectional survey; Pearson correlation coefficients | Age of first disclosure, disclosure reaction^2^, number of people told, CSA severity^3^, number of other maltreatment types, number of other (non-trauma) adversities, resilient functioning, internalising difficulties, externalising difficulties, substance use difficulties | Longer delays positively correlated with older age at first disclosure (r = .82, *p* < .01), externalising difficulties (r = .15, *p*< .05), substance use difficulties (r = .14, *p* < .05), supportive vs unsupportive disclosure reactions (r = .16, *p* < .05) |
| *Note.* ^1^ Included teachers, clergy members, carers, or youth leaders. ^2^ For the disclosure reaction question, participants were asked a series of dichotomous questions about whether the first person they had told had blamed them, supported them, not believed them, ignored them, or other. ^3^ Rated on a five-point scale, where one point was given for each of the following: abuse lasting longer than one month, abuse onset prior to age 12, family member perpetrator, abuse involved physical contact beyond fondling, and perpetrator use of physical violence or threat of violence. | | | | | |
