# Supplementary material for: Barriers and Facilitators for Sexual Trauma Disclosure in Boys and Men: A Systematic Review
Source: Trauma Violence Abuse. 2025 Mar 23;27(3):830–53. doi: 10.1177/15248380251325210 (PMC13287383; doi:10.1177/15248380251325210)
Supplement: sj-docx-6-tva-10.1177_15248380251325210 – Supplemental material for Barriers and Facilitators for Sexual Trauma Disclosure in Boys and Men: A Systematic Review [file sj-docx-6-tva-10.1177_15248380251325210.docx]

**Supplementary File G. Barriers and facilitators for disclosure (reported in k = 42 qualitative articles and mixed-method article Guerra et al., 2021)**

| **Authors; year; location** | **Sample description; sample size; gender; age range, mean age** | **Key findings: barriers** | **Key findings: facilitators and/or motivators** |
| --- | --- | --- | --- |
| Alaggia; 2005; Canada | CSA-exposed members of general community; n = 30; mixed gender (n = 11 men); range = 18 - 65 years, *M* = 40.1 years | Men: concerns about being labelled gay, not wanting to be viewed as victims, and invisibility of men’s ST. Women: higher self-blame and anticipated blame and disbelief. | Reaching a “breakdown” or crisis point^1^ (both genders) and fear of becoming a perpetrator themselves (men only). |
| Aspin et al.; 2009; New Zealand | ST-exposed Māori men who have sex with men; n = 8; all men; range = 24 - 50 years, mean NR | Lack of appropriate services (including culturally appropriate services), perceived futility in reporting due to lack of faith that authorities and institutions would respond, uncertainty about characterising experiences as ST, invisibility of men’s ST. | N/A – not assessed |
| Attrash-Najjar et al.; 2023; Israel | ST-exposed men who submitted narratives to Israeli Public Inquiry of CSA; n = 51; all men; range = 18 - 60 years; *M* = 35 years | Shame, confusion and difficulty recognising abuse (including due to abuse being normalised as part of daily routines in educational settings and/or occurring in public spaces), avoidance and repression of trauma memories, concerns about being labelled gay, concerns about tangible consequences (inability to progress in military), not wanting to go through justice system, limited recognition among others about boys' behavioural changes following abuse, poor disclosure reactions from informal and formal supports (indifference, lack of follow-up, disbelief, lack of connections to support services), isolation/lack of supports | Reaching a crisis point (significant post-traumatic stress symptoms) |
| Braun et al.; 2009; New Zealand | ST-exposed gay and bisexual men; n = 19; all men; range = 20 - 54 years; mean NR | Concerns about not being believed, particularly when men had interacted/spent time with perpetrators prior to assaults (e.g., interacting at a gay club, going home together), normalising assaults when repeated in intimate partner relationships. | N/A – not assessed |
| Christian et al.; 2011; Democratic Republic of Congo | ST-exposed men in conflict and post-conflict rural villages, social supports of ST-exposed men (family members and friends), and support workers (medical and social healthcare practitioners); n = 30; mixed gender (n = 7 ST-exposed men); age range = 25 - 74 years, mean NR | Shame, perceived loss of masculine identity following ST, concerns about ridicule and ostracization from community (for self and family), confidentiality concerns, cost of accessing support, lack of appropriate services for men, poor health service resourcing (limited practitioner capacity, particularly for men; limited supplies in health centres and hospitals; lack of services for mental health care; insufficient resourcing for follow-up), lack of appropriate training for healthcare practitioners, ST as taboo (particularly when experienced by men). | Reaching a crisis point and being encouraged or forced to seek help by family members. |
| Chynoweth et al.; 2020; Italy, Bangladesh and Kenya | ST-exposed refugees and support workers (aid workers and human rights experts); n = 458 (n = 148 support workers and n = 310 refugees); mixed gender (number of ST-exposed boys/men NR); range and mean NR | Difficulty recognising or labelling events as ST, internalised blame (particularly among sexual and gender minority men), concerns about compromising residency status and access to other benefits, safety concerns (perpetrator retaliation), legal definition of rape excluded men, laws prohibiting same-sex relationships, lack of appropriate services and referral points for men, poor referral systems, financial cost of accessing support, transport costs and difficulties (particularly for men with disabilities), limited knowledge about support services, uncertainty about the benefits of accessing support, lack of appropriate training for healthcare practitioners, anticipating negative attitudes and reactions from service providers (particularly among gay and bisexual men and men with disabilities), confidentiality concerns, concerns about humiliation, loss of social relationships, and ostracization from community (for self and family). | N/A – not assessed |
| Corboz et al.; 2023; Afghanistan | ST-exposed men and support workers (medical practitioners, mental health practitioners, community health workers); n = 91 (n = 27 ST-exposed men); range = 18 - 40 years; *M* = 25 years | Limited services for boys and men, uncertainty about where to obtain support, limited practitioner knowledge, lack of processes and protocols for responding to boys' and men's reports (distinct from girls and women), shame, internalised blame, concerns about impacts on social standing (including personal and family reputations) and potential ostracization from communities, fear of perpetrator retaliation, fear of violence/retaliation from disclosure sources (family members and religious leaders; particularly due to laws prohibiting same-sex sexual activity), concerns about - and experiences of - negative reactions from health practitioners (e.g., attributing blame, eliciting shame), confidentiality concerns (including practitioners informing family, community, and/or law enforcement), lack of safety for gender diverse people in healthcare settings and local culture. | N/A – not assessed |
| Donne et al.; 2018; United States | ST-exposed (past 12 months) men from general community; n = 32; all men; age range = 21-47 years, *M* = 32 years | Difficulty recognising or labelling events as ST, avoidance, preference for self-reliance, desire to uphold masculine norms, concerns about social consequences, confidentiality concerns, being gay and help-seeking as “double stigma”, lack of normalisation of mental health help-seeking in Black community, avoiding trauma memories and painful emotions, financial cost of accessing support, insurance issues, scheduling issues, struggling to find practitioner who was “the right fit”. | Reaching a crisis point in the form of gradually deteriorating mental health, or acute deterioration in mental health (after seeing the perpetrator unexpectedly). |
| Easton, Saltzman, & Willis; 2014; United States^2^ | CSA-exposed men from general community; n = 460; all men; range = 19-84 years, *M* = 50.7 years | Desire to uphold masculine norms (did not want to be viewed as weak or feminine), avoiding experiencing and expressing strong emotions during disclosure, expectations surrounding men’s sexual desire, lack of appropriate services for men, concerns about rejection or loss/change of relationships, experiencing negative responses to disclosures previously (accusations of false allegations, minimising reports, attributing blame), concerns about being labelled future perpetrators, perceived futility in reporting, difficulty trusting others, sexuality concerns (not wanting to be labelled gay; not wanting sexuality to be attributed to ST), perpetrator threats (to tell others and that boys would go to hell if they told), concerns about loss of housing or employment, safety concerns (physical violence from family), ST as taboo, desire to “protect” loved ones, guilt and self-blame, shame and embarrassment, feelings of disgust and worthlessness, difficulty labelling events as ST, denial and repressed memories. | N/A – not assessed |
| Elder et al.; 2017; United States | ST-exposed veterans; n = 21; all men; range = 29 - 70 years; mean NR | Not wanting to be labelled gay (discussed in relation to violating masculine norms/ideals), concerns about unsupportive responses (being blamed), ST viewed as violating masculine norms and not wanting to be viewed as weak or vulnerable | N/A – not assessed |
| Forde & Duvvury; 2017; Republic of Ireland | ST-exposed rape crisis centre users; n = 5; all men; range = 28 - 56 years; *M* = 44.6 years | Repressed memories and difficulty accepting status as victim-survivors, avoided and suppressed memories and feelings associated with ST (including by using alcohol and drugs and engaging in antisocial and violent behaviour), shame, perceived dissonance between ST and masculine norms and lack of awareness men experience ST, difficulties trusting others, negative past experiences of disclosure (from police, legal professionals, healthcare practitioners, informal supports), ST against men as taboo | Engagement with other mental health supports/organisations, being encouraged to seek support by other practitioners (general practitioner), concerns about impacts of ST on current behaviour and mental health (including aggression and introversion), desire to challenge belief that men cannot be abused. |
| Foster; 2017a; United States | ST-exposed mental health service users; n = 19; all boys; range = 3 - 17 years, *M* = 8.5 years | Shame and embarrassment, sexuality concerns (not wanting to be labelled gay), safety concerns (perpetrator retaliation), concerns about perpetrators experiencing punishment or consequences, uncertainty about how others would respond. | Being asked directly by a caregiver (mother). |
| Foster; 2017b; United States | ST-exposed mental health service users; n = 19; all boys; range = 3 - 17 years, *M* = 8.5 years | Safety concerns (perpetrator retaliation), sexuality concerns (not wanting to be labelled gay), uncertainty about how others would react, confidentiality concerns (including beliefs that telling a caregiver would lead to immediate police reporting), not wanting to relive trauma during counselling. | Encouragement from social supports (mothers) to begin and continue attending counselling, sustained help-seeking became easier through developing trusting relationships with counsellors. |
| Gagnier & Collin-Vezina; 2016; Canada | Sexual assault service users; n = 17; all men; range = 19-67 years, *M* = 47 years | Safety concerns (perpetrator retaliation), negative previous disclosure experiences (lack of response, reports not taken seriously), not wanting to disappoint family members/others, ST as taboo, expectation for men to desire heterosexual sexual activity, difficulty recognising or labelling events as ST (due to uncertainty about whether events could be characterised as ST, particularly when perpetrated by women), denial, repressed memories, desire to uphold masculine norms (not wanting to be labelled victims, pressure to demonstrate stoicism and self-reliance), concerns about being labelled future perpetrators. | Hearing others' experiences (including social supports, other men, and figures in popular media), presence of trusting relationships, subsequent disclosures became easier after the first disclosure. |
| Gagnier, Collin-Vezina, & De La Sablonniere-Griffin; 2017; Canada | Sexual assault service users; n = 17; all men; range = 19-67 years, *M* = 47 years | Lack of trust in child protection and law enforcement services, long waitlists, limited appropriate supports for men, the lack of men’s representation in ST resources and materials. | Help-seeking facilitated by referrals from other professionals, visibility of relevant services, loved ones encouraging help-seeking (spouses, partners, family members, friends) and popular media highlighting other men's stories. One motivator for help-seeking reported: seeking support to ensure relationships were not negatively impacted by CSA and related problems. |
| Gill & Begum; 2023; United Kingdom | Sexual assault service and website users; n = 8; all men; range and mean NR | Concerns about unsupportive responses from family members (disbelief, limited understanding), perception disclosure would "let down" family, concerns about impacts on family reputation (particularly given close community relationships), previous unsupportive disclosure reactions (including discouraging subsequent disclosures), perpetrator encouraging silence and secrecy, not wanting to upset loved ones, not wanting to be labelled gay (and belief family would conflate abuse with consensual same-sex sexual activity), taboo surrounding discussing ST (particularly as men), cultural stigma surrounding men's victimisation. | Presence of trusted supports, particularly romantic partners (some described feeling safer to disclose to partners than family members) |
| Gruenfield, Willis, & Easton; 2017; International | Mental health practitioners with experience supporting CSA-exposed men; n = 9; all men; range and mean NR | Self-blame, shame, difficulty recognising and labelling events as ST, beliefs that men should not experience ST, desire to uphold masculine norms (not wanting to be viewed as weak or victims, concerns about being labelled future perpetrators, expectations for men to desire heterosexual sexual activity, sexuality concerns (not wanting to be labelled gay), negative previous disclosure experiences (blamed, shamed, disbelieved, lack of response) and negative past experiences in therapy, concerns about loss or negative impacts on relationships, concerns about impacts on job security, difficulty establishing trusting relationships with therapists, perceived power imbalance between clients and therapists, time-limited provision of support, and limited appropriate services for men. | N/A – not assessed |
| Guerra et al.; 2021; Chile | Sexual assault service users; qualitative phase: n = 10, mixed gender (n = 3 men), age range = 18 - 20 years, *M* = 18.8 years | For men and women, barriers included guilt, shame, concerns about negative reactions (including disbelief and punishment), safety concerns (perpetrator retaliation), not wanting to upset loved ones, and difficulty recognising or labelling events as ST. For men, participants described the invisibility of men’s ST and how masculine gender stereotypes in Chile (expectations for men to be dominant, virile, strong) impeded disclosure. Help-seeking was viewed as reflecting weakness and violating masculine norms, and men experienced concerns about being labelled gay. | N/A – not assessed |
| Hlavka; 2017; United States | ST-exposed; n = 31; all boys; range = 5-17 years, mean NR | Difficulty labelling event as ST, desire to uphold masculine norms, sexuality concerns (not wanting to be labelled gay), perpetrators threatening to tell others, shame, minimisation (particularly when ST perpetrated by women), expectations for men to desire heterosexual sexual activity, invisibility of men’s ST, concern about punishment due to illicit substance use at the time of ST, desire to protect loved ones. | One participant reported ST following perpetrator threatening physical violence and fears for their physical safety. |
| Holland & Cipriano; 2021; United States | ST-exposed undergraduate university students; n = 40; mixed gender (n = 7 men); range = 19 - 25 years, *M* = 19.77 years | Men reported minimisation, anticipating negative responses (reports being questioned and lack of response), concerns about loss of scholarship due to drinking at the time of the ST, and loss of social relationships if ST was disclosed or discovered. Unlike women, men did not describe poor knowledge about Title IX Office or perpetrator locality (i.e., not attending the university) as disclosure barries. | N/A – not assessed |
| Hunter; 2011; Australia | ST-exposed; n = 22; mixed gender (n = 9 men); range = 25 - 70 years, mean NR | Both genders reported fear-based barriers (although these barriers were more salient in girls), including concerns about not being believed, safety concerns (due to perpetrators threatening violence), concerns about negative impacts on family, and concerns that family members may respond by harming perpetrators. Both genders also reported non-disclosure arising from desires to protect (avoid upsetting) family members. Shame-related barriers were more salient in boys and included shame surrounding sexuality (i.e., belief they were targeted by men because they seemed gay; shame associated with “becoming” or being labelled gay) and internalised blame. | N/A – not assessed |
| Jackson et al.; 2017; United States and Canada | ST-exposed men who had sex with men; n = 18; all men; range NR; *M* = 42.2 years | Difficulty trusting others (discussed in relation to sexual minority status; impacted by historical tensions with police and by negative experiences 'coming out' to supports), concerns about unsupportive responses and disbelief (related to stereotypes about men who had sex with men as "promiscuous") and double stigma associated with being assaulted as both men and sexual minority men, unsupportive past responses to disclosures from informal and formal supports (dismissed, disbelieved, asked unnecessary probing questions, job loss and other tangible consequences, lack of follow-up after disclosures), lack of understanding of the experiences and needs of men who had sex with men among practitioners. | Disclosure deemed safer with people who men had 'come out' to, past supportive/positive responses to disclosures facilitated subsequent disclosures. |
| Jamel, Bull, & Sheridan; 2008; United Kingdom | Sexual assault service users (in Australia) and police (in United Kingdom); n = 95; ST-exposed: n = 76, mixed gender (n = 20 men), range and mean NR; police: n = 19, gender NR, range and mean NR | Barriers reported by both genders included concerns about not being believed, not having sufficient evidence to pursue legal action, self-blame, shame and humiliation, concerns about negative police responses (particularly with known perpetrators), safety concerns (perpetrator retaliation), and confidentiality concerns. Women more often reported feeling embarrassed by the event (not reported by men) and men reported concerns their sexuality would be “outed”, concerns about not being believed (particularly with perpetrators in positions of authority), and perceived futility in reporting due to poor faith in police responsiveness. | Desire for retribution/legal action (both men and women); men also reported to seek referrals to appropriate agencies (as they did not know where to go for help) and to receive recognition that the assault was against the law. |
| Jamel; 2010; United Kingdom | Sexual assault service users (in Australia) and police (in United Kingdom); n = 95; ST-exposed: n = 76, mixed gender (n = 20 men), range and mean NR; police: n = 19, gender NR, range and mean NR | Lack of confidence in police and judicial system, uncertainty about where and how to seek support, invisibility of men's ST and lack of resources that represented ST-exposed men, preference to disclose to women police (not wanting to admit how much they had "failed as a man" to men). | Motivators were seeking recognition that the assault was against the law, seeking retribution or legal justice and reporting to find relevant supports. |
| Javaid; 2018; United Kingdom | Mental health practitioners with experience supporting ST-exposed men and police; n = 70; mixed gender (n = 33 men); range and mean NR | Shame, perceived futility in reporting (lack of faith in police/criminal justice system response), concerns about not being believed, and concerns about homophobic and dismissive responses (particularly for men who have sex with men). | N/A – not assessed |
| Manor-Binyamini & Schreiber-Divon; 2023; Israel | ST-exposed Bedouin men; n = 17; all men; range = 23 - 40 years; M = 31.88 years | Shame and confusion around ST experiences, concerns about ostracization and reputational damage in the local community (for self and family), concerns about labelled "weak" or "a woman" and ST as violating masculine norms, silence and taboo surrounding ST experienced by boys and men, negative reactions to disclosure (including disbelief and encouragement to stay silent) | N/A – not assessed |
| Mgolozeli & Duma; 2020; South Africa | Sexual assault service users (post-rape crisis centres); n = 11; all men; age range = 18 - 65, mean NR | Safety concerns (perpetrator retaliation), concerns about negative reactions from police (not being taken seriously, non-responding, laughing, harmful and homophobic comments; particularly reported by gay men), confidentiality concerns, concerns about negative impacts on social standing and professional reputation, perceived futility in reporting (limited faith in justice system, particularly when perpetrators were unknown). | Encouragement from loved ones, safety concerns (perpetrator retaliation), seeking legal justice, preventing others from experiencing ST, concerns about re-victimisation (from the same perpetrator or another perpetrator), seeking answers from perpetrators about ST experience. |
| Oueis, McKie, & Reissing; 2024; Canada | ST-exposed (adulthood) men who had sex with men; n = 206; all men; range = 18 - 77 years; *M* = 31.84 years | Avoidance/minimisation, self-blame, lack of services for ST-exposed men (and services tailored for trans men and men who had sex with men), concerns about reports being dismissed or disbelieved by police, not wanting to go through the difficulty of the criminal justice system, difficulty recognising that experiences constituted assaults (particularly when sexual experiences had initially been consensual). | Hearing others' stories and exposure to conversations about consent in media (both facilitated recognition of own assaults). |
| Pacheco, Buenaventura, & Miles; 2023; International | ST-exposed men (child sexual exploitation and abuse); n = 10; all men; range = 20 - 59 years; mean NR | Difficulty recognising and disclosing abuse informed by manipulation, deception, and gaslighting from perpetrators (including family members involved in exploitation and abuse), desire to protect relationships with family members, taboo associated with discussing incest, conflation between perceiving self as gay (due to physiological responses during abuse) and religious beliefs, further exacerbating shame, negative past experiences with disclosure to police and health practitioners (disbelief, not taking disclosures seriously/separating child from perpetrators, encouraging silence, responding with violence, gender biases where victimisation against boys/men not acknowledged). | N/A – not assessed |
| Patterson et al.; 2023; New Zealand | Peer support group members for ST-exposed men; n = 9; all men; range = 42 - 67 years, mean NR | Avoidance (including attempts at distraction/escape via substance use), shame, self-blame, difficulty labelling experiences as abuse, negative past disclosure experiences with police and informal supports (including lack of response and follow-up), belief the perpetrator loved and cared about them. | N/A – not assessed |
| Petersson & Plantin; 2019; Sweden | ST-exposed men from general community; n = 10; all men; range = "20s to 70s"; mean NR | Shame, internalised blame, minimising experiences, not wanting to be viewed as victims (consequently felt more comfortable discussing ST with people with knowledge about men's ST, such as practitioners and researchers), unsupportive/uncomfortable responses to disclosures of ST perpetrated against men. | Sought therapy to understand and process ST experiences, and in pursuit of improved mental health and wellbeing. |
| Rapsey et al.; 2020; New Zealand | Peer support group members for ST-exposed men; n = 9; all men; range = 42 - 67 years, mean NR | Concerns about unsupportive responses (judgment, ridicule, disbelief), concerns about not being understood or accepted by mental health practitioners, negative previous help-seeking experiences with mental health practitioners (struggling to connect; practitioners not adequately discussing ST in therapy), financial costs of accessing support, referral-related challenges, shame (perceived weakness for needing psychological support), perceived power imbalance in therapy between clients and practitioners. | Seeking self-acceptance and understanding of experiences, seeking more healthy coping mechanisms (with greater sense of control over behaviours and futures), encouraged to seek help by a loved one (partner), therapeutic disclosure was supported by trusting relationships with practitioners. |
| Reeves & Stewart; 2017; Canada | ST-exposed Indigenous men attending a culture-based, multi-health service for Indigenous community members and mental health practitioners and community healers working at the service; n = 16; ST-exposed: n = 6, all men, range = 30 - 60 years, mean NR; support workers: n = 10, mixed gender (n = 5 men), age range="late 20s to late 60s", mean NR | Desire to uphold masculine norms (not wanting to be viewed as weak for seeking support), invisibility of men’s ST, shame, guilt, social isolation, difficulty trusting others, difficulty expressing vulnerability, and lack of appropriate services for men. | Help-seeking often occurred for reasons related to ST but not to discuss ST directly, such as violence, substance use, relationship problems, or worsening mental health (including crisis points). Facilitators were developing trusting relationships with practitioners, encouragement to talk about ST, hearing others’ stories, provision of culturally meaningful care and connection to culture. |
| Roberts; 2020; United Kingdom | Prison-based sample of men who had both experienced and perpetrated CSA; n = 18; all men; range = 23-67 years, mean NR | Shame, safety concerns (perpetrator retaliation or physical violence from disclosure source), concerns about not being believed, fear of legal proceedings negatively impacting family members, desire to uphold masculine norms, social isolation, uncertainty about characterising events as ST (particularly when perpetrated by women), and desire for ongoing relationship with perpetrator. | N/A – not assessed |
| Sharma; 2022; India | ST-exposed men from general community; n = 11; all men; range = "20s to 50s"; mean NR | Shame, guilt and self-blame (including beliefs of inviting abuse and that assaults did not reach threshold of violence to constitute abuse), difficulty labelling experiences as abuse, protecting perpetrators' social standing and reputation, taboo around sex and same-sex sexual acts, concerns about unsupportive responses (that reports would be minimised, dismissed, or ignored) | N/A – not assessed |
| Sivagurunathan et al.; 2019a; Canada | Mental health practitioners with expertise supporting CSA-exposed men; n = 11; mixed gender (n = 4 men); range and mean NR | Shame, guilt and self-blame (particularly with physiological arousal), denial, minimising ST and its impacts, invisibility of men’s ST, sexuality conflicts (whether they invited the abuse or the abuse “made them gay”), sexuality concerns (not wanting to be labelled gay), family members discouraging disclosure, being blamed, concerns about social rejection or loss of relationships, concerns about job loss, concerns about being labelled future perpetrators, desire to uphold masculine norms (both ST and help-seeking as violating masculine norms), lack of trusted supports, concerns about being disbelieved (particularly when perpetrators were trusted community figures), desire to “protect” perpetrators from consequences or punishment, uncertainty about where and how to access support, lack of openness about sex and sexuality in family, ST as taboo (particularly when experienced by boys/men). | Inner strength, disclosure sources providing safe and trusting environments for disclosure (not being judgmental or minimising reports), hearing others' ST experiences and group therapy with other ST-exposed men. |
| Sivagurunathan, Orchard, & Evans; 2019b; Canada | Mental health practitioners with experience supporting CSA-exposed men; n = 11; mixed gender (n = 4 men); range and mean NR | Lack of appropriate services for men, limited choice between individual vs group-based modalities, financial cost, difficulties with transport and service accessibility (particularly for men with disabilities), long waitlists, requiring referrals from other services/practitioners, limited number of sessions, lack of appropriately skilled practitioners and training in men’s ST, language and communication issues (for men with communication-related difficulties and speaking languages other than English), negative previous experiences with mental health practitioners (dismissive attitudes), perceived power imbalance in therapy, limited knowledge about available programs and services (among men and service providers), and poor communication between services. | Practitioners showing vulnerability |
| Sorsoli, Kia-Keating, & Grossman; 2008; United States | Men exposed to family member- or caretaker-perpetrated CSA; n = 20; all men; range = 24-61 years; mean NR | Repressed memories, avoidance, difficulty labelling events as ST, difficulty talking about ST (e.g., initiating discussions, articulating events, describing associated emotions), shame about CSA and non-disclosure, not feeling emotionally ready or safe to disclose, perceived futility in disclosing, concerns about negative reactions (being "sent away" by parents, being labelled perverse, rapists, crazy or gay), not wanting to upset others, lack of trusted supports, negative past disclosure responses (including physical violence), belief that others knew about the CSA but chose not to respond, ST as taboo, invisibility of men's ST. | Being asked directly, encouragement from supports (therapist encouraged disclosure to mother). Motivators included wanting to process experiences and find closure. |
| Turchik et al.; 2013; United States | Veteran men exposed to military sexual trauma who had not received mental health care; n = 20; all men; range NR, *M* = 62.20 years | Shame, self-reliance, internalised blame, confidentiality concerns, minimisation, concerns about healthcare provider responses (shock, disgust, laughing, disbelief, avoiding discussions about ST), beliefs that men should not need help for ST, help-seeking perceived as violating masculine norms, sexuality concerns (not wanting to be labelled gay) uncertainty about where and how to access support. | N/A – not assessed |
| Weare, Hulley, & Craig; 2024; United Kingdom | ST-exposed men (forced-to-penetrate cases with women) from general community; n = 30; range NR; *M* = 42.9 years | Shame, difficulty understanding and labelling ST experiences (particularly with physiological arousal during assaults and due to difficulty recognising women as perpetrators), internalised blame (including beliefs about not sufficiently communicating non-consent), concerns about responses to disclosure (dismissed, disbelieved, laughter, negative appraisals), expectations for men to constantly desire heterosexual sexual encounters, concerns about perpetrator retaliation (counter-claims of rape and abuse), legal definitions did not acknowledge experiences, limited options for support and practitioner knowledge, ST perceived as violating masculine norms | N/A – not assessed |
| Widanaralalage et al.; 2022; United Kingdom | Men exposed to ST perpetrated by other men after age 13; n = 9; all men; range and mean NR | Shame, internalised blame, difficulty labelling ST experiences, desire to uphold masculine norms (not wanting to be labelled weak or victims, particularly among gay men), concerns about disbelief, concerns about being pitied or viewed differently by others, sexuality concerns (not wanting to be labelled gay), negative past help-seeking experiences (unsatisfactory and insensitive police responses; being turned away from sexual assault services), ST as taboo, invisibility of men’s ST. | Motivators were seeking legal justice and moral imperative (civic duty). |
| Young, Pruett, & Colvin; 2018; United States | Sexual assault service users; n = 116; mixed gender (n = 58 boys/men); range = 15-61 years, *M* = 32 years | Men showed greater indications of difficulty trusting helpline workers, relative to women^3^. Helpline workers described men’s discomfort disclosing ST to their social supports (particularly to other men) and difficulty expressing thoughts and feelings surrounding ST. Callers of both genders reported uncertainty about characterising events as ST. Women discussed concerns about being believed more often than men. | For men, motivators for calling the helpline were initial ST disclosures due to difficulty talking to social supports about ST experiences (whereas women more often used the helpline as an adjunct support) and for crisis counselling and referrals. Relative to men, women used the helpline for a broader range of reasons (e.g., drug testing, rape kits, sexually transmitted infection testing, abortion clinics, self-defence training). |
| Zalcberg; 2017; Israel | CSA-exposed members of Haredi (ultra-Orthodox Jewish) community; n = 40; all men; age range = 18 - 44 years, *M* = 29 years | Concerns about being blamed, concerns about lack of response, concerns about being viewed as perverse and negative impacts on social standing, ST as taboo, lack of openness about sexuality in culture, safety concerns (physical violence from disclosure source; perpetrator retaliation), concerns about perpetrator retaliation by telling others in community, desire to protect (avoid upsetting) loved ones, negative previous help-seeking experiences (not taken seriously, lack of response), uncertainty about characterising events as ST. | Hearing others' stories, cultural changes in acceptability of discussing ST. |
| *Note.* ST=sexual trauma. CSA=child sexual abuse. ^1^ Crisis points throughout refer to declining mental ill-health warranting immediate support. ^2^ Recruitment sources (websites for ST-exposed men) were US-based organisations, but survey could be accessed from any country. ^3^ Evidenced by abrupt hang-ups during calls to a sexual assault helpline. | | | |
