# Supplementary material for: Barriers and Facilitators for Sexual Trauma Disclosure in Boys and Men: A Systematic Review
Source: Trauma Violence Abuse. 2025 Mar 23;27(3):830–53. doi: 10.1177/15248380251325210 (PMC13287383; doi:10.1177/15248380251325210)
Supplement: sj-docx-7-tva-10.1177_15248380251325210 – Supplemental material for Barriers and Facilitators for Sexual Trauma Disclosure in Boys and Men: A Systematic Review [file sj-docx-7-tva-10.1177_15248380251325210.docx]

**Supplementary Table C.1.**

*Quality appraisal using the Mixed-Methods Appraisal Tool (2018) for quantitative articles reporting endorsed disclosure barrier items and mixed-methods articles*

| **Article** | **Quality appraisal items in Mixed-Methods Appraisal Tool (2018)** | | | | | **Rating** |
| --- | --- | --- | --- | --- | --- | --- |
|  | **For quantitative articles reporting endorsed disclosure barrier items** | | | | |  |
|  | **Sampling strategy relevant to research question** | **Representative sample of target population** | **Appropriate measurements** | **Risk of non-response bias low** | **Appropriate statistical analysis** |  |
| Adejimi et al., 2016 | YES | NO | CAN'T TELL | CAN'T TELL | YES | Poor |
| Frias & Erviti, 2014 | YES | YES | NO | CAN'T TELL | YES | Fair |
| Hahn et al., 2021 | YES | NO | YES | NO | NO | Poor |
| Lehrer et al., 2013 | NO | NO | NO | CAN'T TELL | NO | Poor |
| Okur et al., 2020 | NO | NO | NO | CAN'T TELL | YES | Poor |
| Postmus et al., 2015 | NO | NO | NO | CAN'T TELL | YES | Poor |
|  | **For mixed-methods articles** | | | | |  |
|  | **Adequate rationale for mixed methods design** | **Different components of the study effectively integrated to answer the research question** | **Integration of qualitative and quantitative components adequately interpreted** | **Addressed divergences and inconsistencies between quantitative and qualitative results** | **Do components adhere to the quality criteria of each tradition of the methods involved** |  |
| Guerra et al., 2021 | YES | NO | YES | YES | NO | Fair |
| Kwon et al., 2007 | NO | NO | YES | YES | NO | Poor |

**Supplementary Table C.2.**

*Quality appraisal using the Joanna Briggs Institute Critical Appraisal Tool for Analytical Cross-Sectional Studies (2020) for quantitative articles examining predictors of disclosure likelihood and timing*

| **Article** | **Quality appraisal items** | | | | | | | | **Rating** |
| --- | --- | --- | --- | --- | --- | --- | --- | --- | --- |
|  | **Clearly defined inclusion criteria** | **Subjects and setting described in detail** | **Exposure measured in valid and reliable way** | **Objective, standard criteria for measuring condition** | **Confounding factors identified** | **Strategies to deal with confounders stated** | **Outcomes measured in reliable and valid way** | **Appropriate statistical analysis** |  |
| Boudreau et al., 2018 | YES | YES | NO | YES | YES | YES | N/A | YES | Good |
| Broban et al., 2020 | YES | NO | N/A | YES | YES | NO | N/A | NO | Fair |
| Canan et al., 2023 | YES | YES | N/A | YES | NO | NO | N/A | NO | Poor |
| Cashmore et al., 2017 | YES | YES | UNCLEAR | YES | NO | NO | NO | NO | Poor |
| Coxell et al., 2000 | NO | YES | N/A | YES | NO | N/A | N/A | NO | Poor |
| Easton, 2013 | YES | YES | N/A | NO | YES | NO | YES | NO | Fair |
| Eisenberg et al., 2021 | YES | YES | N/A | YES | NO | NO | N/A | NO | Poor |
| Gundlapalli et al., 2019 | YES | YES | N/A | YES | NO | NO | NO | YES | Poor |
| Hanson et al., 2003 | YES | NO | NO | YES | YES | YES | N/A | YES | Fair |
| Hershkowitz et al., 2005 | YES | NO | YES | NO | NO | NO | NO | NO | Poor |
| Hietamäki et al., 2024 | YES | YES | N/A | YES | NO | NO | N/A | NO | Poor |
| Masho & Alvanzo, 2010 | YES | YES | YES | YES | YES | YES | N/A | YES | Good |
| Nofziger & Stein, 2006 | YES | YES | YES | NO | YES | YES | N/A | YES | Fair |
| Priebe & Svedin, 2008 | YES | NO | YES | YES | YES | YES | N/A | YES | Good |
| Priebe & Svedin, 2012 | YES | NO | YES | YES | YES | YES | N/A | YES | Good |
| Romano et al., 2019 | YES | YES | YES | YES | YES | NO | N/A | NO | Poor |
| Velloza et al., 2022 | YES | YES | NO | YES | YES | YES | N/A | YES | Good |
| Walfield et al., 2024 | YES | YES | N/A | YES | YES | YES | N/A | YES | Good |
| Weiss, 2010 | YES | YES | N/A | YES | NO | NO | N/A | NO | Poor |
| *Note.* N/A = not applicable. | | | | | | | | | |

**Supplementary Table C.3.**

*Quality appraisal using the Critical Skills Appraisal Program (2018) for qualitative articles*

| **Article** | **Critical Skills Appraisal Program (2018) items** | | | | | | | | | | **Rating** |
| --- | --- | --- | --- | --- | --- | --- | --- | --- | --- | --- | --- |
|  | **Clear statement of aims** | **Qualitative method appropriate** | **Design appropriate to meet aims** | **Recruitment strategy appropriate to aims** | **Collected data in way that addressed research issue** | **Considered relationship between researcher and participants** | **Ethical issues taken into consideration** | **Sufficiently rigorous analysis** | **Clear statement of findings** | **Results help locally** |  |
| Alaggia, 2005 | YES | YES | YES | YES | YES | NO | CAN'T TELL | YES | YES | YES | Good |
| Aspin et al., 2009 | YES | YES | YES | NO | YES | NO | CAN'T TELL | YES | YES | YES | Fair |
| Attrash-Najjar et al., 2023 | YES | YES | YES | NO | YES | CAN’T TELL | YES | YES | YES | YES | Good |
| Braun et al., 2009 | YES | YES | YES | YES | YES | NO | YES | YES | NO | NO | Fair |
| Christian et al., 2011 | YES | YES | NO | NO | YES | NO | YES | YES | YES | YES | Poor |
| Chynoweth et al., 2020 | YES | YES | NO | YES | YES | YES | YES | YES | YES | YES | Fair |
| Corboz et al., 2023 | YES | YES | YES | NO | NO | NO | YES | YES | YES | YES | Fair |
| Donne et al., 2018 | YES | YES | NO | YES | YES | NO | YES | YES | NO | YES | Poor |
| Easton et al., 2014 | YES | YES | YES | YES | YES | NO | YES | YES | YES | YES | Good |
| Elder et al., 2017 | YES | YES | YES | NO | YES | YES | YES | YES | YES | YES | Good |
| Forde & Duvvury, 2017 | YES | YES | YES | NO | YES | NO | YES | YES | YES | YES | Good |
| Foster, 2017a | YES | NO | NO | CAN'T TELL | YES | YES | YES | YES | NO | YES | Poor |
| Foster, 2017b | YES | YES | NO | CAN'T TELL | YES | YES | YES | YES | NO | YES | Poor |
| Gagnier, 2016 | YES | YES | YES | YES | YES | NO | YES | YES | YES | YES | Good |
| Gagnier, 2017 | YES | YES | YES | YES | YES | NO | YES | YES | YES | YES | Good |
| Gill & Begum, 2023 | YES | YES | YES | YES | YES | NO | YES | YES | YES | YES | Good |
| Gruenfeld et al., 2017 | YES | YES | YES | NO | YES | NO | YES | YES | YES | YES | Good |
| Hlavka, 2017 | YES | YES | YES | YES | NO | NO | YES | NO | YES | YES | Fair |
| Holland & Cipriano, 2021 | YES | YES | NO | YES | NO | NO | YES | NO | YES | YES | Poor |
| Hunter, 2011 | YES | YES | YES | YES | NO | NO | YES | CAN'T TELL | NO | YES | Fair |
| Jackson et al., 2017 | YES | YES | YES | YES | YES | YES | YES | YES | YES | YES | Good |
| Jamel et al., 2008 | YES | YES | YES | YES | NO | NO | CAN'T TELL | NO | YES | YES | Fair |
| Jamel et al., 2010 | YES | YES | YES | YES | NO | NO | CAN'T TELL | NO | YES | YES | Fair |
| Javaid, 2018 | YES | YES | NO | YES | YES | NO | CAN'T TELL | NO | YES | NO | Poor |
| Manor-Binyamini & Schreiber-Divon, 2023 | YES | YES | YES | NO | YES | NO | YES | YES | YES | YES | Good |
| Mgolozeli & Duma, 2020 | YES | YES | YES | YES | YES | NO | YES | YES | YES | YES | Good |
| Oueis et al., 2024 | YES | YES | NO | YES | YES | NO | YES | YES | YES | YES | Fair |
| Pacheco et al., 2023 | YES | YES | NO | YES | YES | NO | YES | YES | YES | YES | Fair |
| Patterson et al., 2023 | YES | YES | YES | NO | YES | NO | YES | YES | YES | YES | Good |
| Petersson & Plantin, 2019 | YES | YES | YES | YES | YES | YES | YES | YES | YES | YES | Good |
| Rapsey et al., 2020 | YES | YES | YES | YES | YES | NO | YES | YES | YES | YES | Good |
| Reeves & Stewart, 2017 | YES | YES | YES | YES | YES | NO | YES | YES | YES | YES | Good |
| Roberts, 2020 | YES | YES | NO | NO | YES | NO | YES | YES | YES | YES | Fair |
| Sharma, 2022 | YES | YES | YES | NO | YES | NO | YES | YES | YES | YES | Good |
| Sivagurunathan, 2019a | YES | YES | YES | YES | YES | CAN'T TELL | YES | YES | YES | YES | Good |
| Sivagurunathan, 2019b | YES | YES | YES | YES | YES | CAN'T TELL | YES | YES | YES | YES | Good |
| Sorsoli et al., 2008 | YES | YES | NO | YES | YES | YES | YES | YES | YES | YES | Good |
| Turchik et al., 2013 | YES | YES | YES | YES | YES | NO | YES | NO | YES | YES | Good |
| Weare et al., 2024 | YES | YES | YES | CAN’T TELL | YES | NO | YES | YES | YES | YES | Good |
| Widanaralalage et al., 2022 | YES | YES | YES | YES | NO | CAN'T TELL | YES | YES | YES | YES | Good |
| Young et al., 2018 | YES | NO | YES | YES | YES | CAN'T TELL | YES | NO | YES | YES | Poor |
| Zalcberg, 2017 | YES | YES | YES | YES | YES | NO | YES | YES | YES | YES | Good |
